# Nasopharyngeal carcinoma MHC region deep sequencing identifies *HLA* and novel non-HLA *TRIM31* and *TRIM*39 loci

**DOI:** 10.1038/s42003-020-01487-y

**Published:** 2020-12-11

**Authors:** Lvwen Ning, Josephine Mun-Yee Ko, Valen Zhuoyou Yu, Hoi Yan Ng, Candy King-Chi Chan, Lihua Tao, Shiu-Yeung Lam, Merrin Man-Long Leong, Roger Kai-Cheong Ngan, Dora Lai-Wan Kwong, Anne Wing-Mui Lee, Wai-Tong Ng, Ashley Cheng, Stewart Tung, Victor Ho-Fun Lee, Ka-On Lam, Chung-Kong Kwan, Wing-Sum Li, Stephen Yau, Jin-Xin Bei, Maria Li Lung

**Affiliations:** 1 Department of Clinical Oncology, University of Hong Kong, Hong Kong (Special Administrative Region), People’s Republic of China; 2 Center for Nasopharyngeal Carcinoma Research, University of Hong Kong, Hong Kong (Special Administrative Region), People’s Republic of China; 3 Department of Clinical Oncology, Queen Elizabeth Hospital, Hong Kong (Special Administrative Region), People’s Republic of China; 4 Department of Clinical Oncology, Pamela Youde Nethersole Eastern Hospital, Hong Kong (Special Administrative Region), People’s Republic of China; 5 Department of Oncology, Princess Margaret Hospital, Hong Kong (Special Administrative Region), People’s Republic of China; 6 Department of Clinical Oncology, Tuen Mun Hospital, Hong Kong (Special Administrative Region), People’s Republic of China; 7 Sun Yat-sen University Cancer Centre, State Key Laboratory of Oncology in South China, Collaborative Innovation Centre for Cancer Medicine, Guangdong Key Laboratory of Nasopharyngeal Carcinoma Diagnosis and Therapy, 510060 Guangzhou, People’s Republic of China

**Keywords:** Head and neck cancer, Cancer genomics, Cancer genomics, Immunogenetics

## Abstract

Despite pronounced associations of major histocompatibility complex (MHC) regions with nasopharyngeal carcinoma (NPC), causal variants underlying NPC pathogenesis remain elusive. Our large-scale comprehensive MHC region deep sequencing study of 5689 Hong Kong Chinese identifies eight independent NPC-associated signals and provides mechanistic insight for disrupted transcription factor binding, altering target gene transcription. Two novel protective variants, rs2517664 (*T*_rs2517664_ = 4.6%, *P* = 6.38 × 10^−21^) and rs117495548 (*G*_rs117495548_ = 3.0%, *P* = 4.53 × 10^−13^), map near *TRIM31* and *TRIM39*/*TRIM39-RPP21*; multiple independent protective signals map near *HLA-B* including a previously unreported variant, rs2523589 (*P* = 1.77 × 10^−36^). The rare HLA-B*07:05 allele (OR < 0.015, *P* = 5.83 × 10^−21^) is absent in NPC, but present in controls. The most prevalent haplotype lacks seven independent protective alleles (OR = 1.56) and the one with additional Asian-specific susceptibility rs9391681 allele (OR = 2.66) significantly increased NPC risk. Importantly, this study provides new evidence implicating two non-human leukocyte antigen (HLA) genes, E3 ubiquitin ligases, *TRIM31* and *TRIM39*, impacting innate immune responses, with NPC risk reduction, independent of classical HLA class I/II alleles.

## Abstract (web summary)

Here the authors report a major histocompatibility complex (MHC) association analysis for nasopharyngeal carcinoma in Chinese individuals from Hong Kong, finding 8 independent associated loci associated with lower risk for developing nasopharyngeal carcinoma. Two non-human leukocyte antigen (HLA) genes are E3 ubiquitin ligases, *TRIM31* and *TRIM39*, having a role in the innate immune response and implicating the importance of host Epstein-Barr virus interactions in this cancer.

## Introduction

NPC, an epithelial tumor arising in the nasopharynx, has distinct geographic and ethnic distributions^1^. Global cancer statistics by GLOBOCAN estimated 129,079 new cases and 72,987 deaths in 2018^2^. It is endemic in Southeast Asia, Southern China, particularly in Guangdong and Hong Kong, Taiwan, Northern Africa, and Alaska^1,3^. Locally, NPC poses considerable health burdens and is the most common cancer among males aged 20–44^3^. NPC etiology is multifactorial, including host genetics, Epstein–Barr virus (EBV) infection, and environmental factors^1,4–9^. Utilizing various genomic approaches, we previously demonstrated the contribution of multiple loci including 6p21, *TERT*, and *MST1R* in NPC^5–7^. Numerous NPC genetic susceptibility association studies identified the 6p21.3 human leukocyte antigen (HLA) locus^4,7,8,10–20^, supporting the hypothesis that HLA alleles or nearby non-HLA genes are major genetic determinants for NPC pathogenesis. However, most of these studies were limited by a small sample size^4,8,11–20^. This gene-dense region is highly polymorphic; the extensive linkage disequilibrium of the HLA pattern poses challenges and requires large cohort studies to resolve the underlying genetic causal factors for NPC. Only one genome-wide association study (GWAS) reported three independent signals associated with NPC risk from the MHC region^7^; this array-based approach may be inadequate for fine-mapping of all the independent signals within the MHC region. Hence, we used comprehensive deep sequencing of the entire 5 Mb MHC region in a large cohort of 5689 age-, gender- and ethnicity-matched Hong Kong Chinese in a two-phase study to dissect in detail the genetic causal factors underlying NPC pathogenesis. To the best of our knowledge, the current study is the largest comprehensive fine-mapping analysis at the single-base resolution to systematically resolve eight independent loci within the MHC region associated with NPC. Our data validated earlier well-known NPC GWAS findings on the HLA-A alleles and amino acids polymorphisms and, additionally, detected six independent loci at the *HLA-A/B* region and *HLA-DQ alpha chain 1* (*HLA-DQA1*)^7,10^. This is the first report of the association of *Tripartite motif-containing 31* (*TRIM31)* with NPC risk and survival. The SNP rs117495548, mapped to a region with two naturally occurring transcripts at *Tripartite motif-containing 39* (*TRIM39*) and a read-through transcript *TRIM39-Ribonuclease P/MRP 21* *kDa subunit* (*RPP21*), associated with reduced NPC risk. This study now provides important novel insights of putative functional SNPs disrupting transcription factor binding, altering target gene transcription for NPC pathogenesis, highlighting the possible involvement of E3 ubiquitin ligases, *TRIM31, TRIM39*, and *TRIM39-RPP21* fusion protein impacting the host innate immunity antiviral defense and for this EBV-associated cancer. Our findings on the haplotype information and genetic heterogeneity of MHC NPC susceptibility loci across East Asian and other populations partially explain the fact that despite ubiquitous EBV infection, worldwide NPC incidence is low, while it is especially high among East Asians and Southern Chinese.

## Results

### Association of common variants, HLA class I alleles, and amino acids with NPC

Owing to the genetic complexity in the MHC region, earlier studies recognized how challenging it was to identify all the independent loci. Hence, we performed fine-mapping by a target-capture sequencing approach with 5689 genetic- and geographically matched Hong Kong Chinese for association analyses after stringent quality control steps. Demographic information of study populations used is detailed in Supplementary Table 1. Our study workflow and analysis pipeline are detailed in Supplementary Fig. 1. The discovery and validation phases included 3047 participants (1431 NPC cases and 1616 controls) and 2642 participants (1321 NPC cases and 1321 controls). The MHC region was sequenced to a mean coverage of 150×. Logistic regression association identified 1971 significant common SNPs/Indels in discovery, validation, and combined phases from 37,931 common variants (*P* < 5 × 10^−8^). Forward logistic regression association analysis of the SNPs/Indels identified five independent signals within the MHC class I region that reached genome-wide significance (Supplementary Table 2). The most significant and the only susceptibility-associated signal was rs9391681 (OR = 2.11, *P* = 1.50 × 10^−46^), located in the intergenic region between *RPP21* and *HLA-E*, followed by four protective signals near *HLA-A*, *HLA-B*, *TRIM31*, and *HCG27* loci. The novel independent signal near *TRIM31* represented by rs2517664 (OR = 0.29, *P* = 6.38 × 10^−21^) was interesting, as it remained independent from HLA class I alleles and amino acid variants in the subsequent conditional logistic analysis.

Our current study identifies 53 *HLA-A* alleles, 98 *HLA-B* alleles, and 39 *HLA-C* alleles. Among the 190 HLA class I alleles, we confirmed NPC risk association for seven common *HLA-A/B/C* alleles (HLA-A*11:01, HLA-A*02:07, HLA-B*46:01, HLA-B*55:02, HLA-B*13:01, HLA-C*01:02, and HLA-C*03:04, Supplementary Table 3). Individuals carrying homozygous susceptibility alleles of HLA-B*46:01, HLA-A*02:07, and heterozygous HLA-B*46:01/HLA-B*51:01 combination conferred ~3-fold higher risk of NPC (Supplementary Table 4). The top NPC-associated haplotype was HLA-A*02:07-HLA-B*46:01-HLA-C*01:02 with modest effect (OR = 2.44, *P* = 7.65 × 10^−27^, Supplementary Table 5).

The association of HLA-A amino acid replicated previous findings with strongest signals at HLA-A_aa-62_ (*P* = 1.48 × 10^−56^) and HLA-A_aa-99_ (*P* = 1.26 × 10^−50^) in the peptide-binding groove supportive for their causal role in the *HLA-A* region (Supplementary Figs. 2, 3 and Table 6)^10–12^. Compared to the Guangxi/Guangdong and Malaysia studies, the current Hong Kong NPC cohorts have very strong additional associations detected at HLA-B_aa-66_ (*P* = 1.21 × 10^−47^), HLA-B_aa-69_ (*P* = 2.24 × 10^−47^), and HLA-B_aa-156_ (*P* = 8.03 × 10^−52^) and relatively weaker signals at HLA-C_aa-6_, HLA-C_aa-9_, and HLA-C_aa-99_^10,11^. Their effects were driven by the HLA-B*46:01 allele, since there were no or marked reduction of significant effects after conditional analysis adjusted with HLA-B*46:01 (Supplementary Table 7).

### Multiple novel independent signals in the MHC region confer NPC risk reduction

**Fig. 1:**
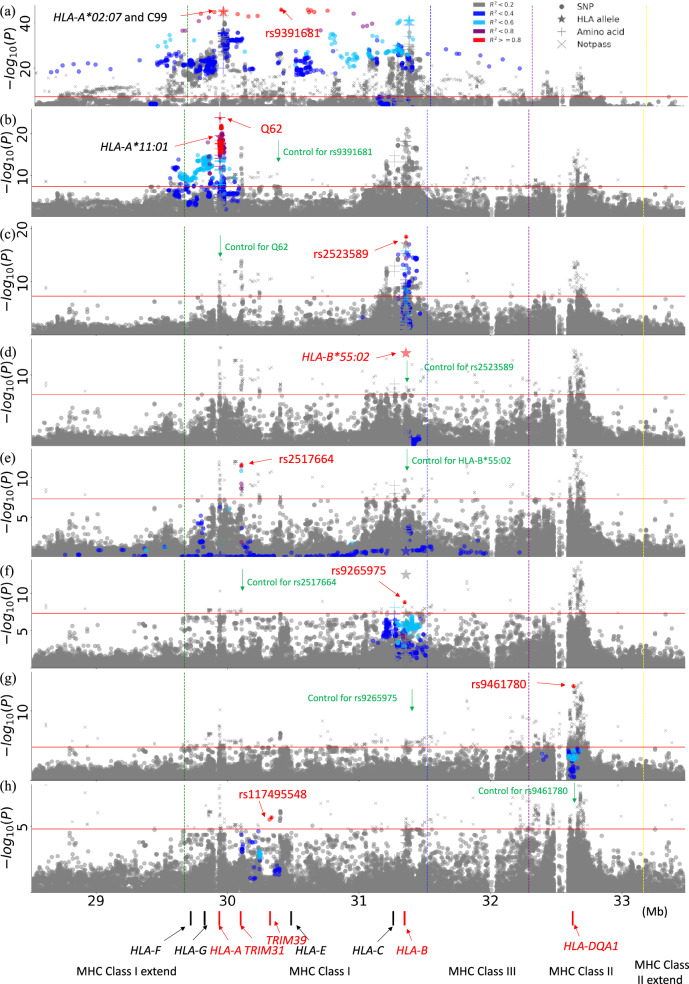
Manhattan plots of the forward conditional association of the variants in HLA region for NPC risk. **a** This panel shows the original association. Other panels show the association while controlling the locus **b** rs9391681, **c** HLA-A_aa-Q62_, **d** rs2523589, **e** HLA-B*55:02, **f** rs2517664, **g** rs9461780, **h** rs117495548. The horizontal axis shows the genomic position and the vertical axis shows negative log_10_-transformed *P* for the association. The red horizontal line corresponds to the significance threshold of *P* = 5 × 10^−8^. SNPs (dots), HLA alleles (stars), and amino acid polymorphisms (+) are marked in the plots. The color of the variant represents the *r*^2^ between this variant and the controlled variant.

**Fig. 2:**
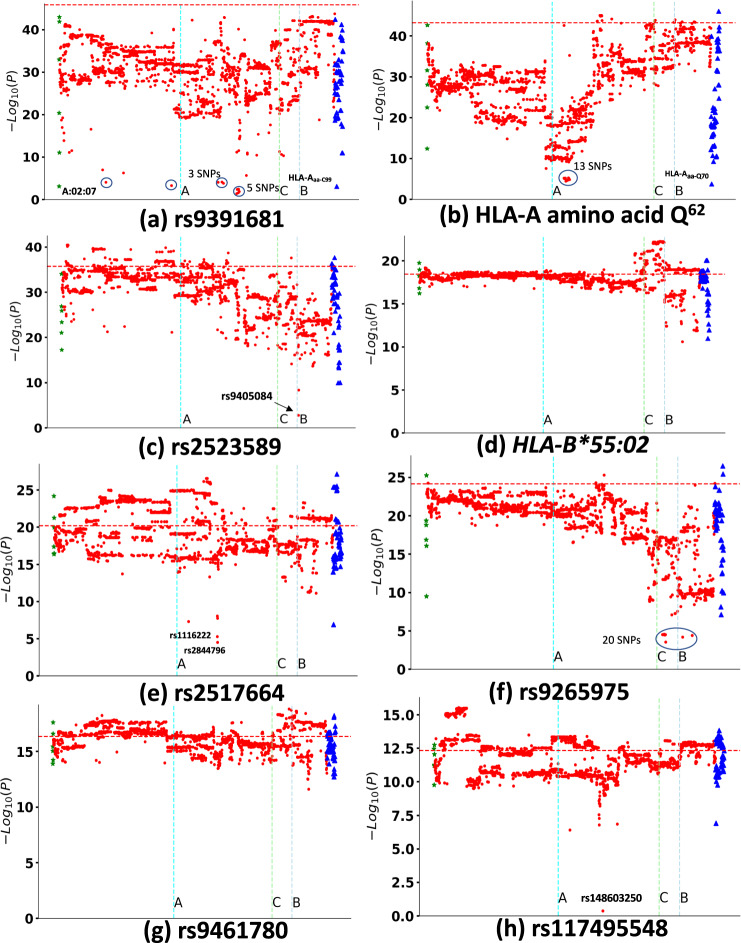
Proxy variant analysis for the eight independent variants adjusted for age and gender. Linkage disequilibrium-proxies reduce markedly the −log_10_(*P*) of the index variants **a** rs9391681, **b** HLA-A_aa-Q62_, **c** rs2523589, **d** HLA-B*55:02, **e** rs2517664, **f** rs9265975, **g** rs9461780, **h** rs117495548. Significantly reduced SNPs are circled. (Details in Supplementary Data 1 for 2967 variants after controlling the eight index variants.) Conditional logistic regression analysis reveals the residual association effect of eight independent signals after adjustment with 2967 significant variants, which includes 2899 SNPs/Indels (red dots), 62 amino acid variants (blue triangles), and 6 HLA alleles (green stars). The *X* axis shows the covariant and the *Y* axis shows the adjusted −log_10_(*P*) for the independent variants. The horizontal red dotted line indicates the unadjusted −log_10_(*P*) of the index variant. SNPs were sorted according to chromosomal coordinates and vertical light blue, green, and gray dotted lines show the location of *HLA-A/B/C*.

Overall, 2967 significant common variants including 2899 SNPs/Indels, 62 amino acid polymorphisms, and 6 HLA alleles are associated with NPC after the Bonferroni correction (*P* < 1.33 × 10^−6^) (Supplementary Data 1). Forward conditional logistic regression analysis was performed to resolve independent representative signals. This fine-mapping approach now clearly demonstrates eight independent signals from the MHC region associated with NPC risk (Table 1). After LD pruning, forward selection stepwise regression analysis with 226 significant SNPs (*r*^2^ > 0.8) identified similar findings of eight independent signals associated with NPC (Supplementary Table 8). Our study is the first to report five independent signals near *HLA-A/B*. Figure 1 illustrates the Manhattan plots of each stepwise analysis. The strongest susceptibility signal, rs9391681 (intergenic RPP21*HLA-E) (*P* = 1.50 × 10^−46^, OR = 2.11), may indirectly represent the causal signals driven by the HLA-A*02:07 allele, which is in tight linkage disequilibrium with rs9391681 (*r*^2^ = 0.85). The effect of rs9391681 is markedly reduced (*P* = 7.59 × 10^−3^), after adjusting with the covariant of HLA-A_aa-C99_ segregating with the HLA-A*02:07 allele (*r*^2^ = 1) (Supplementary Tables 6 and 7). Figure 1a shows ten additional SNPs in tight linkage disequilibrium with rs9391681 (r^2^ ≥ 0.8). The proxy variant analysis in Fig. 2a further indicates the marked reduction of their effect to a very weak association (*P* = 3.63 × 10^−2^–7.41 × 10^−5^, Supplementary Data 1). Although it is biologically plausible to suggest HLA-A_aa-C99_ is the causal factor marked by rs9391681, we cannot rule out the possibilities of these ten tight linkage disequilibrium variants exerting their effects on NPC. Functional genomics data based on the transcription factor chromatin immunoprecipitation and sequencing (ChIP-Seq) Clusters from the ENCODE database prioritized four candidate functional variants (rs143982339 near *ABCF1*, rs9348841 near *PPP1R10*, rs9380181 intergenic between ABCF1*PPP1R10, and rs9380182 near *FLOT1*) (Supplementary Data 1). Noteworthy, *POLR2A* was the common transcription factor involved in all four of these. Recent GWAS consistently reported the HLA-A_aa-Q62_ marked by HLA-A*11:01, rather than HLA-A*02:07, is the causal factor^7,10,11^. We now confirm HLA-A_aa-Q62_ (*P*_HLA-A-aa-Q62_ = 6.27 × 10^−44^, OR = 0.56) as the second independent protective signal (Table 1), which is in tight linkage disequilibrium (*r*^2^ = 0.86) with our previously reported GWAS SNP rs2860580 ~3 kb apart from *HLA-A* (Figs. 1b, 2b and Supplementary Table 6)^7^. The effect of rs2860580 disappears (unadjusted *P* = 1.64 × 10^−37^ to adjusted *P* = 0.14) after adjustment with HLA-A_aa-Q62_ (Supplementary Table 7).

**Table 1:** Association results of eight independent variants of NPC genetic susceptibility in discovery, validation, and combined phases.

Variants	Locus	Markers in LD	EA/OA	MAF	Discovery (*n* = 3047)		Validation (*n* = 2642)		Combined (*n* = 5689)		Conditional analysis
					OR (95% CI)	*P*	OR (95% CI)	*P*	OR (95% CI)	*P*	*P*
rs9391681	*intergenic* RPP21* *HLA-E*	HLA-A*02:07^14,16,17^, HLA-A_aa-C99_^11^	C/T	0.17/0.23/0.12	2.30 (2.00–2.64)	1.73E−31	1.90 (1.64–2.21)	3.68e−17	**2.11** **(1.90–2.33)**	**1.50e−46**	–
HLA-A amino acid Q_62_	*HLA-A*	HLA-A*11:01^7,10,11^, rs2860580^7^	P/Ab	0.33/0.26/0.39	0.54 (0.48–0.61)	1.35E−26	0.58 (0.51–0.65)	7.11e−19	**0.56** **(0.51–0.61)**	**6.27e−44**	**1.68e−24**
rs2523589	*HLA-B*	rs9405084^a^	G/T	0.30/0.24/0.35	0.56 (0.50–0.63)	1.05E−23	0.62 (0.55–0.70)	4.15e−14	**0.59** **(0.54–0.64)**	**1.77e−36**	**3.26e−19**
HLA-B*55:02	*HLA-B*	–	P/Ab	0.018/0.0047/0.031	0.13 (0.070–0.23)	1.99E−11	0.18 (0.10–0.32)	4.81e−09	**0.15** **(0.10–0.23)**	**3.59e−19**	**8.79e−14**
rs2517664	*TRIM31*	rs1116222, rs2844796^a^	T/C	0.030/0.014/0.046	0.27 (0.19–0.39)	7.77E−13	0.31 (0.21–0.45)	1.66e−09	**0.29** **(0.22–0.37)**	**6.38e−21**	**2.55e−12**
rs9265975	*HLA-B*	rs2498207^7^	A/G	0.10/0.072/0.13	0.46 (0.39–0.55)	4.41E−17	0.56 (0.47–0.68)	1.15e−09	**0.51** **(0.45–0.58)**	**6.77e−25**	**1.61e−09**
rs9461780	*HLA-DQA1*	–	T/C	0.071/0.049/0.091	0.51 (0.42–0.62)	2.81E−11	0.55 (0.44–0.70)	6.85e−07	**0.52** **(0.45–0.61)**	**4.38e−17**	**2.60e−14**
rs117495548	*TRIM39/* *TRIM39-RPP21*	rs148603250^a^	G/A	0.020/0.0094/0.030	0.36 (0.24–0.54)	8.56E−07	0.27 (0.16–0.43)	1.19e−07	**0.31** **(0.23–0.43)**	**4.53e−13**	**2.24e−06**

*P* < 1.69e10-5 after Bonferroni correction considering 2967 significant common variants.

OR and *P* of the combined data set and *P* of conditional analysis are bolded.

*EA* effect allele, *OA* other allele, *P* present allele, *Ab* absent allele for locus with multiple alleles, *MAF* minor allele frequency for combined data set/case/control, *conditional analysis* forward conditional logistic regression model with gender- and age-adjusted, *OR* odds ratio, *P* *P-*value.

^a^Putative functional annotated SNPs in tight LD with the previously unreported independent signals observed in the current NPC study.

Among the three independent signals related to *HLA-B* locus, the most interesting is the novel independent protective signal, rs2523589 (chr6:31,359,557, hg38) located ~2.4 kb upstream of *HLA-B* (*P* = 1.77 × 10^−36^, OR = 0.59), as it provides mechanistic insights for NPC genetic epidemiology. The effect of rs2523589 remains significant after adjusting with all significant variants (Figs. 1c and 2c). The effect of rs2523589 remains independent of rs2894207, previous GWAS independent signal at *HLA-B/C* loci, ~64 kb from rs2523589 (*r*^2^ = 0.19) (adjusted *P* = 4.47 × 10^−19^) (Supplementary Table 7). However, its effect diminishes to a very weak association (*P* = 1.58 × 10^−3^, Supplementary Data 1) after adjusting with rs9405084, which is ~1.1 kb apart from rs2523589 (*r*^2^ = 0.91). Functional annotation suggests rs9405084 may influence epigenetic regulation as a transcriptional enhancer with high H3K27Ac histone modification and DNase I enzyme hypersensitivity. Transcription factor (ChIP-Seq) Clusters from the ENCODE database suggest rs9405084 may regulate transcription of target genes through disruption of transcription factor binding. The eQTL evidence according to GTEx suggests the possible impact of rs2523589 on expression levels of multiple candidate genes including *MIR6891, HCG27, HLA-B, HLA-C*, and *MICA*, etc. (Supplementary Table 9).

The second independent protective signal near *HLB-B* loci is rs9265975 (chr6:31,348,114, hg38) (*P* = 6.77 × 10^−25^, OR = 0.51) (Table 1 and Figs. 1f, 2f). It is ~6 kb downstream of *HLA-B*, ~11 kb from rs2523589, and ~52 kb from the rs2894207 reported in previous GWAS. Our study validated the association signal of rs2894207 (Supplementary Table 10). The protective signal associated with rs9265975 is not previously reported in NPC studies and linked with rs2894207 (*r*^2^ = 0.51) (*P* = 2.69 × 10^−5^, Supplementary Data 1) (Supplementary Table 7). However, its effect diminishes to a very weak association (*P* = 2.82 × 10^−4^–2.69 × 10^−5^, Supplementary Data 1) after adjusting with 20 intronic SNPs (*r*^2^ ≥ 0.5). Functional annotation indicates the putative regulatory function for two of these, rs3906273 and rs9468928, near *HLA-B*, mapped to transcription factor *EP300* by the transcription factor ChIP-Seq Clusters from the ENCODE database. The eQTL evidence according to GTEx suggests the possible impact of rs9265975 on expression levels of multiple candidate genes including *LINC01149, HCG27, CCHCR1*, and *HLA-C*, etc. (Supplementary Table 9). HLA-B*55:02 (*P* = 3.59 × 10^−19^, OR = 0.15), the third signal in the *HLA-B* region, was previously reported in Guangxi and Guangdong^10^ and is now confirmed in the Hong Kong population as an independent signal (Figs. 1d, 2d and Supplementary Table 7).

When we conditioned on the rs9391681, HLA-A_aa-Q62,_ rs2523589, and HLA-B*55:02, we further identified the novel independent intronic signal, rs2517664, mapped to a non-HLA gene, *TRIM31* (*P* = 6.38 × 10^−21^, OR = 0.29, Table 1). The association of rs2517664 is independent of *HLA-A* and *HLA-B* signals since the adjusted *P-*value of this index variant remained significant after conditional analysis adjustment with all the significant variants (Figs. 1e, 2e and Supplementary Table 7). Its effect diminishes to a very weak association with two tightly linked putative functional SNPs, rs2844796 (*r*^2^ = 0.62) and rs1116222 (*r*^2^ = 0.58) (*P* = 5.01 × 10^−6^ or 3.16 × 10^−5^, Supplementary Data 1). Hence, this may be driven by two nearby functional epigenetically regulated SNPs, rs1116222 (~2.4 kb apart from rs2517664 located within a CpG island) and rs2844796 (0.75 kb apart from rs2517664 mapping to a CTCF-binding site) according to USCC Genome Browser. Transcription factor ChIP-Seq Clusters from the ENCODE database further suggest rs1116222 may be involved in transcription factor binding of dozens of transcription factors. It may influence epigenetic regulation as a transcriptional enhancer with high H3K27Ac histone modification and DNase I enzyme hypersensitivity. Stratification analysis of a subset of samples, excluding individuals with HLA-A*02:07 or HLA-A*11:01 alleles (964 cases/1053 controls), identified rs2517664 as a significantly associated variant (*P* = 4.91×10^−12^, OR = 0.30). Independent validation of the genetic susceptibility role of *TRIM31* loci by rs2844796 genotyping was performed in our previous NPC GWAS with another Southern Chinese cohort of 3040 controls and 1583 NPC from Guangdong^7^. Logistic regression analysis indicates that rs2844796, which is in the same linkage disequilibrium with rs2517664 (*r*^2^ = 0.62), significantly associates with NPC (MAF_control_ = 0.046, MAF_case_ = 0.025 *P* = 1.62 × 10^−6^, OR 95% CI = 0.54 0.42–0.69).

Conditioned on the first six independent signals, we identified the seventh independent signal at intronic SNP rs9461780 near *HLA-DQA1* at HLA class II region (*P* = 4.38 × 10^−17^; OR = 0.52) (Table 1 and Fig. 1g). Our current study did not replicate the independent finding at rs28421666 near *HLA-DQ/DR* (*P* = 4.15 × 10^−4^, OR = 0.84) reported by previous GWAS (Supplementary Table 10)^7^. The rs24821666 is ~6.3 kb apart from rs9461780, but they are not tightly linked (*r*^2^ = 0.013). The effect of rs9461780 remains strong after adjusted with other significant variants (Fig. 2g).

The last independent signal identified after conditioning on the top seven independent signals, rs117495548, is novel and maps near *TRIM39* or *TRIM39-RPP21* (*P* = 4.53 × 10^−13^, OR = 0.31, Table 1, Fig. 1h). The effect of rs117495548 remains strong after adjusted with other top significant variants (Fig. 2h). However, there is no significant association after controlling with rs148603250 near *HCG17/HCG18*, in tight linkage disequilibrium (*r*^2^ = 0.99) with rs117495548. Transcription factor (ChIP-Seq) Clusters from the ENCODE database further suggest rs148603250 may be involved in transcription factor binding of *RNA Binding Fox-1 Homolog 2* (*RBFOX2*). *RBFOX2* is a key regulator of alternative exon splicing. Interestingly, this locus is annotated with two overlapping transcripts. The first is *TRIM39*, which encodes an E3 ubiquitin ligase involved in apoptosis or cell cycle regulation^21,22^. *TRIM39-RPP21* is a read-through transcript encoded by a naturally occurring fusion product of neighboring *TRIM39* and *RPP21* that regulates type I interferon response and antiviral defense^23^. Independent validation of the genetic susceptibility role of *TRIM39* or *TRIM39-RPP21* loci was performed by the imputation of rs117495548 in our previous NPC GWAS with another Southern Chinese cohort of 3040 controls and 1583 NPC from Guangdong^7^. Logistic regression analysis indicates that rs117495548 significantly associates with NPC (MAF_Control_ = 0.022, MAF_Case_ = 0.014, *P* = 6.83 × 10^−3^, OR 95% CI = 0.62 0.44–0.88).

### Gene-based association test

The gene-based association test by SKAT analysis was performed with all variants including 5% (5506) exonic variants and 95% intronic or intergenic variants. The parameters for weighting more on rare variants by Rvtests are detailed in the methods section. The top 15 candidate genes associated with NPC are summarized in Supplementary Table 11. The role of *TRIM31* (*P* = 6.75 × 10^−29^)*, HLA-B* (*P* = 1.60 × 10^−27^), and *TRIM39* (*P* = 1.47 × 10^−16^) in genetic susceptibility was further supported by gene-level association tests for NPC risk.

### Haplotype analysis defined by the eight NPC independent signals

Haplotype association defined by the eight independent signals including six SNPs, HLA-A_aa-Q62_ and HLA-B*55:02, identifies 24/111 haplotypes significantly (OR2, normalized to H1 haplotype) associated with NPC in Hong Kong Chinese (Table 2). Noteworthy, Hong Kong Chinese individuals carrying the most common H1 haplotype, which lacks all seven protective alleles (0/7), is associated significantly with a higher risk of NPC (OR1 = 1.56, *P* = 2.90 × 10^−27^). An additional susceptibility haplotype, H2 with the risk allele of rs9391681 (OR1 = 2.66, *P* = 1.76 × 10^−53^), also associates with higher NPC risk. The haplotype association (OR2) identified 22 protective haplotypes that confer significant protection for NPC to variable extents depending upon the combinations of protective alleles (Table 2). Ten strong effect haplotypes (H9-H11, H13-H14, H17-H18, H20, and H22-H23) carrying from one to four protective alleles with at least one allele from HLA-B*55:02 or rs2517664 or rs117495548 conferring 5.6- to 16.7-fold reduction of NPC risk relative to H1. The majority of the remaining twelve moderate effect haplotypes carrying one to three MHC class I/II protective alleles of HLA-A_aa-Q62_ or *HLA-B* loci (rs9265975/rs2523589) or *HLA-DQA1* locus (rs9461780) confer 1.6- to 3.5-fold reduction of NPC risk relative to H1.

**Table 2:** Association of haplotypes defined by eight independent signals with NPC in Hong Kong Chinese.

Haplotype (H)^a^	rs9391681	HLA-A_aa-Q62_	rs2523589	HLA-B*55:02	rs2517664	rs9265975	rs9461780	rs117495548	Case_H_ freq	Control_H_ freq	Total_H_ freq	OR1	*P1*	OR2	*P2*
**H1**	**0**	**0**	**0**	**0**	**0**	**0**	**0**	**0**	**0.3530**	**0.2594**	**0.3047**	**1.56**	**2.90e−27**	**1.00**	**1.00e**+**00**
H2	1	0	0	0	0	0	0	0	0.1533	0.0638	0.1071	2.66	1.76e−53	**1.77**	9.18e−16
H3	0	1	1	0	0	0	0	0	0.0434	0.0785	0.0615	0.53	1.01e−14	**0.41**	5.61e−26
H4	0	1	1	0	0	1	0	0	0.0109	0.0277	0.0196	0.39	1.44e−10	**0.29**	4.59e−17
H5	0	1	0	0	0	0	0	0	0.1428	0.1648	0.1542	0.84	1.28e−03	**0.64**	2.10e−14
H6	0	0	1	0	0	1	0	0	0.0164	0.0300	0.0234	0.54	2.14e−06	**0.40**	3.78e−12
H7	0	1	0	0	0	0	1	0	0.0091	0.0192	0.0143	0.47	7.62e−06	**0.35**	3.37e−10
H8	0	0	0	0	0	0	1	0	0.0165	0.0279	0.0224	0.59	5.41e−05	**0.44**	4.47e−10
H9	0	1	1	1	0	0	0	0	0.0005	0.0060	0.0033	0.09	1.00e−07	**0.07**	7.87e−10
H10	0	0	1	1	0	0	0	0	0.0016	0.0068	0.0043	0.24	4.72e−05	**0.18**	2.97e−07
H11	0	1	0	0	1	0	0	0	0.0015	0.0065	0.0040	0.22	4.79e−05	**0.17**	3.55e−07
H12	0	0	1	0	0	0	0	0	0.1161	0.1171	0.1166	0.99	8.87e−01	**0.73**	1.12e−06
H13	0	0	0	1	0	0	0	0	0.0009	0.0049	0.0030	0.18	1.68e−04	**0.14**	3.25e−06
H14	0	1	1	0	1	1	0	0	0.0004	0.0036	0.0020	0.10	7.72e−05	**0.07**	4.29e−06
H15	0	1	0	0	0	1	0	0	0.0102	0.0160	0.0132	0.63	8.25e−03	**0.47**	9.51e−06
H16	0	1	1	0	0	0	1	0	0.0031	0.0077	0.0054	0.40	1.46e−03	**0.30**	1.26e−05
H17	0	0	1	0	0	0	0	1	0.0005	0.0034	0.0020	0.16	5.54e−04	**0.12**	2.83e−05
H18	0	1	0	1	0	0	0	0	0.0007	0.0037	0.0023	0.19	6.15e−04	**0.14**	3.06e−05
H19	0	0	0	0	0	0	0	1	0.0036	0.0078	0.0058	0.46	4.76e−03	**0.34**	5.26e−05
H20	0	0	0	0	1	0	0	1	0.0000	0.0019	0.0010	<0.097	1.03e−03	**<0.071**	1.21e−04
H21	0	0	1	0	0	0	1	0	0.0067	0.0109	0.0089	0.61	2.31e−02	**0.45**	1.65e−04
H22	0	0	1	0	1	1	0	0	0.0002	0.0022	0.0012	0.08	1.95e−03	**0.06**	1.95e−04
H23	0	1	1	0	0	0	0	1	0.0002	0.0022	0.0012	0.08	1.95e−03	**0.06**	1.95e−04
H24	0	1	1	0	1	0	0	0	0.0033	0.0068	0.0051	0.48	1.18e−02	**0.35**	2.44e−04

Twenty-four out of 111 haplotypes were significantly associated with NPC passing Bonferroni correction (*p* < 4.50 × 10^−4^). H1 (bolded) indicates individuals predisposed with higher NPC risk when carrying this susceptibility haplotype lacking all seven protective alleles.

OR1 is the raw odds ratio of each haplotype. OR2 (bolded) is the normalized odds ratio relative to H1, which is the most frequent haplotype in Hong Kong Chinese.

^a^1 stands for the risk C-allele of rs9391681 in H2 and the other seven protective alleles (HLA-A_aa-Q62_, T-allele of rs2517664, A-allele of rs9265975, G-allele of rs2523589, HLA-B*55:02, T-allele of rs9461780, and G-allele of rs117495548) in other haplotypes.

### Genetic heterogeneity of MHC NPC susceptibility loci across East Asian and other populations

**Fig. 3:**
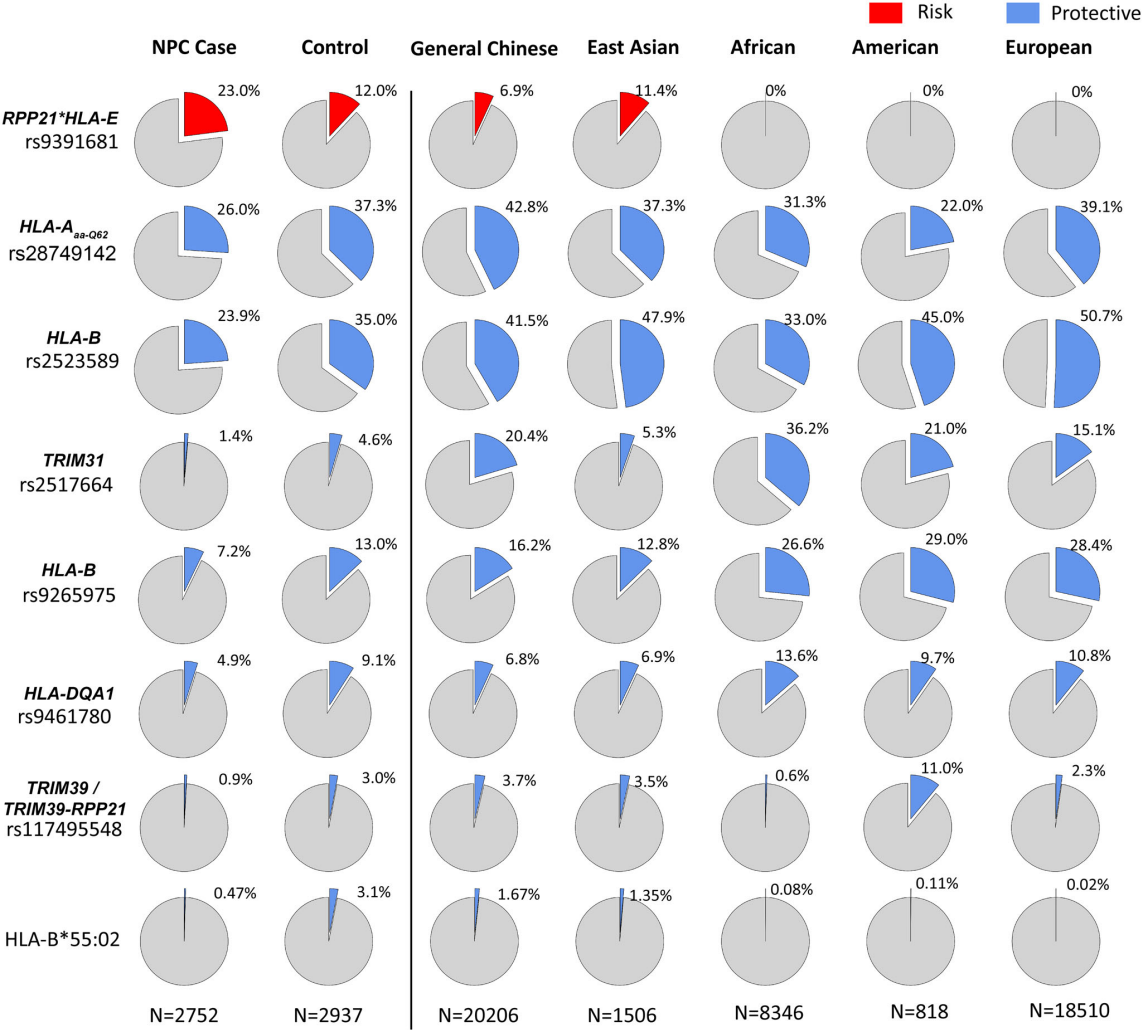
Comparison of the MAF of eight independent associated signals in NPC across different ethnic populations. The other ethnic populations included East Asian, African, American, European, and Ashkenazi Jews from the gnomAD database and a general Chinese cohort from a psoriasis study^25^. The NPC susceptibility allele is in red, while the multiple NPC protective alleles are in blue. The MAF of the susceptibility rs9391681 for NPC is higher in Hong Kong Chinese and East Asians but extremely rare in the four other non-Asian populations from the gnomAD database. The MAFs of rs2523589, rs9265975, rs2517644, rs9461780, and rs117495548 for NPC are much lower in Hong Kong Chinese and East Asians than other non-Asian populations. Rs28749142 is a tagging SNP of HLA-A_aa-Q62_ (*R*^2^ = 0.95)_._ The MAF of rs28749142 does not occur as often in Hong Kong Chinese and East Asians, as compared to general Chinese and European populations. HLA-B*55:02 allele frequencies are obtained from the Allele frequency net database (http://www.allelefrequencies.net) for European, North American, South East Asian populations. HLA-B*55:02 allele is more prevalent in Chinese and East Asian populations.

The eight independent NPC variant frequency distributions were compared across different populations using public database gnomAD^24^, Allele frequency net database (http://www.allelefrequencies.net), and a large general Chinese population from a psoriasis study utilizing MHC-target sequencing (Fig. 3)^25^. Importantly, the most significant NPC susceptibility C-allele of rs9391681 is specific to and only observed in East Asian populations. Hong Kong NPC cases have the highest risk allele frequency of 23%, compared to MAF of 12% of Hong Kong healthy, 11.4% of East Asians, dropping to 6.9% of Northern Chinese and further decreasing to 0% of other non-Asian populations including African, American and European. The protective T-allele of rs2517664 of *TRIM31* locus was only observed in 1.4% NPC cases compared to the 15.1% − 36.2% in populations with low-NPC incidence. The T-allele is ~10 to 26-fold higher among Northern Chinese, African, American, and European populations with low-NPC incidence compared to that of Hong Kong NPC cases. The other five protective alleles of rs28749142 tagging SNP of HLA-A_aa-Q62_, rs2523589 upstream of *HLA-B*, rs9265975 downstream of *HLA-B*, rs9461780 near *TRIM39,* and rs117495548 near *HLA-DQA1* are also more frequent in other low-NPC incidence populations. The Hong Kong Southern Chinese controls further demonstrate a lower frequency of protective alleles of rs28749142, rs2523589, rs2517664, rs9265975, and rs117495548 compared to the general Chinese population^25^; this data set has a large proportion of Northern Chinese, for whom NPC incidence is much lower than in Hong Kong. The differential allelic data between Hong Kong Chinese and East Asians compared to non-Asian populations are concordant with the endemically high NPC incidence in both Hong Kong and East Asia.

### Clinical relevance of TRIM31 in NPC

**Fig. 4:**
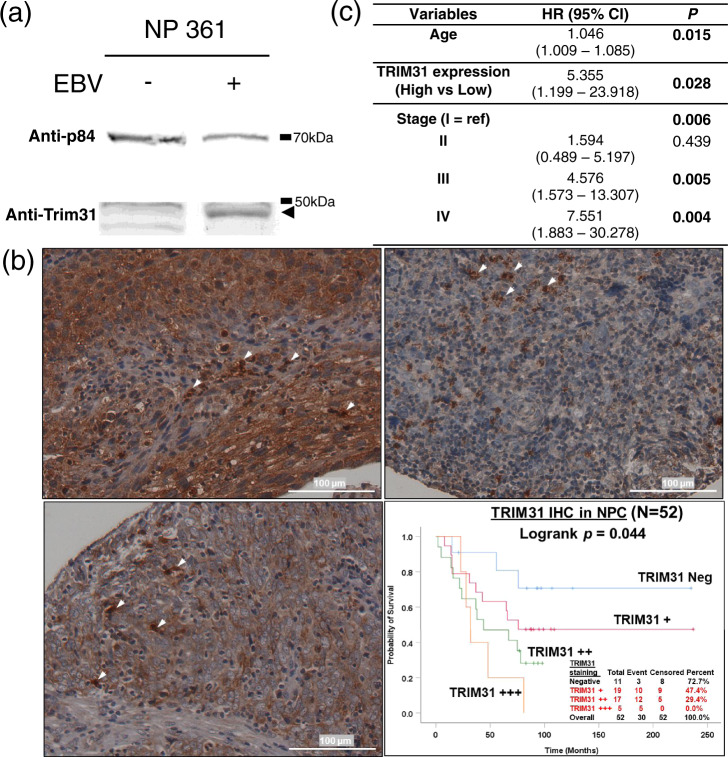
Correlation of TRIM31 expression and survival. **a** TRIM31 expression in EBV-infected normal immortalized nasopharyngeal epithelial cell line, NP361. Supplementary Fig. 7 showed the uncropped blots. **b** TRIM31 immunohistochemical staining clinically associated with poor survival in NPC. Representative images of NPC cells showing (upper right panel) high (+++) positive staining, (lower left panel) low (+) positive staining, and (upper left panel) negative TRIM31 expression in NPC tissue microarray after immunohistochemical staining. White arrowheads indicate the TRIM31-positive inflammatory cells. (Scale bar, 100 µm) For a subset of NPC with TRIM31-positive inflammatory cells, Kaplan–Meier survival analysis (lower right panel) indicates an association between higher TRIM31 expression in NPC and poorer survival compared to negative TRIM31 expression in NPC. **c** Multivariate COX regression analysis of age, TRIM31 expression, and stage with survival in NPC patients (*n* = 52).

NPC is closely associated with EBV infection and *TRIM31* deficiency attenuates innate antiviral responses to infections involves MAVS signaling^26^. Hence, we compared and detected induction of TRIM31 expression in EBV-infected normal immortalized nasopharyngeal epithelial cell cline NPC361 before and after EBV infection (Fig. 4a). Our previous methylome analysis reported *TRIM31* association with de novo methylation and reduced expression levels in NPC^27^. High TRIM31 expression associates with poor prognosis and drug resistance in various cancers including colorectal, pancreatic, and liver cancers^28–31^. Immunohistochemical staining of 133 NPC primary tissues showed significantly higher TRIM31 expression clinically associated with poorer survival by Kaplan–Meier analysis in a subset with positive TRIM31 expression in inflammatory cells (Fig. 4b, *P* = 0.044). The median overall survival (OS) of NPC patients from the tumor TRIM31 (+++) positive group (median OS 32 months) was significantly shorter compared to those from the tumor TRIM31 (++) group (median OS 44 months), TRIM (+) group (median OS 76 months) and TRIM negative group (median OS NR months). The age, gender, clinical stage, and survival information in this subset of 52 NPC patients are detailed in Supplementary Table 12. The median survival time of the NPC patients is 71 months and the range of survival is from 2 to 237 months. Univariate Cox regression indicates that age, stage, and tumor TRIM31 staining are significantly associated with survival, while gender and hospital are not associated with NPC survival. Multivariate Cox regression showed that age, clinical stage, and tumor TRIM31 status were independent prognostic indicators of NPC survival (Fig. 4c). Further mechanistic studies are needed for the functional role of TRIM31 in NPC pathogenesis.

### Novel rare variant association with NPC

Our study identifies a novel significant protective effect for two rare HLA alleles, HLA-B*07:05 (>66.7-fold) and HLA-C*15:05 (>58.8-fold), and four previously reported rare alleles (HLA-A*31:01, HLA-B*13:01, HLA-C*03:04, and HLA-B*39:01) (Supplementary Table 3 and Fig. 4). Both HLA-B*07:05 and HLA-C*15:05 alleles are absent from the 2752 NPC cases, but only present in ~1% control population (*n* = 2937) (HLA-B*07:05, OR < 0.015, *P* = 5.83 × 10^−21^ and HLA-C*15:05, OR < 0.017, *P* = 5.19 ×10 ^−18)^. Rare variant association analysis included 103,725 SNPs and *HLA-A*/*B*/*C* alleles and amino acids with MAF < 1%. Twenty-four rare SNPs, two HLA alleles (HLA-A*31:01 and HLA-B*07:05), and HLA-B_aa-156R_ (*P* = 6.28 × 10^−26^, OR = 0.021) confer strong protective effects for NPC (Supplementary Table 13) and reached the genome-wide significance. If a less stringent *P*-value cut-off (<1e−06) is set, 5/644 significantly associated rare SNPs are associated with increased NPC risk. Most individuals carry these rare SNPs segregating with HLA-A*31:01 and HLA-B*07:05 alleles. The two most significant rare variants, HLA-B_aa-156R_ and rs2596540 (*P* = 1.03 × 10^−24^, OR = 0.032), a putative functional SNP located at the 5′-UTR of *MICA*, are in tight linkage disequilibrium. Functional annotation of these rare variants prioritizes rs77803816 at *HCP5* in a region with high H3K27Ac marks predicting regulatory elements and involvement in transcription factor binding. Functional evidence suggests rs77803816 A-allele exhibits higher regulatory enhancer effects (Supplementary Fig. 5).

## Discussion

Using MHC region two-phase deep sequencing of the largest cohort reported to-date, we identified eight independent common loci associated with NPC as compared to only three previously reported independent loci by NPC GWAS studies (Supplementary Table 10)^7,10,11,14,32^. The majority of these independent signals (7/8) at the MHC region confer protection. The protective signals for NPC are not generally observed for other cancers. Further MHC sequencing investigation in other cancer types in the Hong Kong population, together with ESCC cases from Henan in Mainland China, did not detect similar results (Supplementary Table 14). Similar target-capture studies in other cancers associated with the MHC region, such as lung cancer and the viral-associated cancers, liver and cervical cancers, may help to address whether these seven protective signals identified or other SNPs in strong LD with these are specific for NPC. In summary, this current study identified two independent opposing signals (susceptibility rs9391681 and protective HLA-A_aa-Q62_) related to the *HLA-A* locus, three signals related to *HLA-B* locus, one near *HLA-DQA1,* and two novel non-HLA loci, *TRIM31* and *TRIM39* or *TRIM39-RPP21*. Three of the five independent signals at *HLA-A/B* are previously unreported SNPs rs9391681, rs2523589, and rs9265975 in tight linkage with potentially functional SNPs residing on regulatory elements to possibly confer higher risk or protection for NPC by modulating gene expression.

Our data now provide novel clues linking reduction of NPC risk with non-HLA genes near *TRIM31* and *TRIM39* or *TRIM39-RPP21* loci independent of the HLA Class I and II alleles and HLA-A/B/C amino acid variants. This is of importance since the TRIM proteins are a big family of 80 distinct ubiquitin E3 ligases involved in many cellular processes that have central roles in antiviral host defenses^33^. TRIM proteins regulate innate immune sensing, direct restriction of viral infection, and autophagy-mediated antiviral immune responses in mammalian cells^33^. The read-through transcript *TRIM39-RPP21* is known to mediate interferon response, a major host immune cytokine response against viral infection^34^. The intimate interaction between the host immune system and EBV infection at the nasopharyngeal epithelium appears to be essential for NPC development. Several key EBV proteins such as BDLF3, EBNA-1, EBNA-3C, LMP-1, affect the ubiquitin–proteasomal system with E3 ligases impacting host immune evasion, apoptosis, and cell cycle deregulation through degradation of substrates such as MHC I, MHC II, p53, Rb, p21, and NF-κB pathways^35^. Our findings now provide evidence and unique insight on the genetic linkage of host immunity and the development of viral latency with its consequential impact on NPC development risk. This is the first report linking *TRIM31* to reduced NPC risk and poor survival. Recent studies suggest that TRIM31 regulates MAVS aggregation upon viral infection to activate the innate immune response as the first-line defense against invading pathogens^26^. Mechanistically, TRIM31 regulates various molecular pathways including NLRP3, MAVS, NFκB, and p53 signaling, through its ubiquitin ligase activity to induce lysine-linked polyubiquitination of protein substrates affecting key cancer-related biological processes and regulating inflammation, antiviral infection, innate immunity, drug resistance, and metastasis^26,28,29,36^. These findings support the clinical relevance of TRIM31, as a potential independent poor prognostic marker in NPC due to altered NFkB signaling critical for NPC development^37^. Its role in promoting tumor aggressiveness was previously reported in various solid tumors including pancreatic, colorectal, and liver cancers^28,29,31^. We now show up-regulation of TRIM31 in a near-normal immortalized non-tumorigenic nasopharyngeal epithelial cell line infected with EBV. We speculate that TRIM31 may be involved in viral stimulation of innate immune responses, which is an early event preceding cancer initiation to maintain tissue homeostasis. These seemingly contradictory observations may be related to the different timing and versatile roles that TRIM31 has in cancer initiation, tumor progression, and the development of drug resistance^38^. Targeting TRIM31 signaling in pancreatic cancer was suggested to have therapeutic potential, as overexpression of TRIM31 activated NFkB resulting in gemcitabine resistance^28^. Our findings suggest the potential therapeutic option for targeting TRIM31 signaling. TRIM39 is a RING domain-containing E3 ubiquitin ligase that regulates the p53, APC/C, and NFκB pathways controlling apoptosis, cell proliferation, and inflammatory signaling by ubiquitylation^21,22,39,40^. In NPC, TRIM39 functionally affects cell proliferation, epithelial–mesenchymal transition, and metastasis and clinically correlates significantly with tumor size, stage, and metastasis^41^. The *TRIM39* or *TRIM39-RPP21* locus is associated with Behcet’s disease, a chronic inflammatory autoimmune disease^42^. GWAS reports indicate a linkage to cutaneous lupus erythematosus^23^ and behavioral risk tolerance^43^ and identified rs2517664 near *TRIM31* as one of the independent loci associated with general cognitive function and immune regulatory loci for mouth ulcers^44,45^. Further in-depth functional characterization of the genetic causal factors linked with *TRIM31, TRIM39*, and *TRIM39-RPP21* loci in NPC pathogenesis is warranted, but this is beyond the scope of the present study. Our findings provide new insights for NPC pathogenesis regarding TRIM-mediated anti-EBV host innate immune defense^35,46^.

Previous NPC GWAS suggest the only independent disease-driven *HLA-A* signal coming from rs2860580 in tight linkage disequilibrium with the HLA-A*11:01^7,8,10,11^. The protective role of HLA-A*11:01 allele is supported by the efficient presentation of EBNA4 antigens to elicit an immune response by the cytotoxic T-lymphocytes upon EBV infection^47^. Our fine-mapping study comprehensively demonstrates two disease-driven independent *HLA-A* signals with opposing effects reaching genome-wide significance, the Asian-specific rs9391681 and the HLA-A_aa-Q62_, which is carried not only by HLA-A*11:01, but 15 other *HLA-A* alleles (Supplementary Table 6). Our proxy variant analysis and functional genomics annotation prioritize four putative functional variants, rs143982339, rs9380181, rs9348841, and rs9380182, also tightly linked with rs9391681, suggesting these candidate functional variants may affect the binding of multiple transcription factors; further investigation of their regulatory mechanisms is of interest.

The highly diversified *HLA-B* locus in the Han Chinese population was previously suggested to have critical contribution in environmental adaptation^25^. Our study cohort detected 190 different *HLA-A*/*B*/*C* alleles with the greatest diversity observed for the 98 *HLA-B* alleles. The frequency distributions of *HLA-A/B/C* alleles in Hong Kong and Mainland China populations are shown (Supplementary Fig. 4). Near the *HLA-B* regions, there are multiple independent protective signals that reached genome-wide significance; two SNPs (rs2523589 and rs9265975) with moderate effect are located upstream and downstream of *HLA-B* and there is a strong protective effect of HLA-B*55:02 and HLA-B*07:05 alleles (Table 1 and Supplementary Table 3). GWAS identified rs2523589 as one of the immune regulatory loci associated with mouth ulcers and medication use in the UK-biobank^44,48^. Our data provide novel mechanistic insights for the involvement of putative functional regulatory variants affecting transcription factor binding and transcription of other non-HLA genes, additional to the regulation of its closest gene *HLA-B* expression with NPC risk. Further functional studies are required to address the causal factors in the same proxies associated with rs2523589 and rs9265975. The HLA-B amino acids associated with NPC across studies are inconsistent due to the disease signals being in tight linkage disequilibrium with *HLA-B* alleles. The great diversity of *HLA-B* alleles may provide an explanation for the discrepancy of different spectra of HLA-B amino acid polymorphisms between our Hong Kong and the Guangdong and Guangxi study^10^. Our findings further suggest multiple common and rare independent variants near *HLA-B*, rather than only a single independent signal rs2894207 reported by earlier GWAS^7^. Our study provides additional evidence and suggests an epigenetic regulatory function of the rare intronic SNPs rs77803816 of *HCP5* and rs2596540 of *MICA* (Supplementary Table 13). We cannot distinguish the protective effects between rs77803816 and HLA-B*07:05 (*D*′ = 0.46). Further studies are required to elucidate if the novel association of HLA-B*07:05 allele is attributed to HLA-B_aa-R156_ or other rare functional variants such as rs2596540 at *MICA* and rs77803816 in *HCP5. HCP5* is a regulatory lncRNA 100 kb centromeric from *HLA-B* involved in adaptive and innate immune responses, multiple autoimmune and viral-associated diseases such as HIV-1-associated AIDS and HCV-associated hepatocellular carcinoma^49–53^. MICA is a stress-induced ligand for NKG2D (Natural-killer group 2, member D) receptor recognized by NK cells and T cells.

SKAT gene association findings reinforce our observations on novel loci near *TRIM31* and *TRIM39* and the *HLA-B* identified from single SNP associations (Table 1 and Supplementary Table 11). The gene-level evidence (*MICA P* = 2.44 × 10^−26^, Supplementary Table 13) supports a possible role of the rare putative functional SNP, rs2596540 located in the 5′ UTR of *MICA*. Earlier NPC *MICA* association studies were limited by a small sample size; the reported signal is not an independent signal in our current study^13,15,54^.

Our current study and earlier GWAS consistently detected only one independent signal near the *HLA-DQA1* of the MHC class II region and suggested dysregulated antigen presentation. However, the detected signal at rs9461780 near *HLA-DQA1* was not in the same proxy with rs28421666^7^, while others reported *HLA-DQB1* and *HLA-DRB1* loci^4,13,17,55,56^. The discrepancy may be partially due to studies of different populations. The underlying causal factor in the MHC class II region for NPC remains unclear.

NPC is rare in most populations worldwide. The absence of the rs9391681 risk allele and the greater abundance of the multiple protective alleles of rs2523589, rs9265975, rs2517644, and rs9260033 tagging HLA-A_aa-Q62_ in other ethnic populations including African, Caucasian, Ashkenazi Jews and a general Chinese cohort in contrast to NPC endemic regions is consistent with host genetic factors being critical determinants in NPC. The most common susceptibility H1 haplotype in Hong Kong Chinese suggests individuals lacking all seven protective alleles have a higher NPC risk (OR1 = 1.56). NPC risk (OR2 = 1.77) further increased for individuals carrying the H2 haplotype of the susceptibility rs9391681 C-allele. The control population with lower NPC risk carry moderate and strong protective haplotypes with different combinations of protective alleles including HLA-A_aa-Q62_, SNPs near *HLA-B*, *HLA-DQA1*, B*55:02, and novel loci at *TRIM31* and *TRIM39* in the non-classical HLA genes (Table 2). Our data implicate host genetic factors act as important components interacting with environmental and EBV exposure, affecting the efficiency of EBV antigen presentation to the host immune system and inhibition of innate immunity. The haplotype information and genetic heterogeneity of MHC NPC susceptibility loci across East Asian and other populations provide clues to partially explain the fact that despite ubiquitous EBV infection, worldwide NPC incidence is low, while it is especially high amongst East Asians and Southern Chinese. Further studies in NPC endemic regions from different ethnicities are warranted to validate our observation in Southern Chinese NPC patients.

In conclusion, this study of the MHC region identified seven independent signals within the MHC class I region and one within the MHC class II region for NPC risk. Findings enhance our understanding of the genetic basis for NPC predisposition in individuals carrying the only two susceptibility haplotypes lacking all the protective alleles involved in both HLA and non-HLA genes. The timing for EBV exposure and infection, host responses to the ubiquitous tumor virus infections, and the development of viral latency are complex contributors to the development of NPC in high-risk populations. The detection of an association of novel *TRIM31, TRIM39*, or *TRIM39-RPP21* signals with NPC highlight the expected importance of host innate immune responses impacting NPC genetic susceptibility.

## Methods

### Study participants of Hong Kong cohorts

Two independent cohorts of 5698 unrelated individuals, including 3056 participants in the discovery phase (1438 NPC cases and 1618 matched healthy controls) and 2642 participants for the validation phase (1321 NPC cases and 1321 matched healthy controls), were utilized in this study (Supplementary Fig. 1a). The demographic characteristics of patients and controls are summarized in Supplementary Table 1. The overall design of this study is shown in Supplementary Fig. 1. The NPC cases and controls were collected by the Tissue Bank established under the Area of Excellence (AoE) program for the period of specimen collection from 2010 to 2017 and then randomly assigned as the discovery phase and validation phase of this study.

All patients and healthy individuals were matched Han Chinese from Hong Kong (Supplementary Fig. 6). NPC patients were recruited from five Hong Kong public hospitals including Queen Mary Hospital (QMH), Queen Elizabeth Hospital (QEH), Tuen Mun Hospital (TMH), Pamela Youde Nethersole Eastern Hospital (PYNEH), and Princess Margaret Hospital (PMH) between 2010 and 2017. The 2937 individuals in the control population consisted of 1804 individuals from the Red Cross and 1133 hospital cancer-free individuals from QMH, QEH, TMH, PYNEH, and PMH. Blood samples were recruited from a total of 2752 NPC patients including 1918 newly diagnosed NPC cases prior to any therapy and 834 follow-up NPC patients. All NPC patients were confirmed by histopathology and enrolled with routine staging procedures according to the American Joint Committee on Cancer (AJCC) TNM system, physical examination, and imaging tests. The study was approved by the Institutional Review Board (IRB) of the University of Hong Kong.

### Study participants of Guangdong cohort

Regional genotype data for 1583 NPC cases and 3040 controls were retrieved from two NPC GWAS projects reported previously^7,57^. Briefly, all cases were patients diagnosed with primary NPC and recruited at Sun Yat-sen University Cancer Center (SYSUCC), Guangdong, China, from October 2005 to May 2012. The diagnosis was histopathologically confirmed by two pathologists at SYSUCC according to the World Health Organization (WHO) classification. Healthy controls were recruited from physical examination centers of several large comprehensive hospitals in local communities in Guangdong. All the participants were self-reported as Southern Han Chinese ancestry. All the controls were self-reported with no history of malignancy at the time of enrollment. All the study subjects signed informed consent and studies were approved by institutional ethical committees of Sun Yat-sen University.

### MHC-target sequencing, variant calling, and HLA typing

The libraries for MHC-target sequencing were prepared and sequenced in the Illumina HiSeq platform according to manufacturer instructions^5^. Briefly, the pre-capture sequencing libraries were prepared with 100–200 ng genomic DNA after fragmentation to 300–400 bp by Covaris according to the KAPA HyperPrep Kits^58^ and hybridized to the SeqCap EZ Choice Enrichment Kit (Roche). Each capture included 24 indexed libraries of blood DNAs. The capture kits were designed using NimbleDesign (https://design.nimblegen.com/nimbledesign, Roche Sequencing Solutions, Inc) to capture the whole MHC region (chr6: 28,510,120–33,480,577, hg38) and other non-MHC regions. The quality of libraries was evaluated by bioanalyzer analysis (Agilent Technologies). The quantity of libraries was assessed by qubit measurement and Q-PCR quantification. The post-capture libraries were sequenced using 150 bp paired-end reads on the Illumina HiSeq platform. Sequencing reads were aligned to the human reference genome hg38 (hg38 + Alt + decay) using Bwa.kit (https://github.com/lh3/bwa/tree/master/bwakit)^59^ (Supplementary Fig. S1b). The PCR duplicates were removed from the aligned file (bam) using SAMtools (version 1.3.1)^60^. Picard (version 2.2.1) was used to calculate the HsMetrics (including the coverage of the targeted region, off-target ratio) from bam files to assess the sample quality. The germline variants (SNP/InDels) were called using mpileup2snp from VarScan (version 2.3.8)^61^ at base resolution in the target region (pileup2cns) with parameters (minimum coverage: 8; minimum supported alternative reads: 2; minimum alternative allele frequency: 0.2; minimum average quality: 15; *P*-threshold:0.01; minimum mapping quality 15). The function of the SNPs/InDels were annotated using ANNOVAR^62^ and functional trackers from UCSC Genome Browser^63^.

At the sample level, we filtered out samples with insufficient sequencing coverage (<15×), a high off-target ratio, and a high missing ratio (>10%). At the variant level, we filtered variants with a high missing ratio (>10%). The variants that violated the Hardy–Weinberg equilibrium in the control samples were also filtered out (Hardy–Weinberg equilibrium *P* < 1e−06). The InDels with length larger than four base pairs or multiple InDels in the same location are filtered out. The identity-by-descent test was performed using PLINK^64^ for variants in the MHC region to remove some potentially related samples (PI_HAT > 0.3). The principal components analysis and multi-dimensional scaling were used to check whether there were any population stratification between cases and controls. The alignments of top variants and significantly associated InDels were manually checked in the Integrative Genomics Viewer^65^ for some randomly selected samples.

The classic HLA class I alleles were typed at the 4-digit resolution from cleaned FASTQ files using OptiType^66^. The protein sequences of each allele were generated according to the IMGT/HLA database (version 3.31.0)^67^ based on the typed HLA alleles. The consensus sequence of each HLA gene (*HLA-A*, *HLA-B*, *HLA-C*) was generated from multiple sequence alignment using Clustal Omega^68^.

The final variant sets (SNPs/InDels/HLA class I alleles/ HLA class I amino acid polymorphism) were imputed and phased using Beagle (version5.0)^69^. Linkage disequilibrium statistics (*D*′ and *r*^2^) were calculated by PLINK.

### Association analysis

The common variants MAF ≥ 1% were analyzed by logistic regression model adjusted for sex and age to test the association between NPC and controls using PLINK^64^. The forward stepwise conditional logistic regression analysis was used to identify the independent signals. Pairwise conditional logistic regression was performed between all significantly associated variants to check their independence. For the rare variants (MAF < 1%), the chi-square test or Fishers’ exact test were used to test the association between NPC and controls. The Fishers’ exact test was used when any of the expected values of the 2 × 2 contingency table was below 5. The gene-level burden test was performed with all the variants using the SNP-set kernel association test (SKAT) from Rvtests^70^ based on the NCBI RefSeq annotation and a set of parameters with a higher weight on rare variants (nPerm = 10,000, alpha = 0.001, beta1 = 1, beta2 = 25).

A classical genome-wide significance cut-off (5 × 10^−8^) was used as the threshold for significance for both common and rare variants. HLA alleles passing the multiple test adjustment with Bonferroni correction (*P* < 2.63 × 10^−4^) were considered significant.

### Validation of TRIM31 association in Guangdong NPC cohort

The original data coordinates obtained from GWAS NPC^7^, including 3040 controls and 1583 NPC patients, are converted via liftOver to hg19 coordinates. Genome-wide imputation was done through SHAPEIT + IMPUTE2 (1000 Genomes as reference) procedure. Imputed results were converted to PLINK format using gtools (quality threshold > 0.9). We performed imputation for un-genotyped SNPs in the MHC region (27–35 Mb on chromosome 6) using SNP2HLA (v1.0.3)^71^ with default parameters and pan-Asian reference panel^72^ (*n* = 530). Imputation quality controls were also applied and SNPs with INFO > 0.8, MAF > 0.01, and HWE > 1 × 10^−6^ (in controls) were used. SNPs showing extensive different missing rates between case–control groups (*P*-value < 1 × 10^−5^) were removed from downstream analysis. The association test was conducted using the logistic linear regression model.

### Analysis of public MHC-target sequencing data

A large data set sequencing the MHC region in more than 20,000 individual Chinese^25^ was analyzed using the same pipeline for the SNPs.

### Assessment of the accuracy of typing

The SNPs and HLA alleles were validated using Sanger sequencing. For SNPs, we randomly selected samples from candidate variants for SNP validations, as well as loci validation. The overall validation rate was 98.9% (86/87). For HLA alleles, primers covering exons 2 and 3 of the HLA-A gene were designed. The Sanger sequencing results were compared with the typed HLA-A alleles. The overall validation rate was 94.4% (17/18). The sequences of primers are listed in Supplementary Table 15. For HLA alleles, in addition to the direct sequencing, we further genotyped the HLA alleles using another HLA typing software Kourami^73^. The concordance rate between these two software was 98.5% (97.5% for *HLA-A*, 99.6% for *HLA-B*, and 98.5% for *HLA-C*).

### Immunohistochemical (IHC) staining and scoring of TRIM31 protein expression in NPC tissue microarray (TMA)

A TMA was constructed with formalin-fixed paraffin-embedded (FFPE) NPC specimens (*N* = 133) in duplicate. Specimens from patients ranging in age from 25 to 85 (mean age = 51.4 ± 11.72) and 78% males were included. There were 33 (24.8%) stage I, 42 (31.6%) stage II, 43 (32.3%) stage III, and 15 (11.3%) stage IV NPC patients. The median follow-up time was 78 months; 54.9% of patients died.

IHC analysis was performed to assess the expression of TRIM31 in the NPC TMA with anti-TRIM31 antibody (Proteintech, Cat#215711-AP) using the DAKO LSAB2 System-HRP (horseradish peroxidase) (DAKO) and the DAB substrate kit (ThermoFisher) according to the manufacturers’ instructions^37^. Antigen retrieval was performed by boiling slides in a microwave oven for 10–14 min in 10 mM citrate buffer (pH 6.0). Briefly, endogenous peroxidase block in the deparaffinized tissue sections was performed by incubation of 3% H_2_O_2_ for 30 minutes. Tissue sections were blocked in 10% goat serum diluted in PBS, followed by overnight incubation with the anti-TRIM31 antibody at 4 °C and a final concentration of 2 µg/ml. The slide was further incubated with biotinylated link antibody, streptavidin-HRP, substrate chromogen solution, DAB (3,3′-diaminobenzidine) and counterstained with hematoxylin for analysis using a bright field microscope. All staining was scored by a trained pathologist (LHT) and a score between 1 and 4 was given to tumor cells and stromal inflammatory cells (1 = negative staining, 2 = weakly positive, 3 = moderately positive, and 4 = strongly positive). Survival was determined from the date of diagnosis to the date of the last disease evaluation or until death/recurrence. Kaplan–Meier methods were used to analyze survival data. Statistical analyses were performed by SPSS version 26 and a *P* < 0.05 was considered statistically significant.

### Cloning and enhancer assay of rs77803816

Genomic sequences spanning ±250bp of SNP rs77803816 at *HCP5* were amplified by the primer set (F: TTCCACCTTTCCCAACCTGT; R: ACACCAAAGCAGGACCATTC) using corresponding patient samples as templates and were cloned into the Firefly luciferase-based lentiviral enhancer assay vector pLS-mP-Luc (Addgene #106253) at the SbfI site before the minimal promoter. The vectors were then expressed in an NPC43 cell line^74^ pre-labeled with ubiquitously-expressed Renilla luciferase. Luciferase activity was measured by adding d-luciferin potassium salt (BioVision, Inc., Milpitas, CA) and Enduren (Promega Corporation, Fitchburg, WI) for Firefly and Renilla luciferases, respectively, and quantified using an IVIS Spectrum In Vivo Imaging System (PerkinElmer, Waltham, MA). The transcriptional activity index was calculated as Firefly luciferase signal intensity normalized to Renilla luciferase signal intensity, followed by normalizing to the fragments of the wildtype (WT) G-allele in the same orientation of *HCP5* (Forward).

### Western blot analysis of TRIM31 protein expression in NP361 cells

Protein lysates of NP361 cells^75^ with and without EBV infection were run and separated by 10% SDS-PAGE. Proteins were transferred to PVDF membranes, which were incubated at 4 °C overnight with the primary antibodies against TRIM31 (1:1000) or p84 (1:1000) after blocking with 5% non-fat milk in TBST. The membranes were then incubated for 1 h with 1:5000 anti-mouse Alexa Fluor 800 or anti-rabbit Alexa Fluro 680. TRIM31 and p84 proteins were detected and visualized by GE Typhoon 5 Molecular Imager.

### Statistics and reproducibility

Statistical tests for TRIM31 TMA survival analysis included Kaplan–Meier survival curve, log-rank test, and multivariate Cox regression were generally considered significant with *P* < 0.05 (SPSS version 26). Measurements in enhancer assay of rs77803816 were taken from three independent biological replicates and Student’s two-tailed *t*-test analyzed using Prism, *P* < 0.05 was considered significant (GraphPad Software, San Diego, CA). Western blot images were representative from two biological replicates.

### Reporting summary

Further information on research design is available in the Nature Research Reporting Summary linked to this article.

## Supplementary information

Supplementary Information

Description of Additional Supplementary Files

Supplementary Data 1

Reporting Summary

## Data Availability

The DNA target sequencing data generated for this publication have been deposited in the European Genome-phenome Archive (EGA) under the accession number EGAS00001003995. The data reported in this study are available from corresponding authors upon request. The public Chinese MHC-target sequencing data can be accessed from the NCBI Sequence Read Archive (SRA) database using the accession ID SRA205317. Additional data related to this study can be found in Supplementary Data 1.
