# Supplementary material for: Nasopharyngeal carcinoma MHC region deep sequencing identifies HLA and novel non-HLA TRIM31 and TRIM39 loci
Source: Commun Biol. 2020 Dec 11;3:759. doi: 10.1038/s42003-020-01487-y (PMC7733486; doi:10.1038/s42003-020-01487-y)
Supplement: Supplementary file 1 — Supplementary Information [file 42003_2020_1487_MOESM1_ESM.pdf]

Supplementary Materials

Nasopharyngeal carcinoma MHC region deep sequencing identifies HLA and novel non-HLA *TRIM31* and *TRIM*39 loci

<u>Supplementary Figures 1-7</u>

Supplementary Figure 1: Study workflow and analysis pipeline

Supplementary Figure 2: Associations of amino acids at HLA-A, HLA-B, and HLA-C in Hong Kong NPC cohort.

Supplementary Figure 3: HLA-$A_{aa\text{-}C99}$ lies within the antigenic peptide binding groove and HLA-$A_{aa\text{-}Q62}$ lies in the MHC-TCR interaction locus.

Supplementary Figure 4: Frequency distribution of top 30 *HLA-A*, *HLA-B*, and *HLA-C* alleles in the Chinese from Hong Kong and China.

Supplementary Figure 5: Functional rare regulatory SNP rs77803816 at *HCP5*.

Supplementary Figure 6: Plots of first two principal components from PCA and dimensions from MDS of 5698 samples show the cases and controls are genetically homogenous.

Supplementary Figure 7: Uncropped blot of Figure 4a.

Supplementary Tables 1-15

Supplementary Table 1: Demographic details of study populations

Supplementary Table 2: Five independent common SNPs associated with NPC risk

Supplementary Table 3: Association of HLA alleles with NPC risk

Supplementary Table 4: Association of HLA allelic combinations with NPC risk

Supplementary Table 5: HLA haplotypes identified in Hong Kong cohort

Supplementary Table 6: Amino acids in HLA class I genes associated with NPC risk

Supplementary Table 7: Multivariate logistic regression analysis conditional for significant variants, HLA class I alleles and their amino acids

Supplementary Table 8: Eight independent signals for common variant association analysis after LD pruning

Supplementary Table 9: eQTL effects of rs2523589 and rs9265975 from GTEx portal.

Supplementary Table 10: Comparison with previous variants reported by GWAS

Supplementary Table 11: Top 15 genes associated with NPC by SKAT gene association analysis.

Supplementary Table 12: Clinical characteristics of NPC cases with positive TRIM31 expression in inflammatory cells

Supplementary Table 13: Association of rare variants with NPC risk

Supplementary Table 14: Association of candidate variants from Henan ESCC and Hong Kong non-NPC cancer

Supplementary Table 15: Primers for Sanger sequencing validation

Supplementary Data 1

Supplementary Figure 1: Study workflow and analysis pipeline

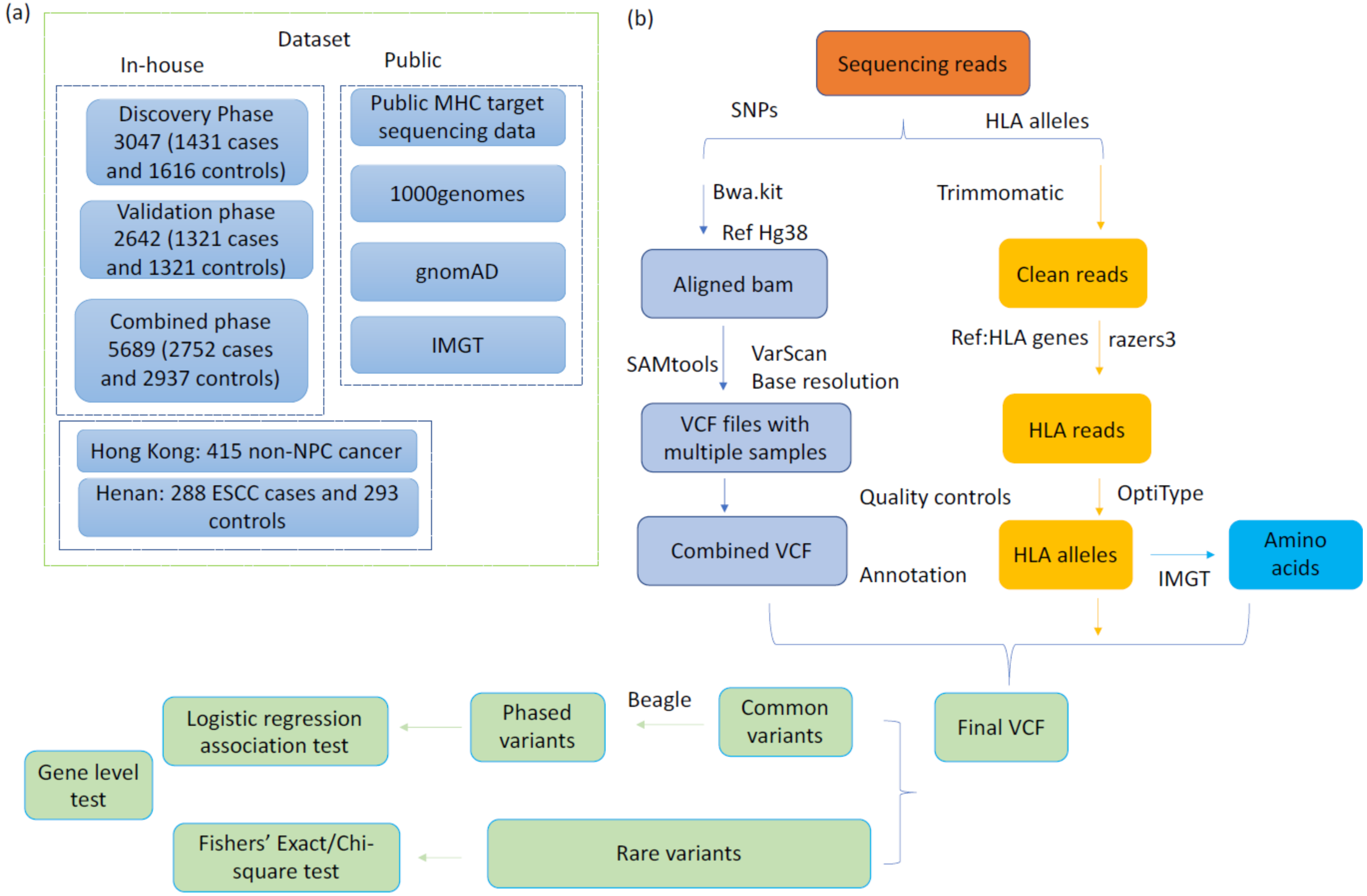

The total samples remaining for analysis after quality controls were 5689.

Supplementary Figure 2: Associations of amino acids at HLA-A, HLA-B, and HLA-C in Hong Kong NPC cohort.

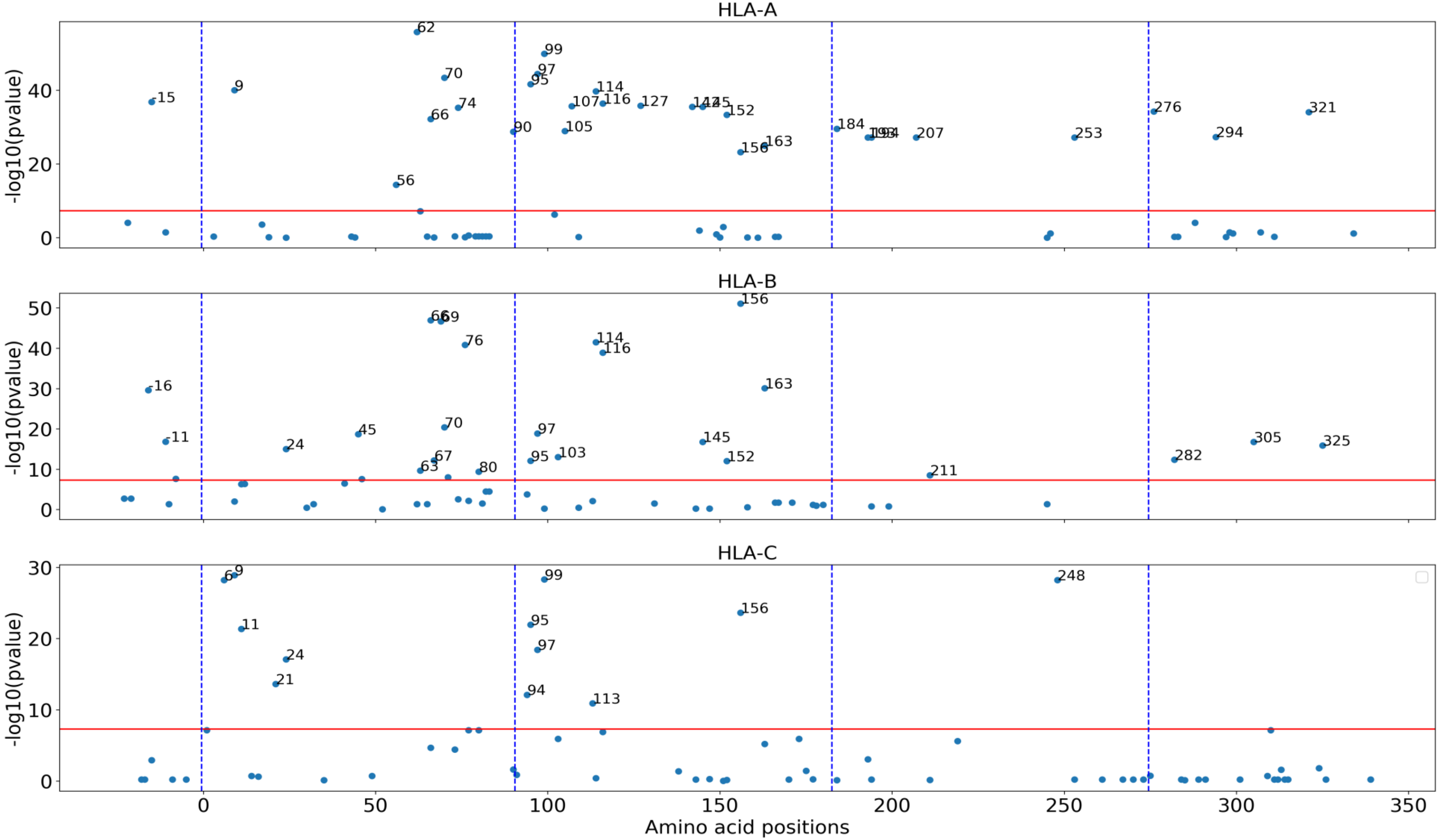

The red line presents the significance threshold *P*-value = 5e-08. The dashed blue line separates the exons 1-4 from left to right. The significant amino acids passing the significance threshold are annotated with their amino acid positions. For the positions with multiple amino acids, the omnibus *P*-value was used.

Supplementary Figure 3: HLA-A$_{aa\text{-}C99}$ lies within the antigenic peptide binding groove and HLA-A$_{aa\text{-}Q62}$ in the MHC-TCR interaction locus. Three-dimensional crystal structure of HLA-A*02:07 and HLA-A*11:01 with EBV epitopes, visualized using PyMOL. **(a)** HLA-A amino acid C^{99} is in high LD with HLA*02:07. HLA-A$_{aa\text{-}C99}$ lies within the antigenic peptide binding groove (PDB accession ID: 3OXS) **(b)** Amino acid Q^{62} is in high LD with HLA-A*11:01. HLA-A$_{aa\text{-}Q62}$ lies in the MHC-TCR interaction locus. (PDB accession ID: 5GSD).

a **b**

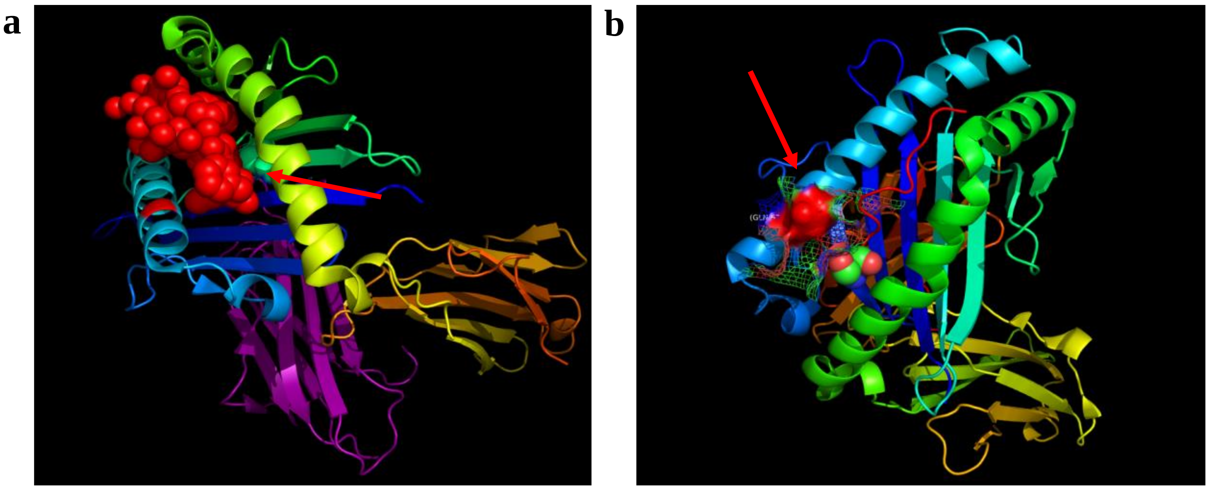

Supplementary Figure 4: Frequency distribution of top 30 *HLA-A*, *HLA-B*, and *HLA-C* alleles in the Chinese from Hong Kong and China. NPC case (N=2752) and control (N=2937) of Hong Kong Chinese population; and general Chinese cohort from psoriasis study labelled as Public (N=20206) [1]. Yellow arrows mark the eleven *HLA-A/B/C* alleles significantly associated with NPC in Hong Kong Chinese. The frequency of susceptible *HLA-A*02:07*, *HLA-B*46:01*, and *HLA-C*01:02* alleles were lowered while protective *HLA-A*31:01*, *HLA-B*55:02*, *HLA-B*39:01*, *HLA-B*07:05* and *HLA-C*15:05* alleles were higher in general Chinese from China compared to Hong Kong NPC population.

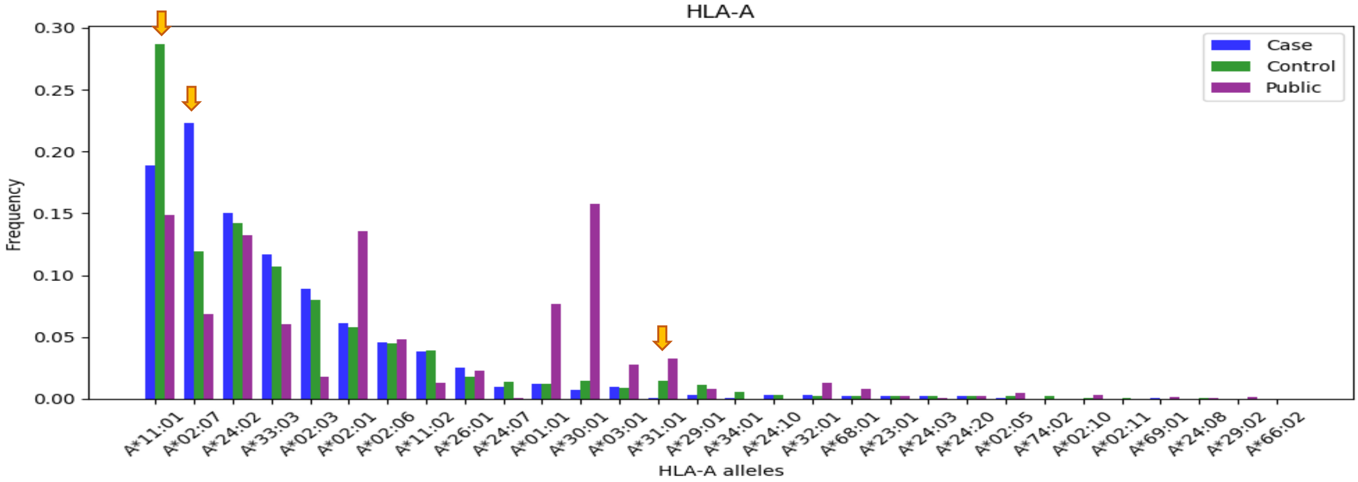

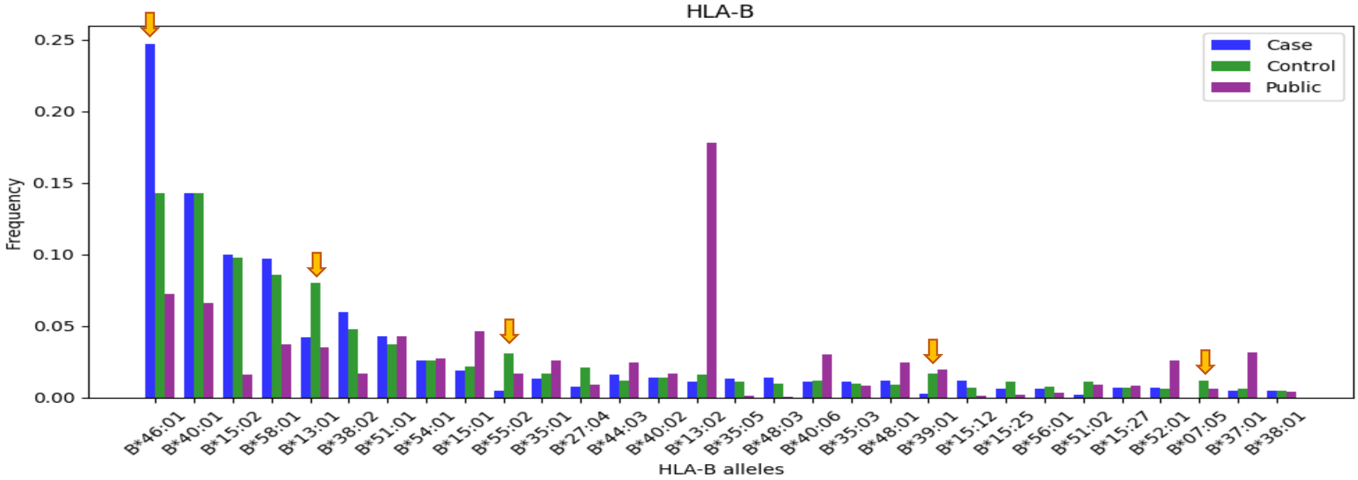

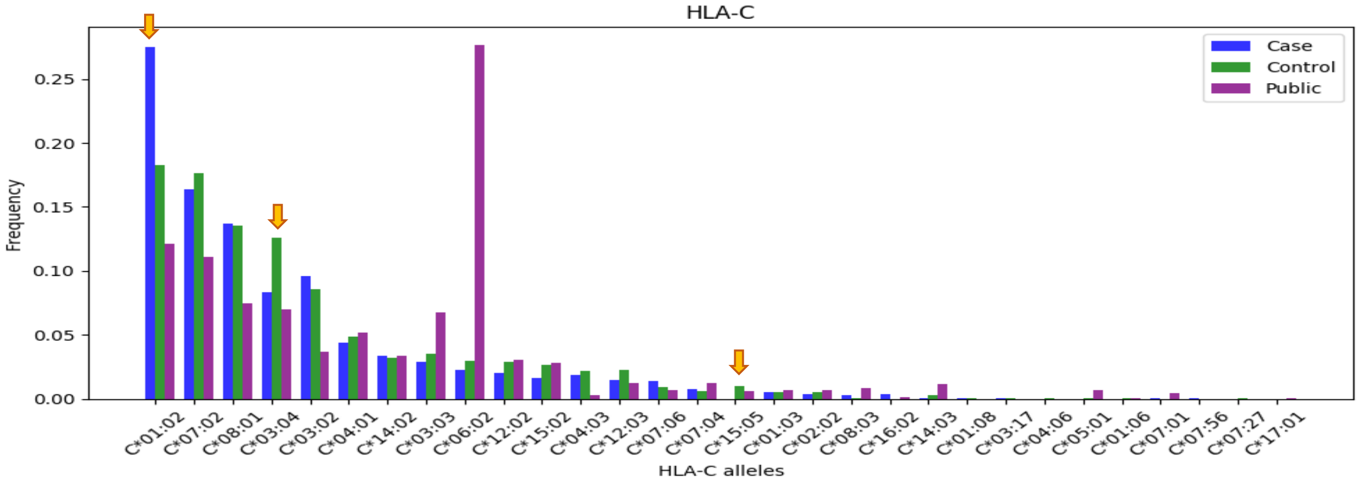

[1] Zhou F, Cao H, Zuo X et al. Deep sequencing of the MHC region in the Chinese population contributes to studies of complex disease. Nat Genet 2016; 48: 740-746.

Supplementary Figure 5: Functional rare regulatory SNP rs77803816 at *HCP5*. Schematic diagram for the location of *HCP5* and rs77803816. Five rare variants near *HCP5* along hg38 chr6:31,460,900-31,466,500. Three out of the five *HCP5* rare variants localized to the H3K27Ac mark, suggesting regulatory elements obtained from UCSC Genome Browser (http://genome.ucsc.edu). Rs77803816 is localized to multiple transcription factor binding sites (ORegAnno). The green box indicates the 500 bp DNA fragment localized to chr6:31463036-31463536 cloned into the luciferase reporter. Enhancer assay study of the rare *HCP5* variant (rs77803816) demonstrates significantly increased luciferase reporter activity of the minor A-allele compared to wildtype G-allele in both orientations of the cloned DNA fragment, upon transfecting NPC43 cells.

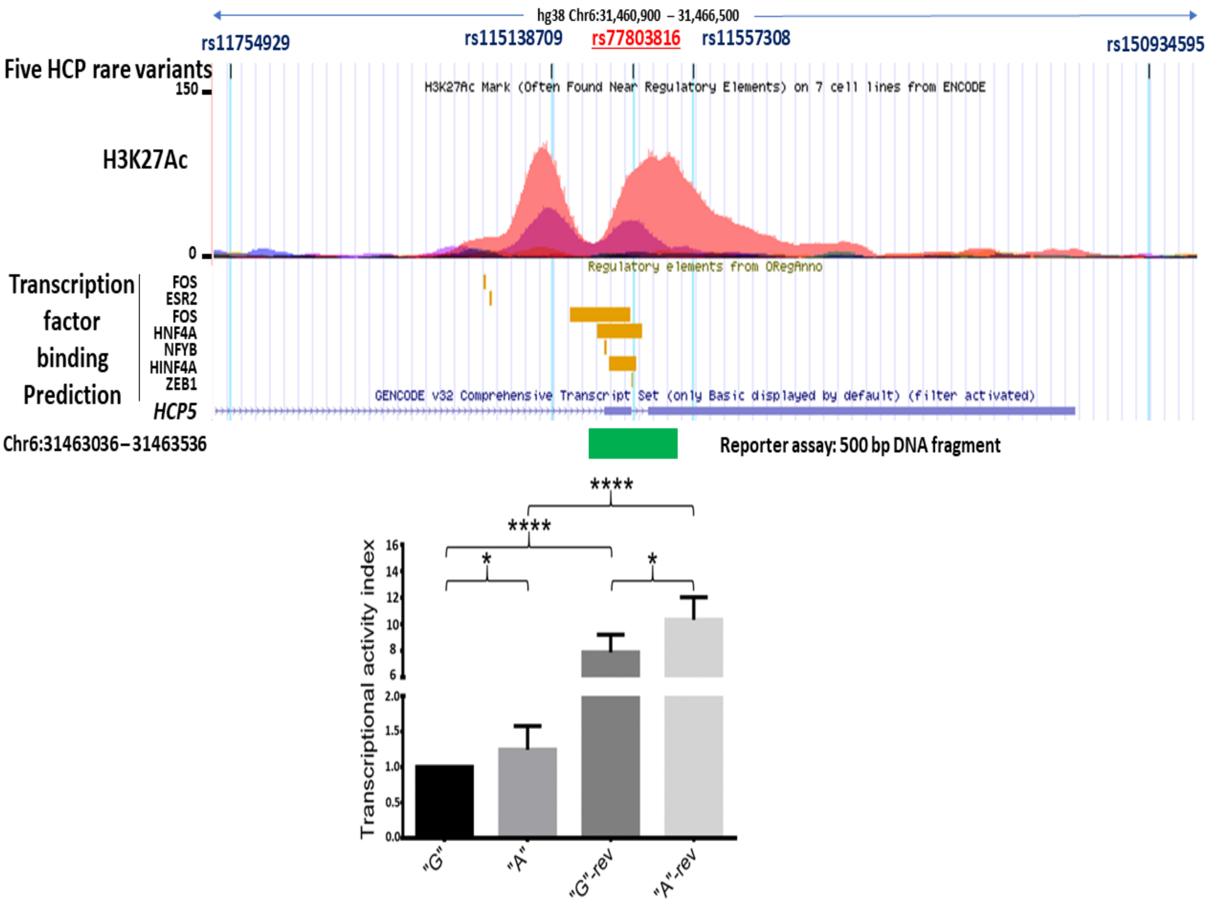

Figure legend:
*adjusted *p*-value <0.05
****adjusted *p*-value < 0.0001
Error bar represents 95% confidence interval, n=3.

Supplementary Figure 6: Plots of first two principal components from PCA and dimensions from MDS of 5689 samples show the cases and controls are genetically homogenous

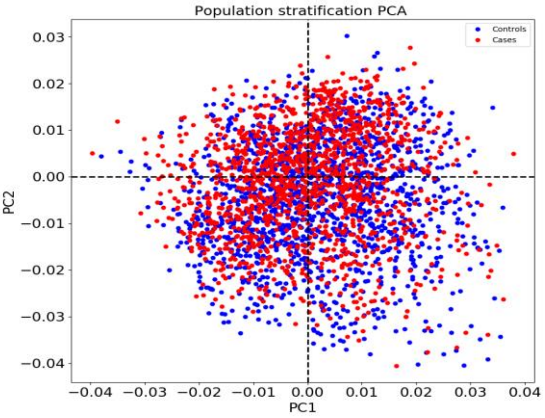

PCA non-MHC region

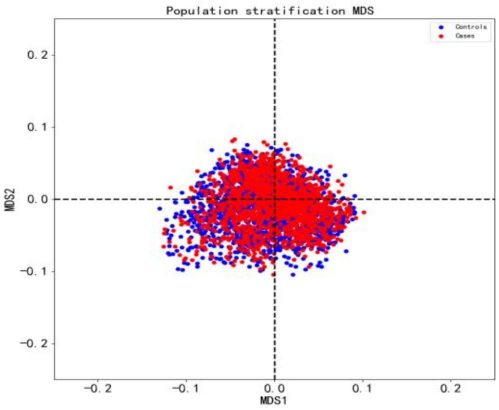

MDS non-MHC region

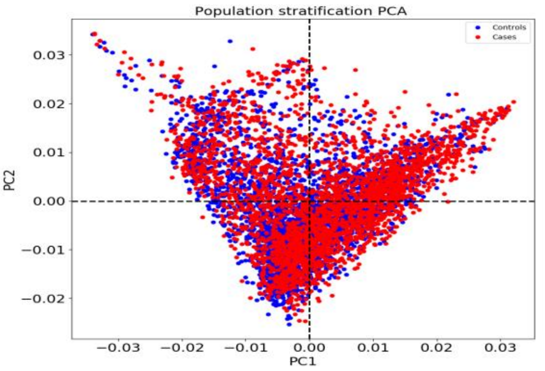

PCA MHC region

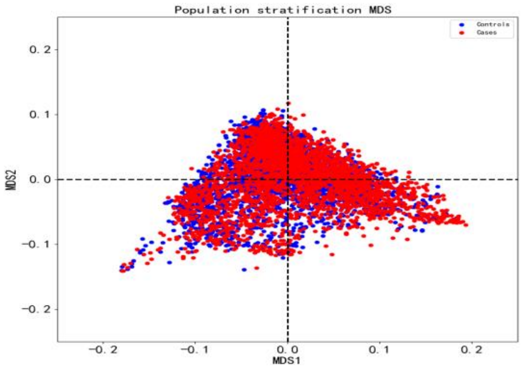

MDS MHC region

Supplementary Figure 7: Uncropped blot of Figure 4a.

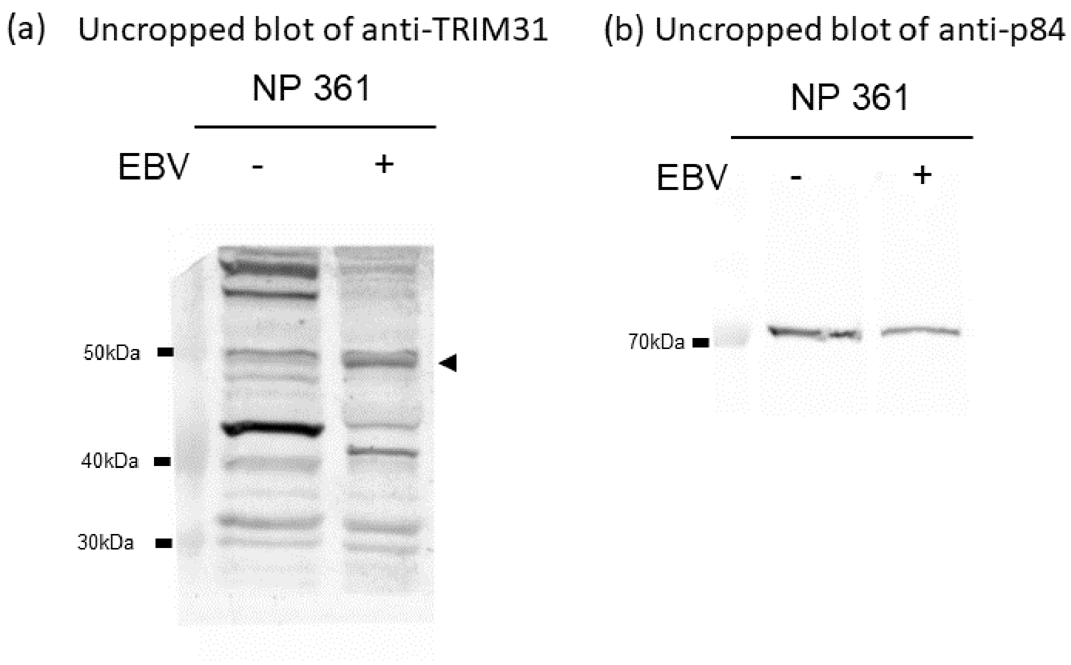

Supplementary Table 1: Demographic information of study populations

cohorts	Discovery (n = 3047)		Validation (n = 2642)		Combined (n = 5689)	
Variables	NPC cases (n = 1431)	Controls (n = 1616)	NPC cases (n = 1321)	Controls (n = 1321)	NPC cases (n = 2752)	Controls (n = 2937)
**Age (Mean ± S.D.)**	52.5 ± 12.7	51.8 ± 12.7	47.7±10.4	47.8±9.8	50.2 ± 11.9	50.0 ± 11.7
**Male (%)**	1102 (77.0)	1187 (73.5)	933 (70.6)	910 (68.9)	2035 (73.9)	2097 (71.4)
**Female (%)**	328 (23.0)	429 (26.5)	388 (29.4)	411 (31.1)	717 (26.1)	840 (28.6)

Supplementary Table 2: Five independent variants of common SNPs association analysis that reached genome-wide significance in two independent NPC cohorts.

SNP	Loci	Ref	Alt	Case MAF	Control MAF	Discovery		Validation		Combined		Conditional *P**
						OR (CI 95%)	*P*	OR (CI 95%)	*P*	OR (CI 95%)	*P*	
**rs9391681**	Intergenic *RPP21*HLA-E*	T	C	0.23	0.12	2.30 (2.20-2.64)	1.72E-31	1.90 (1.64-2.21)	3.68E-17	**2.11 (1.90-2.33)**	**1.50E-46**	**-**
**rs9260475**	6.5kb downstream *HLA-A*	T	C	0.26	0.38	0.55 (0.50-0.62)	9.10E-25	0.59 (0.53-0.67)	8.82E-18	**0.57 (0.53-0.62)**	**6.79E-41**	**1.42E-22**
**rs2523589**	2.4kb upstream *HLA-B*	T	G	0.24	0.35	0.56 (0.50-0.63)	1.05E-23	0.62 (0.55-0.70)	4.15E-14	**0.59 (0.54-0.64)**	**1.77E-36**	**1.79E-18**
**rs2517664**	intronic *TRIM31*	C	T	0.014	0.046	0.27 (0.19-0.39)	7.77E-13	0.31 (0.21-0.45)	1.66E-09	**0.29 (0.22-0.37)**	**6.38E-21**	**5.00E-12**
**rs3887381**	0.6kb downstream *HCG27*	C	T	0.22	0.30	0.62 (0.55-0.70)	2.62E-15	0.66 (0.58-0.75)	6.46E-11	**0.64 (0.59-0.69)**	**6.23E-25**	**6.37E-09**

MAF: minor allele frequency; OR: odds ratio; *P*: *P*-value
*Conditional *P* is calculated from the combined dataset.

Supplementary Table 3: HLA alleles associated with NPC risk

HLA alleles	Discovery				Validation				Combined				Remark#
	Case freq	Control freq	OR*	*P*	Case freq	Control freq	OR*	*P*	Case freq	Control freq	OR*	*P*	
A*02:07	0.23	0.12	2.20	1.09E-28	0.22	0.12	1.99	4.52E-15	0.22	0.12	2.10	1.11E-45	
B*46:01	0.26	0.13	2.14	1.62E-29	0.24	0.15	1.71	2.53E-14	0.25	0.14	1.93	1.64E-41	Common
A*11:01	0.18	0.29	0.55	1.00E-21	0.19	0.28	0.62	2.27E-13	0.29	0.29	0.58	3.03E-33	(p<5e-8)
C*01:02	0.29	0.18	1.84	5.30E-23	0.26	0.19	1.51	8.82E-10	0.28	0.18	1.68	3.22E-30	
B*55:02	0.0042	0.032	0.13	1.99E-11	0.0053	0.030	0.18	4.81E-09	0.0047	0.031	0.15	3.59E-19	
A*31:01	0.00035	0.017	0.02	6.15E-16	0.00077	0.013	0.029	1.89E-09	0.00055	0.015	0.036	6.26E-22	Rare
B*07:05	0	0.013	<0.027	5.94E-12	0	0.011	<0.033	8.52E-10	0	0.012	<0.015	5.83E-21	(p<5e-8)
B*13:01	0.042	0.084	0.49	1.32E-10	0.042	0.076	0.52	1.05E-07	0.042	0.080	0.50	6.86E-17	Common
C*03:04	0.079	0.13	0.57	1.39E-10	0.087	0.12	0.69	7.11E-05	0.083	0.13	0.63	1.22E-13	(p<2.6e-4)
C*15:05	0	0.011	<0.031	1.68E-10	0	0.009	<0.040	2.80E-08	0	0.01	<0.017	5.19E-18	Rare
B*39:01	0.004	0.017	0.27	9.73E-06	0.002	0.016	0.14	2.37E-07	0.003	0.017	0.21	8.12E-12	(p<2.6e-4)

Yellow highlight represents novel alleles associated with NPC observed in the current study.

Top seven alleles (5 common alleles and 2 rare alleles) pass genome-wide significance threshold ($P < 5 \times 10^{-8}$) in both discovery and validation phases; additional four alleles (rare alleles) pass the threshold of Bonferroni correction ($P < 2.6 \times 10^{-4}$) in both discovery and validation phases.

*Assume one allele is detected in the case for estimation of OR, which is not possible to be calculated when zero allele is observed.

#Common HLA alleles are defined as ≥1% in the whole population.

Supplementary Table 4: Combined HLA alleles with NPC risk

HLA alleles	Case P# freq	Control P# freq	OR**	*P* *
B*46:01, B*46:01	0.069	0.022	**3.30**	1.36E-17
A*11:01, A*11:01	0.033	0.085	0.36	5.83E-17
A*02:07, A*02:07	0.053	0.016	**3.50**	1.43E-14
A*02:07, A*24:02	0.069	0.029	2.46	4.50E-12
C*01:02, C*01:02	0.079	0.036	2.29	4.92E-12
A*02:07, A*33:03	0.052	0.024	2.19	7.60E-08
B*46:01, B*51:01	0.025	0.008	**3.06**	1.50E-06
A*24:02, A*31:01	0	0.008	<0.044	1.83E-06
A*11:01, A*29:01	0	0.008	<0.046	3.48E-06
B*55:02, B*58:01	0.001	0.009	0.081	3.64E-06
B*15:02, B*46:01	0.053	0.029	1.87	5.57E-06
C*01:02, C*08:01	0.08	0.051	1.60	1.71E-05
B*46:01, B*58:01	0.047	0.026	1.87	2.01E-05
B*40:01, B*46:01	0.065	0.04	1.66	3.14E-05
A*11:01, A*31:01	0	0.005	<0.067	3.32E-05

*Fisher's Exact test or Chi-square test. #A = absence of risk alleles; #P = presence of risk alleles. **Assume one individual is detected in the case for OR calculation.

Supplementary Table 5: HLA haplotypes associated with NPC risk

Haplotypes	Case freq	Control freq	OR**	*P**
*A*02:07-B*46:01-C*01:02*	0.0839	0.0362	**2.44**	7.65E-27
*A*11:01-B*13:01-C*03:04*	0.0067	0.0175	**0.38**	2.55E-07
*A*11:01-B*40:01-C*03:04*	0.0038	0.0107	**0.35**	2.65E-05
*A*02:07-B*51:01-C*14:02*	0.0045	0.0007	**6.68**	4.09E-05
*A*02:07-B*38:02-C*07:02*	0.0045	0.0007	**6.68**	4.09E-05
*A*11:01-B*40:01-C*07:02*	0.0100	0.0184	**0.54**	2.17E-04
*A*24:02-B*46:01-C*01:02*	0.0163	0.0088	**1.86**	4.57E-04
*A*29:01-B*07:05-C*15:05*	0.0000	0.0020	**<0.097**	5.18E-04
*A*02:07-B*40:01-C*01:02*	0.0080	0.0031	**2.62**	5.93E-04
*A*02:01-B*46:01-C*01:02*	0.0063	0.0020	**3.12**	5.94E-04
*A*11:01-B*55:02-C*08:01*	0.0000	0.0019	**<0.097**	1.03E-03
*A*02:07-B*15:02-C*08:01*	0.0076	0.0031	**2.50**	1.26E-03
*A*30:01-B*13:02-C*06:02*	0.0013	0.0048	**0.27**	1.37E-03
*A*11:01-B*39:01-C*07:02*	0.0004	0.0027	**0.13**	1.42E-03
*A*24:02-B*46:01-C*07:02*	0.0031	0.0007	**4.54**	3.50E-03
*A*11:01-B*07:05-C*15:05*	0.0000	0.0015	**<0.13**	4.03E-03
*A*24:02-B*13:01-C*03:04*	0.0025	0.0061	**0.41**	5.89E-03
*A*02:07-B*46:01-C*03:02*	0.0054	0.0022	**2.47**	7.97E-03
*A*11:01-B*55:02-C*01:02*	0.0005	0.0026	**0.21**	8.01E-03
*A*26:01-B*39:01-C*07:02*	0.0000	0.0014	**<0.13**	8.01E-03

* *P* calculated using Fisher's Exact test or Chi-square test. **Assume one haplotype is detected in the case for OR calculation.

Supplementary Table 6: Amino acids in HLA class I genes associated with NPC risk

	AA location	hg38	Ref	Minor allele	MAF	MAF case	MAF control	OR	OR low	OR high	*P*	Omnibus *P*	*P* -value (Tang *et al* , 2012, N=4055)	HLA alleles
HLA-A	-22	29942562	V	I	0.004	0.001	0.007	0.18	0.08	0.42	8.60E-05		2.58E-01	A*34:01, A*34:05
	-15	29942583	V	L	0.443	0.381	0.501	0.61	0.56	0.66	2.82E-37		1.91E-12	A*01:01, A*01:03, A*03:01, A*03:02, A*11:01, A*11:02, A*11:04, A*11:05, A*29:01, A*29:02, A*30:01, A*30:02, A*30:04, A*31:01, A*32:01, A*33:01, A*33:03, A*74:02
	-11	29942595	S	L	0.131	0.124	0.137	0.89	0.80	0.99	3.71E-02		3.52E-02	A*29:01, A*29:02, A*31:01, A*32:01, A*33:01, A*33:03, A*74:02
	3	29942764	H	Q	0.002	0.002	0.002	0.68	0.29	1.57	3.65E-01		3.03E-01	A*02:30,A*24:08,A*24:20
	7	29942776	Y	C	0.000	0.000	0.000	NA	NA	NA	NA		NA	A*24:25
	9	29942782	Other	Y	0.355	0.305	0.402	0.65	0.60	0.70	2.95E-27	9.70E-41	8.40E-11	A*02:05,A*02:06,A*02:10,A*02:44,A*02:51,A*11:01,A*11:02,A*11:04,A*11:05,A*11:13,A*11:20,A*26:01,A*26:09,A*34:01,A*34:05,A*66:01,A*66:02,A*68:01,A*69:01
		29942782	Other	F	0.340	0.398	0.285	1.66	1.54	1.80	4.34E-36		1.66E-06	A*01:01,A*01:03,A*02:01,A*02:03,A*02:07,A*02:11,A*02:12,A*02:13,A*02:20,A*02:30,A*02:42,A*02:74,A*03:01,A*03:02,A*32:01,A*74:02
		29942782	Other	S	0.178	0.176	0.180	0.98	0.89	1.08	6.66E-01		5.19E-01	A*23:01,A*24:02,A*24:03,A*24:07,A*24:08,A*24:10,A*24:20,A*24:25,A*24:27,A*24:63,A*30:01,A*30:02,A*30:04
		29942782	Other	T	0.127	0.121	0.133	0.89	0.80	1.00	4.28E-02		3.50E-03	A*29:01,A*29:02,A*31:01,A*33:01,A*33:03
	17	29942806	R	S	0.011	0.008	0.015	0.51	0.35	0.73	3.01E-04		1.13E-01	A*30:01,A*30:02,A*30:04
	19	29942812	E	K	0.039	0.039	0.040	0.97	0.81	1.16	7.48E-01		4.26E-01	A*11:02,A*24:63
	24	29942827	A	S	0.000	0.000	0.000	1.11	0.07	17.74	9.42E-01		NA	A*02:42
	43	29942884	Q	R	0.001	0.001	0.002	0.70	0.25	1.98	5.05E-01		1.70E-01	A*02:05
	44	29942887	R	K	0.012	0.011	0.012	0.96	0.68	1.34	8.08E-01		1.25E-01	A*01:01,A*01:03
	56	29942923	G	R	0.020	0.008	0.030	0.27	0.19	0.37	6.44E-15		1.22E-03	A*30:01,A*30:02,A*30:04,A*31:01
	**62**	29942941	Other	G	0.363	0.422	0.308	1.64	1.52	1.77	2.69E-35	**1.48E-56**	4.68E-14	A*02:01,A*02:03,A*02:05,A*02:06,A*02:07,A*02:10,A*02:11,A*02:12,A*02:13,A*02:20,A*02:30,A*02:42,A*02:44,A*02:51,A*02:74,A*24:08
		29942941	**Other**	**Q**	**0.324**	**0.261**	**0.383**	**0.56**	**0.52**	**0.61**	**2.29E-43**		1.17E-24	**A*01:01,A*01:03,A*03:01,A*03:02,A*11:01,A*11:02,A*11:04,A*11:05,A*11:13,A*11:20,A*30:01,A*30:02,A*30:04,A*31:01,A*32:01,A*74:02**
		29942941	Other	R	0.140	0.146	0.134	1.11	1.00	1.24	5.00E-02		1.21E-05	A*26:01,A*26:09,A*33:01,A*33:03,A*34:01,A*34:05,A*66:01,A*66:02,A*68:01,A*69:01
		29942941	Other	E	0.166	0.169	0.164	1.03	0.94	1.14	4.92E-01		7.60E-01	A*23:01,A*24:02,A*24:03,A*24:07,A*24:10,A*24:20,A*24:25,A*24:27,A*24:63
		29942941	Other	L	0.007	0.003	0.011	0.24	0.13	0.41	4.78E-07		1.03E-04	A*29:01,A*29:02
	63	29942944	E	Other	0.147	0.149	0.145	1.03	0.93	1.15	5.60E-01	6.66E-08	3.00E-04	A*01:01,A*01:03,A*02:01,A*02:03,A*02:05,A*02:06,A*02:07,A*02:10,A*02:11,A*02:12,A*02:13,A*02:20,A*02:30,A*02:42,A*02:44,A*02:51,A*02:74,A*03:01,A*03:02,A*11:01,A*11:02,A*11:04,A*11:05,A*11:13,A*11:20,A*23:01,A*24:02,A*24:03,A*24:07,A*24:08,A*24:10,A*24:20,A*24:25,A*24:27,A*24:63,A*30:01,A*30:02,A*30:04,A*31:01,A*32:01,A*74:02
		29942944	Other	N	0.140	0.146	0.134	1.11	1.00	1.24	5.00E-02		1.21E-05	A*26:01,A*26:09,A*33:01,A*33:03,A*34:01,A*34:05,A*66:01,A*66:02,A*68:01,A*69:01
		29942944	Other	Q	0.007	0.003	0.011	0.24	0.13	0.41	4.78E-07		1.03E-04	A*29:01,A*29:02
	65	29942950	R	G	0.166	0.169	0.164	1.03	0.94	1.14	4.92E-01		7.60E-01	A*23:01,A*24:02,A*24:03,A*24:07,A*24:10,A*24:20,A*24:25,A*24:27,A*24:63
	66	29942953	N	K	0.467	0.409	0.521	0.63	0.59	0.68	8.80E-33		5.74E-12	A*02:01,A*02:03,A*02:05,A*02:06,A*02:07,A*02:10,A*02:11,A*02:12,A*02:13,A*02:30,A*02:42,A*02:44,A*02:51,A*02:74,A*23:01,A*24:02,A*24:03,A*24:07,A*24:08,A*24:10,A*24:20,A*24:25,A*24:27,A*24:63,A*34:01,A*34:05
	67	29942956	V	M	0.012	0.011	0.012	0.96	0.68	1.34	8.08E-01		1.25E-01	A*01:01,A*01:03
	70	29942965	H	Q	0.325	0.261	0.385	0.56	0.51	0.61	6.27E-44		1.47E-21	A*03:01,A*03:02,A*11:01,A*11:02,A*11:04,A*11:05,A*11:13,A*11:20,A*24:07,A*29:01,A*29:02,A*30:01,A*34:01,A*34:05,A*66:01,A*66:02,A*68:01,A*69:01
	73	29942974	T	I	0.121	0.118	0.123	0.96	0.86	1.08	4.79E-01		2.44E-04	A*02:11,A*31:01,A*33:01,A*33:03
	74	29942977	D	H	0.362	0.422	0.307	1.65	1.52	1.78	5.99E-36		4.08E-14	A*02:01,A*02:03,A*02:05,A*02:06,A*02:07,A*02:10,A*02:12,A*02:13,A*02:20,A*02:30,A*02:42,A*02:44,A*02:51,A*02:74
	76	29942983	V	Other	0.210	0.211	0.208	1.02	0.93	1.12	6.73E-01	7.02E-01	3.66E-01	A*02:01,A*02:03,A*02:05,A*02:06,A*02:07,A*02:10,A*02:11,A*02:12,A*02:13,A*02:20,A*02:30,A*02:42,A*02:44,A*02:51,A*02:74,A*03:01,A*03:02,A*11:01,A*11:02,A*11:04,A*11:05,A*11:13,A*11:20,A*30:01,A*31:01,A*33:01,A*33:03,A*34:01,A*34:05,A*66:01,A*66:02,A*68:01,A*69:01,A*74:02
		29942983	Other	E	0.169	0.172	0.167	1.04	0.94	1.14	4.35E-01		7.72E-01	A*23:01,A*24:02,A*24:03,A*24:07,A*24:08,A*24:10,A*24:20,A*24:25,A*24:27,A*24:63,A*30:02,A*30:04,A*32:01
		29942983	Other	A	0.040	0.039	0.041	0.94	0.78	1.13	5.29E-01		1.17E-01	A*01:01,A*01:03,A*26:01,A*26:09,A*29:01,A*29:02
	77	29942986	D	Other	0.210	0.211	0.208	1.02	0.93	1.12	6.73E-01	2.32E-01	3.66E-01	A*02:01,A*02:03,A*02:05,A*02:06,A*02:07,A*02:10,A*02:11,A*02:12,A*02:13,A*02:20,A*02:30,A*02:42,A*02:44,A*02:51,A*02:74,A*03:01,A*03:02,A*11:01,A*11:02,A*11:04,A*11:05,A*11:13,A*11:20,A*30:01,A*31:01,A*33:01,A*33:03,A*34:01,A*34:05,A*66:01,A*66:02,A*68:01,A*69:01,A*74:02
		29942986	Other	N	0.207	0.208	0.206	1.01	0.92	1.11	8.23E-01		3.47E-01	A*01:01,A*01:03,A*23:01,A*24:02,A*24:03,A*24:07,A*24:08,A*24:10,A*24:20,A*24:25,A*24:27,A*24:63,A*26:01,A*26:09,A*29:01,A*29:02,A*30:02,A*30:04
		29942986	Other	S	0.002	0.003	0.002	1.90	0.88	4.12	1.05E-01		6.48E-01	A*32:01
	79	29942992	G	R	0.169	0.172	0.166	1.04	0.94	1.15	4.21E-01		7.90E-01	A*23:01,A*24:02,A*24:03,A*24:07,A*24:08,A*24:10,A*24:20,A*24:25,A*24:27,A*24:63,A*32:01
	80	29942995	T	I	0.169	0.172	0.166	1.04	0.94	1.15	4.21E-01		7.90E-01	A*23:01,A*24:02,A*24:03,A*24:07,A*24:08,A*24:10,A*24:20,A*24:25,A*24:27,A*24:63,A*32:01
	81	29942998	L	A	0.169	0.172	0.166	1.04	0.94	1.15	4.21E-01		7.90E-01	A*23:01,A*24:02,A*24:03,A*24:07,A*24:08,A*24:10,A*24:20,A*24:25,A*24:27,A*24:63,A*32:01
	82	29943001	R	L	0.169	0.172	0.166	1.04	0.94	1.15	4.21E-01		7.90E-01	A*23:01,A*24:02,A*24:03,A*24:07,A*24:08,A*24:10,A*24:20,A*24:25,A*24:27,A*24:63,A*32:01
	83	29943004	G	R	0.169	0.172	0.166	1.04	0.94	1.15	4.21E-01		7.90E-01	A*23:01,A*24:02,A*24:03,A*24:07,A*24:08,A*24:10,A*24:20,A*24:25,A*24:27,A*24:63,A*32:01
	90	29943025	A	D	0.316	0.265	0.364	0.62	0.57	0.68	1.42E-29		2.68E-16	A*01:01,A*01:03,A*11:01,A*11:02,A*11:04,A*11:05,A*11:13,A*11:20,A*26:01,A*26:09,A*34:01,A*34:05,A*66:01
	95	29943281	Other	I	0.470	0.409	0.528	0.62	0.57	0.66	2.93E-36	2.29E-42	4.02E-12	A*01:01,A*01:03,A*03:01,A*03:02,A*11:01,A*11:02,A*11:04,A*11:05,A*11:13,A*11:20,A*26:01,A*26:09,A*29:01,A*29:02,A*30:01,A*30:02,A*30:04,A*31:01,A*32:01,A*33:01,A*33:03,A*34:01,A*34:05,A*66:01,A*66:02,A*68:01,A*74:02

[illegible]	29943281	Other	V	0.362	0.422	0.306	1.65	1.53	1.79	2.46E-36	[illegible]	6.81E-14	A*02:01,A*02:03,A*02:06,A*02:07,A*02:10,A*02:11,A*02:12,A*02:13,A*02:20,A*02:30,A*02:42,A*02:44,A*02:51,A*02:74,A*69:01
	29943281	Other	L	0.168	0.170	0.166	1.03	0.93	1.13	5.98E-01		7.93E-01	A*02:05,A*23:01,A*24:02,A*24:03,A*24:07,A*24:08,A*24:10,A*24:20,A*24:25,A*24:27,A*24:63
97	29943287	Other	R	0.389	0.449	0.332	1.63	1.51	1.76	1.99E-35	3.61E-45	1.50E-14	A*02:01,A*02:03,A*02:05,A*02:06,A*02:07,A*02:10,A*02:11,A*02:12,A*02:13,A*02:20,A*02:30,A*02:42,A*02:44,A*02:51,A*02:74,A*26:01,A*26:09,A*34:01,A*34:05,A*66:01,A*66:02,A*69:01
	29943287	Other	M	0.300	0.295	0.304	0.96	0.89	1.04	2.98E-01		1.87E-01	A*01:03,A*23:01,A*24:02,A*24:03,A*24:07,A*24:08,A*24:10,A*24:20,A*24:25,A*24:27,A*24:63,A*29:01,A*29:02,A*31:01,A*32:01,A*33:01,A*33:03,A*68:01,A*74:02
	29943287	Other	I	0.312	0.257	0.363	0.60	0.55	0.65	1.49E-34		3.05E-20	A*01:01,A*03:01,A*03:02,A*11:01,A*11:02,A*11:04,A*11:05,A*11:13,A*11:20,A*30:01,A*30:02,A*30:04
**99**	29943293	Y	Other	0.337	0.392	0.285	1.61	1.49	1.75	1.63E-32	**1.26E-50**	3.19E-04	A*01:01,A*01:03,A*02:01,A*02:03,A*02:05,A*02:06,A*02:11,A*02:12,A*02:13,A*02:20,A*02:30,A*02:42,A*02:44,A*02:51,A*02:74,A*03:01,A*03:02,A*11:01,A*11:02,A*11:04,A*11:05,A*11:13,A*11:20,A*26:01,A*26:09,A*29:01,A*29:02,A*30:01,A*30:02,A*30:04,A*31:01,A*32:01,A*33:01,A*33:03,A*34:01,A*34:05,A*66:01,A*66:02,A*68:01,A*69:01,A*74:02
	29943293	Other	F	0.167	0.169	0.166	1.03	0.93	1.13	6.01E-01		7.11E-01	A*02:10,A*23:01,A*24:02,A*24:03,A*24:07,A*24:08,A*24:10,A*24:20,A*24:25,A*24:27,A*24:63
	**29943293**	**Other**	**C**	**0.169**	**0.223**	**0.119**	**2.10**	**1.89**	**2.33**	**1.11E-45**		4.58E-07	**A*02:07**
102	29943302	D	H	0.007	0.003	0.011	0.23	0.13	0.41	5.54E-07		1.79E-04	A*29:01
105	29943311	S	P	0.320	0.269	0.368	0.62	0.58	0.68	1.08E-29		1.26E-18	A*01:01,A*01:03,A*11:01,A*11:02,A*11:04,A*11:05,A*11:13,A*11:20,A*26:01,A*26:09,A*32:01,A*34:01,A*34:05,A*66:01,A*66:02,A*74:02
107	29943317	G	W	0.362	0.422	0.306	1.65	1.53	1.79	2.22E-36		4.68E-14	A*02:01,A*02:03,A*02:05,A*02:06,A*02:07,A*02:11,A*02:12,A*02:13,A*02:20,A*02:30,A*02:42,A*02:44,A*02:51,A*02:74,A*69:01
109	29943323	F	L	0.004	0.003	0.004	0.87	0.47	1.60	6.59E-01		7.63E-05	A*32:01,A*74:02
114	29943338	H	Other	0.470	0.409	0.528	0.62	0.57	0.66	2.93E-36	1.84E-40	5.06E-12	A*02:01,A*02:03,A*02:05,A*02:06,A*02:07,A*02:10,A*02:11,A*02:12,A*02:13,A*02:20,A*02:30,A*02:42,A*02:44,A*02:51,A*02:74,A*23:01,A*24:02,A*24:03,A*24:07,A*24:08,A*24:10,A*24:20,A*24:25,A*24:27,A*24:63,A*69:01
	29943338	Other	R	0.309	0.253	0.362	0.59	0.54	0.64	8.61E-36		5.74E-22	A*01:01,A*01:03,A*03:01,A*03:02,A*11:01,A*11:02,A*11:04,A*11:05,A*11:13,A*11:20,A*29:01,A*29:02,A*68:01
	29943338	Other	Q	0.149	0.148	0.151	0.97	0.88	1.08	6.16E-01		1.99E-03	A*26:01,A*26:09,A*31:01,A*32:01,A*33:01,A*33:03,A*34:01,A*34:05,A*66:01,A*66:02,A*74:02
	29943338	Other	E	0.011	0.008	0.015	0.51	0.35	0.73	3.01E-04		1.13E-01	A*30:01,A*30:02,A*30:04
116	29943344	Y	Other	0.470	0.409	0.528	0.62	0.57	0.66	2.93E-36	4.23E-37	5.06E-12	A*02:01,A*02:03,A*02:05,A*02:06,A*02:07,A*02:10,A*02:11,A*02:12,A*02:13,A*02:20,A*02:30,A*02:42,A*02:44,A*02:51,A*02:74,A*23:01,A*24:02,A*24:03,A*24:07,A*24:08,A*24:10,A*24:20,A*24:25,A*24:27,A*24:63,A*69:01
	29943344	Other	D	0.459	0.401	0.513	0.63	0.59	0.68	2.09E-32		2.84E-11	A*01:01,A*01:03,A*03:01,A*03:02,A*11:01,A*11:02,A*11:04,A*11:05,A*11:13,A*11:20,A*26:01,A*26:09,A*29:01,A*29:02,A*31:01,A*32:01,A*33:01,A*33:03,A*34:01,A*34:05,A*66:01,A*66:02,A*68:01,A*74:02
	29943344	Other	H	0.011	0.008	0.015	0.51	0.35	0.73	3.01E-04		1.13E-01	A*30:01,A*30:02,A*30:04
125	29943371	A	S	0.000	0.000	0.000	NA	NA	NA	NA		NA	A*34:05
127	29943377	K	N	0.468	0.407	0.526	0.61	0.57	0.66	1.92E-36		7.96E-12	A*01:01,A*01:03,A*03:01,A*03:02,A*11:01,A*11:02,A*11:04,A*11:05,A*11:13,A*11:20,A*26:01,A*26:09,A*29:01,A*29:02,A*30:01,A*30:02,A*30:04,A*31:01,A*32:01,A*33:01,A*33:03,A*34:01,A*34:05,A*66:01,A*66:02,A*74:02
138	29943410	M	R	0.000	0.000	0.000	NA	NA	NA	NA		NA	A*24:27
142	29943422	I	T	0.365	0.424	0.309	1.65	1.53	1.78	3.64E-36		1.37E-13	A*02:01,A*02:03,A*02:05,A*02:06,A*02:07,A*02:10,A*02:11,A*02:12,A*02:13,A*02:20,A*02:30,A*02:42,A*02:51,A*02:74,A*68:01,A*69:01
144	29943428	K	Other	0.170	0.160	0.180	0.86	0.78	0.95	3.88E-03	1.24E-02	NA	A*01:01,A*01:03,A*02:01,A*02:03,A*02:05,A*02:06,A*02:07,A*02:10,A*02:11,A*02:12,A*02:13,A*02:20,A*02:30,A*02:42,A*02:44,A*02:51,A*02:74,A*03:01,A*03:02,A*11:01,A*11:02,A*11:04,A*11:20,A*24:02,A*24:03,A*24:07,A*24:08,A*24:10,A*24:20,A*24:25,A*24:27,A*24:63,A*68:01,A*69:01
	29943428	Other	Q	0.170	0.160	0.180	0.86	0.78	0.95	4.18E-03		3.70E-02	A*23:01,A*26:01,A*26:09,A*29:01,A*29:02,A*30:01,A*30:02,A*30:04,A*31:01,A*32:01,A*33:01,A*33:03,A*34:01,A*34:05,A*66:01,A*66:02,A*74:02
	29943428	Other	R	0.000	0.000	0.000	NA	NA	NA	NA		NA	A*11:05
	29943428	Other	E	0.000	0.000	0.000	NA	NA	NA	NA		NA	A*11:13
145	29943431	R	H	0.365	0.424	0.309	1.65	1.53	1.78	3.64E-36		1.37E-13	A*02:01,A*02:03,A*02:05,A*02:06,A*02:07,A*02:10,A*02:11,A*02:12,A*02:13,A*02:20,A*02:30,A*02:42,A*02:51,A*02:74,A*68:01,A*69:01
149	29943443	A	T	0.110	0.114	0.106	1.10	0.97	1.23	1.31E-01		2.71E-04	A*02:03,A*26:01,A*26:09,A*34:01,A*34:05,A*66:01,A*66:02
150	29943446	A	V	0.012	0.011	0.012	0.96	0.68	1.34	8.08E-01		1.25E-01	A*01:01,A*01:03
151	29943449	H	R	0.144	0.134	0.154	0.84	0.76	0.94	1.42E-03		7.64E-02	A*02:51,A*23:01,A*29:01,A*29:02,A*30:01,A*30:02,A*31:01,A*32:01,A*33:01,A*33:03,A*74:02
152	29943452	V	Other	0.422	0.371	0.469	0.66	0.61	0.72	4.47E-26	4.95E-34	1.83E-09	A*02:01,A*02:05,A*02:06,A*02:07,A*02:10,A*02:11,A*02:12,A*02:20,A*02:30,A*02:42,A*02:44,A*02:51,A*02:74,A*03:02,A*23:01,A*24:02,A*24:03,A*24:07,A*24:08,A*24:10,A*24:20,A*24:25,A*24:27,A*24:63,A*29:01,A*29:02,A*30:04,A*31:01,A*32:01,A*33:01,A*33:03,A*68:01,A*69:01,A*74:02
	29943452	Other	E	0.120	0.124	0.115	1.09	0.97	1.22	1.31E-01		3.16E-03	A*02:03,A*02:13,A*03:01,A*11:20,A*26:01,A*26:09,A*34:01,A*34:05,A*66:01,A*66:02
	29943452	Other	A	0.291	0.239	0.339	0.60	0.55	0.65	5.78E-32		1.12E-17	A*01:01,A*01:03,A*11:01,A*11:02,A*11:04,A*11:05,A*11:13
	29943452	Other	R	0.000	0.000	0.000	NA	NA	NA	NA		4.67E-01	A*30:01
	29943452	Other	W	0.011	0.007	0.015	0.51	0.35	0.74	3.53E-04		1.34E-01	A*30:02
156	29943464	Other	L	0.431	0.476	0.388	1.43	1.33	1.54	1.09E-20	6.46E-24	7.05E-09	A*02:01,A*02:06,A*02:07,A*02:10,A*02:11,A*02:20,A*02:30,A*02:42,A*02:51,A*02:74,A*03:01,A*23:01,A*29:01,A*29:02,A*30:01,A*30:02,A*31:01,A*32:01,A*33:01,A*33:03,A*69:01,A*74:02
	29943464	Other	Q	0.444	0.395	0.490	0.67	0.63	0.73	2.15E-24		1.30E-15	A*02:12,A*02:13,A*02:44,A*03:02,A*11:01,A*11:02,A*11:04,A*11:05,A*11:13,A*11:20,A*24:02,A*24:03,A*24:07,A*24:08,A*24:10,A*24:20,A*24:25,A*24:27,A*24:63
	29943464	Other	W	0.113	0.118	0.109	1.09	0.97	1.22	1.49E-01		4.35E-04	A*02:03,A*02:05,A*26:01,A*26:09,A*30:04,A*34:01,A*34:05,A*66:01,A*66:02,A*68:01
	29943464	Other	R	0.012	0.011	0.012	0.96	0.68	1.34	8.08E-01		1.25E-01	A*01:01,A*01:03
158	29943470	A	V	0.012	0.011	0.012	0.96	0.68	1.34	8.08E-01		1.25E-01	A*01:01,A*01:03

	161	29943479	E	D	0.010	0.010	0.010	1.01	0.70	1.47	9.50E-01		6.19E-03	A*03:01,A*03:02
	163	29943485	T	Other	0.315	0.267	0.360	0.64	0.59	0.69	9.48E-27	1.09E-25	NA	A*02:01,A*02:03,A*02:05,A*02:06,A*02:07,A*02:10,A*02:11,A*02:12,A*02:13,A*02:20,A*02:30,A*02:42,A*02:44,A*02:51,A*02:74,A*03:01,A*03:02,A*11:04,A*23:01,A*24:02,A*24:03,A*24:07,A*24:08,A*24:20,A*24:25,A*24:27,A*24:63,A*26:09,A*29:01,A*29:02,A*30:01,A*30:02,A*30:04,A*31:01,A*32:01,A*33:01,A*33:03,A*34:01,A*34:05,A*68:01,A*69:01,A*74:02
		29943485	Other	R	0.315	0.267	0.360	0.64	0.59	0.69	1.42E-26		1.05E-15	A*01:01,A*01:03,A*11:01,A*11:02,A*11:05,A*11:13,A*11:20,A*24:10,A*26:01,A*66:01
		29943485	Other	E	0.000	0.000	0.000	NA	NA	NA	NA		NA	A*66:02
	166	29943494	E	D	0.174	0.175	0.172	1.03	0.93	1.13	6.02E-01		6.51E-01	A*01:01,A*01:03,A*23:01,A*24:02,A*24:07,A*24:08,A*24:20,A*24:25,A*24:27,A*24:63
	167	29943497	W	G	0.174	0.175	0.172	1.03	0.93	1.13	6.02E-01		6.51E-01	A*01:01,A*01:03,A*23:01,A*24:02,A*24:07,A*24:08,A*24:20,A*24:25,A*24:27,A*24:63
	171	29943509	Y	H	0.000	0.000	0.000	NA	NA	NA	NA		NA	A*33:01
	175	29943521	G	R	0.000	0.000	0.000	NA	NA	NA	NA		NA	A*02:74
	184	29944126	A	P	0.402	0.457	0.350	1.56	1.44	1.68	4.24E-30		1.56E-10	A*01:01,A*01:03,A*03:01,A*03:02,A*11:01,A*11:02,A*11:04,A*11:05,A*11:13,A*23:01,A*24:02,A*24:03,A*24:07,A*24:08,A*24:10,A*24:20,A*24:25,A*24:63,A*30:01,A*30:02,A*30:04,A*31:01,A*33:01,A*33:03
	186	29944132	K	R	0.000	0.000	0.000	NA	NA	NA	NA			A*33:01
	193	29944153	A	P	0.478	0.425	0.528	0.66	0.61	0.71	7.52E-28		5.50E-19	A*01:01,A*01:03,A*03:01,A*03:02,A*11:01,A*11:02,A*11:04,A*11:05,A*11:13,A*23:01,A*24:02,A*24:03,A*24:07,A*24:08,A*24:10,A*24:20,A*24:25,A*24:63,A*30:01,A*30:02,A*30:04
	194	29944156	V	I	0.478	0.425	0.528	0.66	0.61	0.71	7.52E-28		5.50E-19	A*01:01,A*01:03,A*03:01,A*03:02,A*11:01,A*11:02,A*11:04,A*11:05,A*11:13,A*23:01,A*24:02,A*24:03,A*24:07,A*24:08,A*24:10,A*24:20,A*24:25,A*24:63,A*30:01,A*30:02,A*30:04
	207	29944195	S	G	0.478	0.425	0.528	0.66	0.61	0.71	7.52E-28		5.50E-19	A*01:01,A*01:03,A*03:01,A*03:02,A*11:01,A*11:02,A*11:04,A*11:05,A*11:13,A*23:01,A*24:02,A*24:03,A*24:07,A*24:08,A*24:10,A*24:20,A*24:25,A*24:63,A*30:01,A*30:02,A*30:04
	245	29944309	A	V	0.002	0.002	0.002	0.98	0.42	2.31	9.64E-01		5.40E-02	A*68:01
	246	29944312	A	S	0.157	0.150	0.163	0.91	0.82	1.01	7.21E-02		1.64E-02	A*26:01,A*26:09,A*29:01,A*29:02,A*31:01,A*32:01,A*33:01,A*33:03,A*34:01,A*34:05,A*66:01,A*66:02,A*74:02
	253	29944333	Q	E	0.478	0.425	0.528	0.66	0.61	0.71	7.52E-28		5.50E-19	A*01:01,A*01:03,A*03:01,A*03:02,A*11:01,A*11:02,A*11:04,A*11:05,A*11:13,A*23:01,A*24:02,A*24:03,A*24:07,A*24:08,A*24:10,A*24:20,A*24:25,A*24:63,A*30:01,A*30:02,A*30:04
	276	29944504	P	L	0.311	0.256	0.363	0.59	0.55	0.65	8.18E-35		3.29E-23	A*01:01,A*01:03,A*03:01,A*03:02,A*11:01,A*11:02,A*11:04,A*11:05,A*30:01,A*30:02,A*30:04
	282	29944522	I	V	0.166	0.168	0.164	1.03	0.94	1.14	5.37E-01		7.00E-01	A*23:01,A*24:02,A*24:03,A*24:07,A*24:08,A*24:10,A*24:20,A*24:25
	283	29944525	P	H	0.002	0.002	0.002	0.79	0.33	1.87	5.90E-01		6.47E-01	A*23:01
	288	29944540	I	L	0.004	0.001	0.007	0.18	0.08	0.42	8.60E-05		2.58E-01	A*34:01,A*34:05
	294	29944558	F	L	0.478	0.425	0.528	0.65	0.61	0.71	5.62E-28		5.31E-19	A*01:01,A*01:03,A*03:01,A*03:02,A*11:01,A*11:02,A*11:04,A*11:05,A*23:01,A*24:02,A*24:03,A*24:07,A*24:08,A*24:10,A*24:20,A*24:25,A*30:01,A*30:02,A*30:04
	297	29944567	V	M	0.004	0.003	0.004	0.87	0.47	1.60	6.59E-01		1.02E-04	A*32:01,A*74:02
	298	29944570	I	F	0.131	0.124	0.137	0.89	0.80	0.99	3.71E-02		3.52E-02	A*29:01,A*29:02,A*31:01,A*32:01,A*33:01,A*33:03,A*74:02
	299	29944573	T	A	0.157	0.150	0.163	0.91	0.82	1.01	7.21E-02		1.75E-02	A*26:01,A*26:09,A*29:01,A*29:02,A*31:01,A*32:01,A*33:01,A*33:03,A*34:01,A*34:05,A*66:01,A*66:02,A*74:02
	307	29944597	M	R	0.131	0.124	0.137	0.89	0.80	0.99	3.71E-02		3.52E-02	A*29:01,A*29:02,A*31:01,A*32:01,A*33:01,A*33:03,A*74:02
	311	29944609	K	N	0.166	0.168	0.164	1.03	0.94	1.14	5.37E-01		7.00E-01	A*23:01,A*24:02,A*24:03,A*24:07,A*24:08,A*24:10,A*24:20,A*24:25
	321	29945081	S	T	0.311	0.256	0.363	0.60	0.55	0.65	1.19E-34		3.67E-20	A*01:01,A*01:03,A*03:01,A*03:02,A*11:01,A*11:02,A*11:04,A*30:01,A*30:02,A*30:04
	334	29945262	V	M	0.157	0.150	0.163	0.91	0.82	1.01	7.21E-02		1.75E-02	A*26:01,A*26:09,A*29:01,A*29:02,A*31:01,A*32:01,A*33:01,A*33:03,A*34:01,A*34:05,A*66:01,A*66:02,A*74:02
HLA-C	339	31269347	A	T	0.190	0.187	0.192	0.97	0.89	1.07	5.57E-01		1.18E-04	C*07:01,C*07:02,C*07:04,C*07:06,C*07:56
	326	31269386	S	C	0.190	0.187	0.192	0.97	0.89	1.07	5.57E-01		1.18E-04	C*07:01,C*07:02,C*07:04,C*07:06,C*07:56
	324	31269499	A	V	0.012	0.014	0.009	1.54	1.09	2.18	1.53E-02		NA	C*07:06
	315	31269526	C	H	0.000	0.000	0.000	0.52	0.05	5.73	5.93E-01		NA	C*17:01
	314	31269969	M	I	0.000	0.000	0.000	0.52	0.05	5.73	5.93E-01		NA	C*17:01
	313	31269972	V	Other	0.190	0.187	0.192	0.97	0.89	1.07	5.57E-01	2.54E-02	NA	C*01:02,C*01:03,C*01:06,C*01:08,C*01:30,C*02:02,C*03:02,C*03:03,C*03:04,C*03:13,C*03:17,C*03:21,C*03:56,C*04:01,C*04:03,C*04:06,C*05:01,C*06:02,C*06:03,C*07:27,C*08:01,C*08:02,C*08:03,C*12:02,C*12:03,C*12:04,C*14:02,C*14:03,C*15:02,C*15:04,C*15:05,C*16:02,C*16:04,C*17:01
		31269972	Other	M	0.178	0.173	0.183	0.94	0.85	1.03	1.99E-01		NA	C*07:01,C*07:02,C*07:04,C*07:56
		31269972	Other	K	0.012	0.014	0.009	1.54	1.09	2.18	1.53E-02		NA	C*07:06
	312	31269975	V	A	0.190	0.188	0.192	0.97	0.88	1.07	5.42E-01		NA	C*07:01,C*07:02,C*07:04,C*07:06,C*07:56,C*17:01
	311	31269978	A	T	0.190	0.187	0.192	0.97	0.89	1.07	5.57E-01		NA	C*07:01,C*07:02,C*07:04,C*07:06,C*07:56
	310	31269981	V	M	0.236	0.214	0.257	0.79	0.72	0.86	6.68E-08		NA	C*05:01,C*06:02,C*08:01,C*08:02,C*08:03,C*12:02,C*12:03,C*12:04,C*15:02,C*15:04,C*15:05
	309	31269984	V	M	0.046	0.044	0.049	0.90	0.76	1.07	2.21E-01		NA	C*04:01
	301	31270008	A	V	0.190	0.187	0.192	0.97	0.89	1.07	5.57E-01		NA	C*07:01,C*07:02,C*07:04,C*07:06,C*07:56
	291	31270038	L	P	0.000	0.000	0.000	0.52	0.05	5.73	5.93E-01		NA	C*17:01
	289	31270044	A	S	0.000	0.000	0.000	0.52	0.05	5.73	5.93E-01		NA	C*17:01
	285	31270056	V	Other	0.190	0.188	0.192	0.97	0.88	1.07	5.42E-01	7.51E-01	NA	C*01:02,C*01:03,C*01:06,C*01:08,C*01:30,C*02:02,C*03:02,C*03:03,C*03:04,C*03:13,C*03:17,C*03:21,C*03:56,C*04:01,C*04:03,C*04:06,C*05:01,C*06:02,C*06:03,C*07:27,C*08:01,C*08:02,C*08:03,C*12:02,C*12:03,C*12:04,C*14:02,C*14:03,C*15:02,C*15:04,C*15:05,C*16:02,C*16:04
		31270056	Other	M	0.190	0.187	0.192	0.97	0.89	1.07	5.57E-01		1.18E-04	C*07:01,C*07:02,C*07:04,C*07:06,C*07:56
		31270056	Other	L	0.000	0.000	0.000	0.52	0.05	5.73	5.93E-01		NA	C*17:01
	284	31270059	I	N	0.000	0.000	0.000	0.52	0.05	5.73	5.93E-01		NA	C*17:01
	275	31270086	E	Other	0.207	0.204	0.210	0.97	0.88	1.06	4.45E-01	1.80E-01	4.71E-01	C*01:02,C*01:03,C*01:06,C*01:08,C*01:30,C*02:02,C*03:02,C*03:03,C*03:04,C*03:13,C*03:17,C*03:21,C*03:56,C*06:02,C*06:03,C*07:01,C*07:02,C*07:04,C*07:06,C*07:27,C*07:56,C*12:02,C*12:03,C*12:04,C*14:02,C*14:03,C*15:02,C*15:04,C*15:05,C*16:02,C*16:04

	31270086	Other	K	0.068	0.064	0.072	0.88	0.76	1.02	8.94E-02		1.25E-01	C*04:01,C*04:03,C*04:06,C*17:01
	31270086	Other	G	0.139	0.140	0.138	1.02	0.92	1.13	7.25E-01		8.37E-01	C*05:01,C*08:01,C*08:02,C*08:03
273	31270216	R	S	0.190	0.187	0.192	0.97	0.89	1.07	5.57E-01		1.18E-04	C*07:01,C*07:02,C*07:04,C*07:06,C*07:56
270	31270225	L	C	0.000	0.000	0.000	0.52	0.05	5.73	5.93E-01		NA	C*17:01
267	31270234	P	Q	0.190	0.188	0.192	0.97	0.88	1.07	5.42E-01		1.18E-04	C*07:01,C*07:02,C*07:04,C*07:06,C*07:56,C*17:01
261	31270252	V	M	0.190	0.187	0.192	0.97	0.89	1.07	5.57E-01		1.18E-04	C*07:01,C*07:02,C*07:04,C*07:06,C*07:56
253	31270276	E	Q	0.190	0.188	0.192	0.97	0.88	1.07	5.42E-01		1.18E-04	C*07:01,C*07:02,C*07:04,C*07:06,C*07:56,C*17:01
248	31270291	V	M	0.234	0.282	0.190	1.65	1.51	1.80	5.03E-29		1.93E-02	C*01:02,C*01:03,C*01:06,C*01:08,C*01:30
219	31270378	R	W	0.433	0.410	0.455	0.84	0.78	0.90	1.75E-06		8.80E-01	C*01:02,C*01:03,C*01:06,C*01:08,C*01:30,C*03:02,C*03:03,C*03:04,C*03:13,C*03:17,C*04:01,C*04:03,C*04:06,C*14:02,C*14:03
211	31270402	A	T	0.005	0.004	0.005	0.91	0.53	1.57	7.35E-01		1.44E-01	C*02:02
194	31270453	V	L	0.190	0.187	0.192	0.97	0.89	1.07	5.57E-01		1.18E-04	C*07:01,C*07:02,C*07:04,C*07:06,C*07:56
193	31270456	P	L	0.002	0.004	0.000	11.73	2.75	49.94	8.68E-04		5.67E-01	C*16:02,C*16:04
184	31270483	H	Other	0.190	0.188	0.192	0.97	0.88	1.07	5.42E-01	7.51E-01	1.18E-04	C*01:02,C*01:03,C*01:06,C*01:08,C*01:30,C*02:02,C*03:02,C*03:03,C*03:04,C*03:13,C*03:17,C*03:21,C*03:56,C*04:01,C*04:03,C*04:06,C*05:01,C*06:02,C*06:03,C*07:27,C*08:01,C*08:02,C*08:03,C*12:02,C*12:03,C*12:04,C*14:02,C*14:03,C*15:02,C*15:04,C*15:05,C*16:02,C*16:04
	31270483	Other	P	0.190	0.187	0.192	0.97	0.89	1.07	5.57E-01		5.64E-01	C*07:01,C*07:02,C*07:04,C*07:06,C*07:56
	31270483	Other	R	0.000	0.000	0.000	0.52	0.05	5.73	5.93E-01		NA	C*17:01
177	31271091	E	K	0.146	0.148	0.144	1.03	0.93	1.15	5.53E-01		5.64E-01	C*05:01,C*07:04,C*08:01,C*08:02,C*08:03
175	31271097	G	R	0.002	0.003	0.001	2.57	1.07	6.22	3.57E-02		NA	C*08:03
173	31271103	E	K	0.229	0.210	0.248	0.80	0.74	0.88	1.35E-06		6.35E-01	C*03:02,C*03:03,C*03:04,C*03:13,C*03:17,C*03:21,C*03:56
171	31271109	Y	H	0.000	0.000	0.000	NA	NA	NA	NA		NA	C*01:30
170	31271112	R	G	0.000	0.000	0.000	0.52	0.05	5.73	5.93E-01		NA	C*17:01
163	31271133	T	Other	0.234	0.214	0.253	0.81	0.74	0.88	1.31E-06	6.43E-06	5.49E-01	C*01:02,C*01:03,C*01:06,C*01:08,C*01:30,C*03:21,C*04:01,C*04:03,C*04:06,C*05:01,C*06:02,C*06:03,C*07:01,C*07:02,C*07:04,C*07:06,C*07:27,C*07:56,C*08:01,C*08:02,C*08:03,C*12:02,C*12:03,C*12:04,C*14:02,C*14:03,C*15:02,C*15:04,C*15:05,C*16:02,C*16:04
	31271133	Other	L	0.229	0.210	0.248	0.81	0.74	0.88	1.49E-06		6.35E-01	C*03:02,C*03:03,C*03:04,C*03:13,C*03:17,C*03:56
	31271133	Other	E	0.005	0.005	0.005	0.88	0.52	1.51	6.48E-01		1.44E-01	C*02:02,C*17:01
156	31271154	L	Other	0.422	0.454	0.391	1.30	1.20	1.40	1.09E-11	2.34E-24	5.88E-02	C*03:02,C*03:03,C*03:04,C*03:13,C*03:17,C*03:56,C*04:06,C*07:01,C*07:02,C*07:06,C*07:27,C*07:56,C*08:01,C*08:03,C*15:02,C*15:04,C*15:05,C*17:01
	31271154	Other	R	0.337	0.380	0.297	1.44	1.33	1.56	3.81E-20		4.92E-01	C*01:02,C*01:03,C*01:06,C*01:08,C*01:30,C*04:01,C*04:03,C*05:01,C*08:02,C*14:02,C*14:03
	31271154	Other	W	0.075	0.063	0.087	0.70	0.61	0.81	8.94E-07		1.35E-09	C*02:02,C*03:21,C*06:02,C*06:03,C*12:02,C*12:03,C*12:04,C*16:04
	31271154	Other	D	0.007	0.008	0.006	1.26	0.81	1.95	3.04E-01		1.22E-01	C*07:04
	31271154	Other	Q	0.002	0.004	0.000	11.19	2.62	47.77	1.11E-03		7.35E-01	C*16:02
152	31271166	E	Other	0.331	0.332	0.330	1.01	0.93	1.09	8.28E-01	7.03E-01	7.42E-04	C*01:02,C*01:03,C*01:08,C*01:30,C*02:02,C*03:02,C*03:03,C*03:04,C*03:13,C*03:17,C*03:21,C*03:56,C*04:01,C*04:03,C*04:06,C*05:01,C*06:02,C*06:03,C*08:02,C*12:02,C*12:03,C*12:04,C*14:02,C*14:03,C*15:02,C*15:04,C*15:05,C*17:01
	31271166	Other	A	0.192	0.191	0.193	0.99	0.90	1.09	8.69E-01		1.95E-04	C*07:01,C*07:02,C*07:04,C*07:06,C*07:27,C*07:56,C*16:02,C*16:04
	31271166	Other	T	0.138	0.140	0.137	1.03	0.93	1.15	5.72E-01		7.89E-01	C*08:01,C*08:03
	31271166	Other	V	0.001	0.000	0.001	0.43	0.08	2.20	3.09E-01		3.03E-01	C*01:06
151	31271169	R	C	0.001	0.001	0.001	1.06	0.37	3.03	9.10E-01		NA	C*01:08
147	31271181	W	L	0.190	0.188	0.193	0.97	0.88	1.06	4.97E-01		1.59E-04	C*07:01,C*07:02,C*07:04,C*07:06,C*07:27,C*07:56,C*17:01
143	31271193	T	S	0.000	0.000	0.000	0.52	0.05	5.73	5.93E-01		NA	C*17:01
138	31271208	T	K	0.001	0.000	0.002	0.12	0.01	0.92	4.17E-02		6.48E-01	C*05:01,C*08:02
121	31271193	K	M	0.386	0.378	0.394	0.93	0.87	1.01	7.36E-02		NA	C*03:56
116	31271274	Other	S	0.224	0.217	0.232	0.92	0.84	1.00	5.69E-02	1.27E-07	3.45E-02	C*02:02,C*03:02,C*06:02,C*06:03,C*07:01,C*07:02,C*07:06,C*07:27,C*07:56,C*12:02,C*12:03,C*12:04,C*14:02,C*14:03,C*15:04,C*16:02,C*16:04
	31271274	Other	F	0.368	0.390	0.347	1.20	1.11	1.29	3.48E-06		2.05E-01	C*01:03,C*04:01,C*04:03,C*04:06,C*05:01,C*07:04,C*08:01,C*08:02,C*08:03,C*15:05,C*17:01
	31271274	Other	Y	0.021	0.016	0.027	0.58	0.45	0.76	6.98E-05		4.09E-01	C*01:02,C*01:06,C*01:08,C*01:30,C*03:03,C*03:04,C*03:13,C*03:17,C*03:21,C*03:56
	31271274	Other	L	0.212	0.209	0.215	0.96	0.88	1.05	4.28E-01		1.59E-04	C*15:02
114	31271280	D	N	0.027	0.016	0.037	0.41	0.32	0.53	8.96E-12		3.80E-01	C*01:03,C*04:01,C*04:03,C*04:06,C*05:01,C*08:01,C*08:02,C*08:03,C*17:01
113	31271283	Y	H	0.229	0.210	0.248	0.80	0.74	0.88	1.35E-06		5.67E-03	C*15:02,C*15:05
103	31271313	L	V	0.492	0.455	0.527	0.74	0.69	0.80	9.67E-15		6.35E-01	C*03:02,C*03:03,C*03:04,C*03:13,C*03:17,C*03:21,C*03:56
99	31271325	Other	Y	0.103	0.099	0.107	0.92	0.81	1.04	1.70E-01	4.86E-29	3.38E-04	C*02:02,C*03:02,C*03:03,C*03:04,C*03:13,C*03:17,C*03:21,C*03:56,C*05:01,C*06:02,C*06:03,C*07:01,C*07:04,C*07:06,C*07:27,C*08:01,C*08:02,C*08:03,C*12:02,C*12:03,C*12:04,C*15:02,C*15:04,C*15:05,C*16:02,C*16:04,C*17:01
	31271325	Other	F	0.234	0.282	0.190	1.65	1.51	1.80	5.03E-29		2.36E-02	C*04:01,C*04:03,C*04:06,C*14:02,C*14:03
	31271325	Other	C	0.171	0.165	0.176	0.92	0.84	1.02	1.17E-01		2.07E-02	C*01:02,C*01:03,C*01:06,C*01:08,C*01:30
	31271325	Other	S	0.319	0.360	0.280	1.44	1.33	1.55	2.72E-19		1.70E-04	C*07:02,C*07:56
97	31271331	R	W	0.171	0.136	0.204	0.61	0.55	0.68	1.06E-21		9.55E-01	C*01:02,C*01:03,C*01:06,C*01:08,C*01:30,C*03:17,C*06:02,C*06:03,C*12:03,C*12:04,C*14:02,C*14:03,C*16:02,C*16:04
95	31271337	L	Other	0.164	0.128	0.198	0.59	0.53	0.66	2.65E-23	1.10E-22	1.50E-05	C*01:02,C*01:03,C*01:06,C*01:08,C*01:30,C*02:02,C*03:02,C*03:13,C*03:17,C*04:01,C*04:03,C*04:06,C*05:01,C*06:02,C*06:03,C*07:01,C*07:02,C*07:06,C*07:27,C*07:56,C*08:01,C*08:02,C*08:03,C*12:02,C*12:03,C*12:04,C*14:02,C*14:03,C*16:02,C*16:04
	31271337	Other	I	0.007	0.008	0.006	1.26	0.81	1.95	3.04E-01		4.48E-06	C*03:03,C*03:04,C*03:21,C*03:56,C*15:02,C*15:04,C*15:05,C*17:01

		31271337	Other	F									1.22E-01	C*07:04
	94	31271340	T	I	0.255	0.225	0.284	0.73	0.67	0.80	8.29E-13		1.82E-01	C*03:02,C*03:03,C*03:04,C*03:21,C*03:56,C*15:02,C*15:04,C*15:05
	91	31271349	G	R	0.033	0.030	0.035	0.85	0.69	1.05	1.36E-01		9.47E-02	C*03:03,C*03:13,C*03:21,C*03:56,C*07:56
	90	31271602	A	D	0.284	0.274	0.294	0.91	0.84	0.99	2.19E-02		2.12E-02	C*04:01,C*04:03,C*04:06,C*06:02,C*06:03,C*07:01,C*07:02,C*07:04,C*07:06,C*07:27,C*07:56
	80	31271632	N	K	0.129	0.111	0.145	0.74	0.66	0.83	8.57E-08		7.64E-04	C*02:02,C*04:01,C*04:03,C*04:06,C*05:01,C*06:02,C*06:03,C*12:04,C*15:02,C*15:04,C*15:05,C*16:02,C*17:01
	77	31271641	S	N	0.129	0.111	0.145	0.74	0.66	0.83	8.57E-08		7.64E-04	C*02:02,C*04:01,C*04:03,C*04:06,C*05:01,C*06:02,C*06:03,C*12:04,C*15:02,C*15:04,C*15:05,C*16:02,C*17:01
	73	31271653	T	A	0.328	0.309	0.346	0.85	0.78	0.92	3.09E-05		9.27E-01	C*04:01,C*04:03,C*04:06,C*06:02,C*06:03,C*07:01,C*07:02,C*07:04,C*07:06,C*07:27,C*07:56,C*12:02,C*12:03,C*12:04, C*17:01
	66	31271674	K	N	0.039	0.030	0.046	0.64	0.53	0.78	1.08E-05		7.22E-03	C*07:01,C*07:06,C*15:02,C*15:04,C*15:05
	49	31271725	A	E	0.046	0.044	0.049	0.90	0.76	1.07	2.21E-01		9.70E-01	C*04:01
	35	31271767	R	Q	0.139	0.140	0.138	1.02	0.92	1.13	7.25E-01		7.72E-01	C*05:01,C*08:01,C*08:02,C*08:03
	24	31271800	A	S	0.451	0.492	0.412	1.39	1.29	1.50	1.18E-17		3.88E-06	C*01:02,C*01:03,C*01:06,C*01:08,C*01:30,C*06:02,C*06:03,C*07:01,C*07:02,C*07:04,C*07:06,C*07:27,C*07:56
	21	31271809	R	H	0.284	0.250	0.315	0.73	0.67	0.79	2.74E-14		3.45E-02	C*02:02,C*03:02,C*03:03,C*03:04,C*03:13,C*03:17,C*03:21,C*03:56,C*04:03,C*04:06,C*14:03,C*15:02,C*15:04,C*15:05
	16	31271824	G	S	0.026	0.024	0.028	0.87	0.69	1.10	2.40E-01		9.11E-03	C*02:02,C*04:03,C*04:06
	14	31271830	R	W	0.046	0.044	0.049	0.90	0.76	1.07	2.21E-01		9.70E-01	C*04:01
	11	31271839	A	S	0.316	0.361	0.274	1.48	1.37	1.60	2.67E-22		1.58E-01	C*01:02,C*01:03,C*01:06,C*01:08,C*01:30,C*04:01,C*14:02,C*14:03
	9	31271845	Other	Y	0.468	0.429	0.504	0.73	0.68	0.79	8.48E-16	1.22E-29	8.10E-05	C*02:02,C*03:02,C*03:03,C*03:04,C*03:13,C*03:17,C*03:21,C*03:56,C*04:03,C*04:06,C*05:01,C*06:03,C*08:01,C*08:02, C*08:03,C*12:02,C*12:03,C*12:04,C*15:02,C*15:04,C*15:05,C*16:02,C*16:04,C*17:01
		31271845	Other	D	0.216	0.210	0.222	0.93	0.86	1.02	1.35E-01		1.07E-03	C*06:02,C*07:01,C*07:02,C*07:04,C*07:06,C*07:27,C*07:56
		31271845	Other	F	0.234	0.282	0.190	1.65	1.51	1.80	5.03E-29		2.07E-02	C*01:02,C*01:03,C*01:06,C*01:08,C*01:30
		31271845	Other	S	0.082	0.079	0.084	0.94	0.82	1.07	3.55E-01		2.09E-01	C*04:01,C*14:02,C*14:03
	6	31271854	R	K	0.234	0.282	0.190	1.65	1.51	1.80	5.03E-29		2.07E-02	C*01:02,C*01:03,C*01:06,C*01:08,C*01:30
	1	31271869	C	G	0.296	0.272	0.318	0.80	0.74	0.87	1.02E-07		2.36E-01	C*03:02,C*03:03,C*03:04,C*03:13,C*04:01,C*04:03,C*04:06,C*17:01
	-5	31272014	T	I	0.000	0.000	0.000	0.52	0.05	5.73	5.93E-01		NA	C*17:01
	-9	31272026	A	G	0.190	0.187	0.192	0.97	0.89	1.07	5.57E-01		1.18E-04	C*07:01,C*07:02,C*07:04,C*07:06,C*07:56
	-15	31272044	I	L	0.221	0.208	0.234	0.86	0.79	0.94	8.52E-04		8.55E-03	C*02:02,C*07:01,C*07:02,C*07:04,C*07:06,C*07:56,C*15:02,C*15:04,C*15:05,C*17:01
	-17	31272050	I	A	0.190	0.188	0.192	0.97	0.88	1.07	5.42E-01		1.18E-04	C*07:01,C*07:02,C*07:04,C*07:06,C*07:56,C*17:01
	-18	31272053	R	Q	0.000	0.000	0.000	0.52	0.05	5.73	5.93E-01		NA	C*17:01
HLA-B	325	31354527	S	C	0.315	0.278	0.351	0.71	0.66	0.77	1.18E-16		1.31E-05	B*07:02,B*07:05,B*07:53,B*08:01,B*13:01,B*13:02,B*27:02,B*27:04,B*27:05,B*27:06,B*27:07,B*40:01,B*40:02,B*40:03, B*40:06,B*40:10,B*40:40,B*40:50,B*41:01,B*41:02,B*44:02,B*44:03,B*47:01,B*48:01,B*48:03,B*48:04,B*57:01,B*81:01
	307	31355128	M	V	0.000	0.000	0.000	NA	NA	NA	NA		NA	B*47:01
	305	31355134	T	A	0.388	0.348	0.427	0.72	0.67	0.78	1.35E-17		5.23E-01	B*07:02,B*07:05,B*07:53,B*08:01,B*13:01,B*13:02,B*14:01,B*14:02,B*27:02,B*27:04,B*27:05,B*27:06,B*27:07,B*38:01, B*38:02,B*39:01,B*39:05,B*39:06,B*39:09,B*39:15,B*40:01,B*40:02,B*40:03,B*40:06,B*40:10,B*40:40,B*40:50,B*41:01, B*41:02,B*44:02,B*44:03,B*47:01,B*48:01,B*48:03,B*48:04,B*57:01,B*67:01,B*81:01
	282	31355203	I	V	0.382	0.348	0.415	0.76	0.70	0.81	3.50E-13		9.15E-01	B*07:02,B*07:53,B*08:01,B*13:01,B*13:02,B*14:01,B*14:02,B*27:02,B*27:04,B*27:05,B*27:06,B*27:07,B*38:01,B*38:02, B*39:01,B*39:05,B*39:06,B*39:09,B*39:15,B*40:01,B*40:02,B*40:03,B*40:06,B*40:10,B*40:40,B*40:50,B*41:01,B*41:02, B*44:02,B*44:03,B*47:01,B*48:01,B*48:03,B*48:04,B*57:01,B*67:01,B*81:01
	245	31355407	A	T	0.023	0.026	0.020	1.29	1.01	1.65	4.42E-02		7.84E-01	B*48:01,B*48:03,B*48:04,B*81:01
	211	31355509	A	G	0.016	0.008	0.023	0.35	0.25	0.49	1.95E-09		1.96E-04	B*27:04,B*27:06
	199	31355545	A	V	0.015	0.017	0.013	1.25	0.92	1.69	1.58E-01		5.90E-01	B*44:02,B*44:03
	194	31355560	I	V	0.185	0.190	0.180	1.07	0.97	1.18	1.54E-01		3.38E-02	B*35:01,B*35:02,B*35:03,B*35:05,B*35:08,B*51:01,B*51:02,B*51:03,B*51:06,B*51:07,B*52:01,B*53:01,B*58:01,B*78:02
	180	31356176	Q	E	0.178	0.171	0.184	0.91	0.83	1.01	6.74E-02		7.72E-01	B*07:02,B*07:05,B*08:01,B*40:01,B*40:10,B*41:01,B*41:02,B*48:01,B*48:03,B*81:01
	178	31356182	T	K	0.175	0.170	0.181	0.93	0.84	1.02	1.17E-01		9.64E-01	B*07:02,B*07:05,B*40:01,B*40:10,B*48:01,B*48:03,B*81:01
	177	31356185	E	D	0.178	0.171	0.184	0.91	0.83	1.01	6.74E-02		7.72E-01	B*07:02,B*07:05,B*08:01,B*40:01,B*40:10,B*41:01,B*41:02,B*48:01,B*48:03,B*81:01
	171	31356203	Y	H	0.054	0.059	0.049	1.21	1.03	1.43	1.79E-02		1.53E-01	B*14:01,B*14:02,B*18:01,B*18:02,B*51:01,B*51:03,B*51:06,B*51:07,B*52:01,B*78:02
	167	31356215	W	Other	0.025	0.029	0.022	1.36	1.08	1.72	9.41E-03	1.74E-02	1.72E-01	B*07:02,B*07:05,B*07:53,B*08:01,B*13:01,B*13:02,B*14:01,B*14:02,B*15:01,B*15:02,B*15:03,B*15:05,B*15:06,B*15:07, B*15:08,B*15:10,B*15:11,B*15:13,B*15:17,B*15:18,B*15:21,B*15:25,B*15:27,B*15:29,B*15:32,B*15:35,B*15:46,B*15:58, B*15:86,B*18:01,B*18:02,B*27:02,B*27:04,B*27:05,B*27:06,B*27:07,B*35:01,B*35:02,B*35:03,B*35:05,B*35:08,B*35:43, B*37:01,B*38:01,B*38:02,B*38:15,B*39:01,B*39:05,B*39:06,B*39:09,B*39:15,B*40:01,B*40:02,B*40:03,B*40:06,B*40:10, B*40:40,B*40:50,B*41:01,B*41:02,B*45:04,B*46:01,B*46:13,B*47:01,B*48:01,B*48:03,B*48:04,B*49:01,B*50:01,B*51:01, B*51:02,B*51:06,B*51:07,B*52:01,B*53:01,B*54:01,B*55:01,B*55:02,B*55:04,B*55:07,B*55:08,B*55:12,B*55:46,B*56:01, B*56:02,B*56:03,B*56:04,B*57:01,B*58:01,B*59:01,B*67:01,B*78:02,B*81:01
		31356215	Other	S	0.016	0.018	0.014	1.23	0.92	1.64	1.71E-01		5.20E-01	B*44:02,B*44:03,B*45:01
		31356215	Other	G	0.009	0.012	0.007	1.62	1.10	2.39	1.38E-02		2.10E-01	B*15:12,B*51:03
	166	31356218	E	D	0.009	0.012	0.007	1.60	1.09	2.35	1.75E-02		2.10E-01	B*15:12
	163	31356227	L	Other	0.427	0.371	0.480	0.64	0.59	0.69	2.81E-31	8.32E-31	2.62E-02	B*15:01,B*15:02,B*15:03,B*15:05,B*15:06,B*15:07,B*15:08,B*15:10,B*15:11,B*15:12,B*15:13,B*15:17,B*15:18,B*15:21, B*15:25,B*15:27,B*15:29,B*15:32,B*15:35,B*15:46,B*15:58,B*35:01,B*35:02,B*35:03,B*35:05,B*35:08,B*35:43,B*44:02, B*44:03,B*45:01,B*45:04,B*46:01,B*46:13,B*49:01,B*50:01,B*51:01,B*51:02,B*51:03,B*51:06,B*51:07,B*52:01,B*53:01, B*55:08,B*56:01,B*56:02,B*56:03,B*56:04,B*57:01,B*58:01,B*78:02
		31356227	Other	T	0.132	0.114	0.150	0.73	0.65	0.81	1.78E-08		7.99E-02	B*08:01,B*14:01,B*14:02,B*15:86,B*18:01,B*18:02,B*37:01,B*38:01,B*38:02,B*38:15,B*39:01,B*39:05,B*39:06,B*39:09, B*39:15,B*41:01,B*41:02,B*54:01,B*55:01,B*55:02,B*55:04,B*55:07,B*55:12,B*55:46,B*59:01,B*67:01

	31356227	Other	E	0.295	0.257	0.331	0.70	0.65	0.76	1.48E-17		1.06E-04	B*07:02,B*07:05,B*07:53,B*13:01,B*13:02,B*27:02,B*27:04,B*27:05,B*27:06,B*27:07,B*40:01,B*40:02,B*40:03,B*40:06,B*40:10,B*40:40,B*40:50,B*47:01,B*48:01,B*48:03,B*48:04,B*81:01
158	31356242	A	T	0.073	0.070	0.076	0.92	0.80	1.06	2.39E-01		5.96E-08	B*38:01,B*38:02,B*38:15,B*39:01,B*39:05,B*39:06,B*39:09,B*39:15,B*67:01
156	31356248	L	Other	0.258	0.300	0.218	1.51	1.39	1.64	5.34E-22	8.03E-52	1.35E-01	B*13:01,B*13:02,B*14:01,B*14:02,B*15:02,B*15:03,B*15:05,B*15:06,B*15:10,B*15:13,B*15:17,B*15:18,B*15:21,B*15:25,B*15:29,B*15:86,B*18:01,B*18:02,B*27:02,B*27:04,B*27:05,B*27:06,B*27:07,B*35:01,B*35:02,B*35:03,B*35:05,B*38:01,B*38:02,B*38:15,B*39:01,B*39:05,B*39:06,B*39:09,B*39:15,B*40:01,B*40:02,B*40:03,B*40:06,B*40:10,B*40:40,B*40:50,B*44:03,B*46:13,B*47:01,B*48:01,B*48:03,B*48:04,B*49:01,B*50:01,B*51:01,B*51:02,B*51:03,B*51:06,B*51:07,B*52:01,B*53:01,B*54:01,B*55:01,B*55:02,B*55:04,B*55:07,B*55:08,B*55:12,B*55:46,B*56:01,B*56:02,B*56:04,B*57:01,B*58:01,B*59:01,B*67:01,B*78:02,B*81:01
	31356248	Other	W	0.238	0.291	0.189	1.73	1.58	1.88	1.09E-34		5.56E-03	B*15:01,B*15:07,B*15:08,B*15:11,B*15:12,B*15:27,B*15:32,B*15:35,B*15:46,B*15:58,B*35:43,B*46:01,B*56:03
	31356248	Other	D	0.011	0.009	0.012	0.69	0.47	0.99	4.44E-02		3.30E-02	B*08:01,B*37:01,B*41:01,B*41:02,B*44:02,B*45:01,B*45:04
	31356248	Other	R	0.009	0.000	0.017	0.02	0.01	0.08	6.10E-08		5.45E-05	B*07:02,B*07:05,B*07:53,B*35:08
152	31356260	V	E	0.437	0.472	0.405	1.31	1.22	1.41	8.82E-13		2.98E-01	B*07:02,B*07:05,B*07:53,B*14:01,B*14:02,B*15:01,B*15:02,B*15:03,B*15:06,B*15:07,B*15:08,B*15:10,B*15:11,B*15:12,B*15:13,B*15:17,B*15:18,B*15:21,B*15:25,B*15:27,B*15:29,B*15:32,B*15:35,B*15:46,B*15:58,B*27:04,B*27:06,B*35:43,B*46:01,B*49:01,B*50:01,B*51:01,B*51:02,B*51:03,B*51:06,B*51:07,B*52:01,B*55:01,B*56:03,B*78:02
147	31356275	W	L	0.167	0.169	0.165	1.03	0.94	1.14	5.36E-01		3.59E-01	B*40:01,B*40:10,B*48:01,B*48:03,B*48:04,B*81:01
145	31356281	R	L	0.075	0.053	0.096	0.53	0.46	0.61	1.94E-17		6.55E-07	B*13:01,B*13:02
143	31356287	T	S	0.167	0.169	0.165	1.03	0.94	1.14	5.36E-01		3.59E-01	B*40:01,B*40:10,B*48:01,B*48:03,B*48:04,B*81:01
131	31356323	S	R	0.205	0.196	0.213	0.91	0.83	0.99	3.17E-02		7.37E-01	B*07:02,B*07:05,B*07:53,B*08:01,B*27:07,B*40:01,B*40:02,B*40:03,B*40:06,B*40:10,B*40:40,B*40:50,B*41:01,B*41:02,B*48:01,B*48:03,B*48:04,B*55:04,B*55:08,B*81:01
116	31356368	Other	S	0.480	0.535	0.428	1.53	1.42	1.65	4.43E-29	1.25E-39	3.52E-05	B*15:01,B*15:02,B*15:03,B*15:05,B*15:06,B*15:07,B*15:08,B*15:11,B*15:12,B*15:13,B*15:18,B*15:21,B*15:25,B*15:27,B*15:29,B*15:32,B*15:35,B*15:46,B*18:01,B*18:02,B*35:01,B*35:05,B*35:08,B*35:43,B*40:03,B*46:01,B*46:13,B*53:01,B*56:03,B*57:01,B*58:01
	31356368	Other	Y	0.261	0.252	0.269	0.92	0.84	0.99	3.76E-02		2.79E-01	B*07:02,B*07:05,B*07:53,B*08:01,B*15:10,B*27:06,B*27:07,B*35:02,B*40:01,B*40:02,B*40:06,B*40:10,B*40:40,B*40:50,B*41:01,B*41:02,B*48:01,B*48:03,B*48:04,B*51:01,B*51:02,B*51:03,B*51:06,B*51:07,B*52:01,B*55:04,B*55:08,B*78:02,B*81:01
	31356368	Other	L	0.138	0.102	0.173	0.54	0.48	0.61	4.18E-27		2.37E-13	B*13:01,B*13:02,B*15:86,B*45:01,B*45:04,B*49:01,B*50:01,B*54:01,B*55:01,B*55:02,B*55:07,B*55:12,B*55:46,B*56:01,B*56:02,B*56:04,B*59:01
	31356368	Other	F	0.089	0.086	0.092	0.93	0.82	1.06	2.74E-01		1.58E-07	B*14:01,B*14:02,B*15:58,B*35:03,B*37:01,B*38:01,B*38:02,B*38:15,B*39:01,B*39:05,B*39:06,B*39:09,B*39:15,B*67:01
	31356368	Other	D	0.032	0.025	0.038	0.66	0.53	0.82	1.50E-04		2.54E-04	B*15:17,B*27:02,B*27:04,B*27:05,B*44:02,B*44:03,B*47:01
114	31356374	N	Other	0.474	0.428	0.518	0.70	0.65	0.75	1.28E-21	3.17E-42	2.54E-03	B*07:05,B*07:53,B*08:01,B*13:01,B*13:02,B*14:01,B*14:02,B*15:58,B*15:86,B*27:07,B*35:02,B*37:01,B*38:01,B*38:02,B*38:15,B*39:01,B*39:05,B*39:06,B*39:09,B*40:01,B*40:02,B*40:06,B*40:10,B*40:40,B*40:50,B*41:01,B*41:02,B*45:01,B*45:04,B*48:01,B*48:03,B*48:04,B*49:01,B*50:01,B*51:01,B*51:02,B*51:03,B*51:06,B*51:07,B*52:01,B*54:01,B*55:01,B*55:02,B*55:04,B*55:07,B*55:08,B*55:12,B*56:01,B*56:02,B*56:04,B*59:01,B*67:01,B*78:02,B*81:01
	31356374	Other	D	0.491	0.436	0.543	0.65	0.61	0.71	7.51E-29		1.14E-04	B*07:02,B*15:01,B*15:02,B*15:03,B*15:05,B*15:06,B*15:07,B*15:08,B*15:10,B*15:11,B*15:12,B*15:13,B*15:18,B*15:21,B*15:25,B*15:27,B*15:29,B*15:32,B*15:35,B*15:46,B*18:01,B*18:02,B*27:06,B*35:01,B*35:03,B*35:05,B*35:08,B*35:43,B*39:15,B*40:03,B*44:02,B*44:03,B*46:01,B*46:13,B*53:01,B*55:46,B*56:03,B*57:01,B*58:01
	31356374	Other	H	0.017	0.009	0.025	0.36	0.26	0.50	8.30E-10		1.02E-04	B*15:17,B*27:02,B*27:04,B*27:05,B*47:01
113	31356377	H	Y	0.155	0.146	0.163	0.87	0.79	0.96	8.12E-03		2.76E-03	B*14:01,B*14:02,B*15:02,B*15:06,B*15:13,B*15:17,B*15:21,B*15:25,B*27:02,B*27:04,B*27:05,B*27:06,B*37:01,B*44:02,B*44:03,B*45:01,B*45:04,B*47:01,B*49:01,B*50:01
109	31356389	L	F	0.000	0.000	0.001	0.35	0.04	3.36	3.62E-01		2.07E-01	B*35:02
103	31356407	V	L	0.265	0.233	0.295	0.72	0.67	0.79	1.03E-13		2.57E-03	B*13:01,B*13:02,B*15:86,B*35:01,B*35:02,B*35:03,B*35:05,B*35:08,B*45:01,B*45:04,B*49:01,B*50:01,B*53:01,B*54:01,B*55:01,B*55:02,B*55:07,B*55:12,B*55:46,B*56:01,B*56:02,B*58:01,B*59:01
99	31356419	Y	Other	0.013	0.013	0.014	0.91	0.66	1.26	5.80E-01	5.73E-01	5.66E-01	B*07:02,B*07:05,B*07:53,B*08:01,B*13:01,B*13:02,B*14:01,B*14:02,B*15:01,B*15:02,B*15:03,B*15:05,B*15:07,B*15:08,B*15:10,B*15:11,B*15:12,B*15:13,B*15:17,B*15:18,B*15:21,B*15:25,B*15:29,B*15:35,B*15:46,B*15:58,B*15:86,B*18:01,B*18:02,B*27:02,B*27:04,B*27:05,B*27:06,B*27:07,B*35:01,B*35:02,B*35:03,B*35:05,B*35:08,B*35:43,B*38:01,B*38:02,B*38:15,B*39:01,B*39:05,B*39:06,B*39:15,B*40:01,B*40:02,B*40:03,B*40:06,B*40:10,B*40:40,B*40:50,B*41:01,B*41:02,B*44:02,B*44:03,B*45:01,B*45:04,B*46:01,B*46:13,B*48:01,B*48:03,B*48:04,B*49:01,B*50:01,B*51:01,B*51:02,B*51:03,B*51:06,B*51:07,B*52:01,B*53:01,B*54:01,B*55:01,B*55:02,B*55:04,B*55:07,B*55:08,B*55:12,B*55:46,B*56:01,B*56:02,B*56:03,B*56:04,B*57:01,B*58:01,B*59:01,B*67:01,B*78:02,B*81:01
	31356419	Other	S	0.006	0.005	0.007	0.78	0.49	1.26	3.16E-01		7.80E-01	B*15:06,B*15:27,B*47:01
	31356419	Other	F	0.007	0.007	0.007	1.04	0.67	1.63	8.49E-01		6.05E-01	B*15:32,B*37:01,B*39:09
97	31356425	R	Other	0.209	0.173	0.242	0.66	0.60	0.72	6.22E-19	1.30E-19	4.41E-11	B*13:01,B*15:01,B*15:02,B*15:03,B*15:05,B*15:06,B*15:08,B*15:10,B*15:11,B*15:12,B*15:13,B*15:17,B*15:18,B*15:21,B*15:25,B*15:27,B*15:29,B*15:32,B*15:46,B*15:58,B*15:86,B*18:01,B*35:01,B*35:02,B*35:03,B*35:08,B*35:43,B*37:01,B*38:01,B*38:02,B*38:15,B*39:01,B*39:05,B*39:09,B*39:15,B*40:01,B*40:10,B*41:01,B*44:02,B*44:03,B*45:01,B*45:04,B*46:01,B*46:13,B*47:01,B*48:03,B*49:01,B*50:01,B*51:06,B*53:01,B*55:08,B*56:02,B*56:03,B*56:04,B*58:01,B*67:01
	31356425	Other	T	0.135	0.116	0.153	0.73	0.65	0.81	1.01E-08		5.20E-07	B*13:02,B*15:35,B*39:06,B*40:06,B*51:01,B*51:02,B*51:03,B*51:07,B*52:01,B*54:01,B*55:01,B*55:02,B*55:07,B*55:12,B*55:46,B*56:01,B*59:01,B*78:02
	31356425	Other	S	0.050	0.042	0.057	0.72	0.61	0.86	2.17E-04		9.13E-02	B*07:02,B*07:05,B*07:53,B*08:01,B*15:07,B*27:07,B*35:05,B*40:02,B*40:03,B*40:40,B*40:50,B*41:02,B*48:01,B*48:04,B*55:04,B*81:01
	31356425	Other	N	0.021	0.012	0.029	0.43	0.32	0.57	4.88E-09		7.48E-05	B*18:02,B*27:02,B*27:04,B*27:05,B*27:06

	31356425	Other	W	0.000	0.000	0.000	NA	NA	NA	NA		NA	B*14:01,B*14:02
	31356425	Other	V	0.003	0.003	0.003	0.89	0.46	1.74	7.42E-01		2.12E-02	B*57:01
95	31356431	L	Other	0.452	0.418	0.484	0.76	0.71	0.82	1.23E-12	7.92E-13	1.99E-06	B*07:02,B*07:05,B*07:53,B*08:01,B*14:01,B*14:02,B*15:01,B*15:03,B*15:05,B*15:06,B*15:07,B*15:08,B*15:10,B*15:11,B*15:12,B*15:17,B*15:18,B*15:27,B*15:29,B*15:32,B*15:35,B*15:46,B*15:58,B*15:86,B*18:01,B*18:02,B*27:02,B*27:04,B*27:05,B*27:06,B*27:07,B*35:05,B*35:43,B*38:01,B*38:02,B*38:15,B*39:01,B*39:05,B*39:09,B*39:15,B*40:01,B*40:02,B*40:03,B*40:10,B*40:40,B*40:50,B*41:02,B*46:01,B*46:13,B*47:01,B*48:01,B*48:03,B*48:04,B*51:06,B*55:04,B*55:08,B*56:02,B*56:03,B*56:04,B*67:01,B*81:01
	31356431	Other	W	0.140	0.122	0.156	0.75	0.68	0.84	2.76E-07		3.95E-07	B*13:02,B*39:06,B*40:06,B*41:01,B*45:01,B*45:04,B*49:01,B*50:01,B*51:01,B*51:02,B*51:03,B*51:07,B*52:01,B*54:01,B*55:01,B*55:02,B*55:07,B*55:12,B*55:46,B*56:01,B*59:01,B*78:02
	31356431	Other	I	0.312	0.295	0.328	0.86	0.79	0.93	1.33E-04		1.58E-01	B*13:01,B*15:02,B*15:13,B*15:21,B*15:25,B*35:01,B*35:02,B*35:03,B*35:08,B*37:01,B*44:02,B*44:03,B*53:01,B*57:01,B*58:01
94	31356434	T	I	0.307	0.291	0.322	0.86	0.79	0.93	1.99E-04		1.83E-01	B*13:01,B*15:02,B*15:13,B*15:21,B*15:25,B*35:01,B*35:02,B*35:03,B*35:08,B*44:02,B*44:03,B*53:01,B*57:01,B*58:01
92	31356718	S	P									NA	B*38:15
83	31356712	G	R	0.324	0.305	0.342	0.85	0.78	0.91	3.06E-05		3.78E-01	B*13:01,B*13:02,B*15:13,B*15:17,B*27:02,B*27:04,B*27:05,B*27:06,B*27:07,B*37:01,B*38:01,B*38:02,B*38:15,B*44:02,B*44:03,B*47:01,B*49:01,B*51:01,B*51:02,B*51:03,B*51:06,B*51:07,B*52:01,B*53:01,B*57:01,B*58:01,B*59:01
82	31356715	R	L	0.324	0.305	0.342	0.85	0.78	0.91	3.06E-05		3.78E-01	B*13:01,B*13:02,B*15:13,B*15:17,B*27:02,B*27:04,B*27:05,B*27:06,B*27:07,B*37:01,B*38:01,B*38:02,B*38:15,B*44:02,B*44:03,B*47:01,B*49:01,B*51:01,B*51:02,B*51:03,B*51:06,B*51:07,B*52:01,B*53:01,B*57:01,B*58:01,B*59:01
81	31356718	L	A	0.300	0.291	0.309	0.91	0.84	0.99	2.91E-02		5.95E-02	B*13:01,B*13:02,B*15:13,B*15:17,B*27:02,B*38:01,B*38:02,B*38:15,B*44:02,B*44:03,B*49:01,B*51:01,B*51:02,B*51:03,B*51:06,B*51:07,B*52:01,B*53:01,B*57:01,B*58:01,B*59:01
80	31356721	N	Other	0.324	0.305	0.342	0.85	0.78	0.91	3.06E-05	4.35E-10	3.78E-01	B*07:02,B*07:05,B*07:53,B*08:01,B*14:01,B*14:02,B*15:01,B*15:02,B*15:03,B*15:05,B*15:06,B*15:07,B*15:08,B*15:10,B*15:11,B*15:12,B*15:18,B*15:21,B*15:25,B*15:27,B*15:29,B*15:32,B*15:35,B*15:46,B*15:58,B*15:86,B*18:01,B*18:02,B*35:01,B*35:02,B*35:03,B*35:05,B*35:08,B*35:43,B*39:01,B*39:05,B*39:06,B*39:09,B*39:15,B*40:01,B*40:02,B*40:03,B*40:06,B*40:10,B*40:40,B*40:50,B*41:01,B*41:02,B*45:01,B*45:04,B*46:01,B*46:13,B*48:01,B*48:03,B*48:04,B*50:01,B*54:01,B*55:01,B*55:02,B*55:04,B*55:07,B*55:08,B*55:12,B*55:46,B*56:01,B*56:02,B*56:03,B*56:04,B*67:01,B*78:02,B*81:01
	31356721	Other	I	0.156	0.161	0.152	1.08	0.97	1.19	1.46E-01		1.51E-01	B*15:13,B*15:17,B*27:02,B*38:01,B*49:01,B*51:01,B*51:02,B*51:03,B*51:06,B*51:07,B*52:01,B*53:01,B*57:01,B*58:01,B*59:01
	31356721	Other	T	0.167	0.144	0.190	0.72	0.65	0.79	6.73E-11		7.34E-01	B*13:01,B*13:02,B*27:04,B*27:05,B*27:06,B*27:07,B*37:01,B*38:02,B*38:15,B*44:02,B*44:03,B*47:01
77	31356730	S	Other	0.309	0.297	0.320	0.90	0.83	0.97	7.66E-03	7.03E-03	9.22E-02	B*07:02,B*07:05,B*07:53,B*08:01,B*14:01,B*14:02,B*15:01,B*15:02,B*15:03,B*15:05,B*15:06,B*15:07,B*15:08,B*15:10,B*15:11,B*15:12,B*15:18,B*15:21,B*15:25,B*15:27,B*15:29,B*15:32,B*15:35,B*15:46,B*15:58,B*15:86,B*18:01,B*18:02,B*27:04,B*27:06,B*35:01,B*35:02,B*35:03,B*35:05,B*35:08,B*35:43,B*39:01,B*39:05,B*39:06,B*39:09,B*39:15,B*40:01,B*40:02,B*40:03,B*40:06,B*40:10,B*40:40,B*40:50,B*41:01,B*41:02,B*45:01,B*45:04,B*46:01,B*46:13,B*48:01,B*48:03,B*48:04,B*50:01,B*54:01,B*55:01,B*55:02,B*55:04,B*55:07,B*55:08,B*55:46,B*56:01,B*56:02,B*56:03,B*56:04,B*67:01,B*78:02,B*81:01
	31356730	Other	N	0.301	0.291	0.311	0.91	0.84	0.99	2.04E-02		1.55E-01	B*13:01,B*13:02,B*15:13,B*15:17,B*27:02,B*38:01,B*38:02,B*38:15,B*44:02,B*44:03,B*49:01,B*51:01,B*51:02,B*51:03,B*51:06,B*51:07,B*52:01,B*53:01,B*55:12,B*57:01,B*58:01,B*59:01
	31356730	Other	D	0.008	0.006	0.009	0.65	0.42	1.01	5.50E-02		5.95E-02	B*27:05,B*27:07,B*37:01,B*47:01
76	31356733	E	V	0.193	0.247	0.143	1.93	1.76	2.13	1.20E-41		6.83E-04	B*46:01,B*46:13
74	31356739	Y	D	0.294	0.307	0.281	1.13	1.04	1.22	3.08E-03		2.64E-02	B*07:02,B*07:05,B*07:53,B*08:01,B*14:01,B*14:02,B*27:02,B*27:04,B*27:05,B*27:06,B*27:07,B*39:01,B*39:06,B*39:09,B*39:15,B*40:50,B*46:01,B*46:13,B*54:01,B*55:01,B*55:02,B*55:04,B*55:07,B*55:08,B*55:12,B*55:46,B*56:01,B*56:02,B*56:03,B*56:04,B*67:01,B*81:01
71	31356748	T	A	0.374	0.401	0.349	1.25	1.16	1.34	1.14E-08		2.81E-01	B*07:02,B*07:05,B*07:53,B*15:17,B*27:02,B*27:04,B*27:05,B*27:06,B*27:07,B*46:01,B*46:13,B*54:01,B*55:01,B*55:02,B*55:04,B*55:07,B*55:08,B*55:12,B*55:46,B*56:01,B*56:02,B*56:03,B*56:04,B*57:01,B*58:01,B*67:01,B*81:01
70	31356751	N	Other	0.374	0.401	0.349	1.25	1.16	1.34	1.14E-08	3.90E-21	2.81E-01	B*08:01,B*13:01,B*13:02,B*14:01,B*14:02,B*15:01,B*15:02,B*15:03,B*15:05,B*15:06,B*15:07,B*15:08,B*15:10,B*15:11,B*15:12,B*15:13,B*15:18,B*15:21,B*15:25,B*15:27,B*15:29,B*15:32,B*15:35,B*15:46,B*15:58,B*15:86,B*18:01,B*18:02,B*35:01,B*35:02,B*35:03,B*35:05,B*35:08,B*35:43,B*37:01,B*38:01,B*38:02,B*38:15,B*39:01,B*39:05,B*39:06,B*39:09,B*39:15,B*40:01,B*40:02,B*40:03,B*40:06,B*40:10,B*40:40,B*40:50,B*41:01,B*41:02,B*44:02,B*44:03,B*45:01,B*45:04,B*47:01,B*48:01,B*48:03,B*48:04,B*49:01,B*50:01,B*51:01,B*51:02,B*51:03,B*51:06,B*51:07,B*52:01,B*53:01,B*59:01,B*78:02
	31356751	Other	Q	0.262	0.292	0.234	1.35	1.24	1.47	2.55E-12		7.21E-01	B*07:02,B*07:05,B*07:53,B*46:01,B*46:13,B*54:01,B*55:01,B*55:02,B*55:04,B*55:07,B*55:08,B*55:12,B*55:46,B*56:01,B*56:02,B*56:03,B*56:04,B*67:01,B*81:01
	31356751	Other	K	0.018	0.009	0.026	0.35	0.26	0.48	1.58E-10		5.12E-05	B*27:02,B*27:04,B*27:05,B*27:06,B*27:07
	31356751	Other	S	0.094	0.100	0.089	1.14	1.00	1.29	4.69E-02		1.41E-03	B*15:17,B*57:01,B*58:01
69	31356754	T	Other	0.374	0.401	0.349	1.25	1.16	1.34	1.14E-08	2.24E-47	2.81E-01	B*08:01,B*13:01,B*13:02,B*14:01,B*14:02,B*15:01,B*15:02,B*15:03,B*15:05,B*15:06,B*15:07,B*15:08,B*15:10,B*15:11,B*15:12,B*15:13,B*15:18,B*15:21,B*15:25,B*15:27,B*15:29,B*15:32,B*15:35,B*15:46,B*15:58,B*15:86,B*18:01,B*18:02,B*35:01,B*35:02,B*35:03,B*35:05,B*35:08,B*35:43,B*37:01,B*38:01,B*38:02,B*38:15,B*39:01,B*39:05,B*39:06,B*39:09,B*39:15,B*40:01,B*40:02,B*40:03,B*40:06,B*40:10,B*40:40,B*40:50,B*41:01,B*41:02,B*44:02,B*44:03,B*45:01,B*45:04,B*47:01,B*48:01,B*48:03,B*48:04,B*49:01,B*50:01,B*51:01,B*51:02,B*51:03,B*51:06,B*51:07,B*52:01,B*53:01,B*59:01,B*78:02
	31356754	Other	A	0.181	0.154	0.206	0.70	0.64	0.78	1.12E-12		7.65E-02	B*07:02,B*07:05,B*07:53,B*15:17,B*27:02,B*27:04,B*27:05,B*27:06,B*27:07,B*54:01,B*55:01,B*55:02,B*55:04,B*55:07,B*55:08,B*55:12,B*55:46,B*56:01,B*56:02,B*56:03,B*56:04,B*57:01,B*58:01,B*67:01,B*81:01
	31356754	Other	R	0.193	0.247	0.143	1.93	1.76	2.13	1.20E-41		6.29E-04	B*46:01,B*46:13

67	31356760	Other	S	0.457	0.439	0.474	0.86	0.80	0.93	1.11E-04	6.13E-13	3.09E-04	B*13:01,B*13:02,B*15:01,B*15:02,B*15:03,B*15:05,B*15:06,B*15:07,B*15:12,B*15:13,B*15:25,B*15:27,B*15:32,B*15:35,B*15:46,B*15:58,B*15:86,B*18:01,B*18:02,B*37:01,B*40:01,B*40:02,B*40:03,B*40:06,B*40:10,B*40:40,B*40:50,B*41:01,B*41:02,B*44:02,B*44:03,B*45:01,B*45:04,B*47:01,B*48:01,B*48:03,B*48:04,B*49:01,B*50:01,B*51:07,B*52:01
	31356760	Other	Y	0.265	0.294	0.238	1.32	1.22	1.44	4.46E-11		7.55E-01	B*07:02,B*07:05,B*07:53,B*15:11,B*46:01,B*46:13,B*54:01,B*55:01,B*55:02,B*55:04,B*55:07,B*55:08,B*55:12,B*55:46,B*56:01,B*56:02,B*56:03,B*56:04,B*67:01,B*81:01
	31356760	Other	C	0.094	0.082	0.106	0.75	0.66	0.85	1.12E-05		8.61E-04	B*14:01,B*14:02,B*15:10,B*15:18,B*15:21,B*27:02,B*27:04,B*27:05,B*27:06,B*27:07,B*38:01,B*38:02,B*38:15,B*39:01,B*39:05,B*39:06,B*39:09,B*39:15
	31356760	Other	F	0.089	0.086	0.092	0.93	0.82	1.06	2.85E-01		2.18E-01	B*08:01,B*15:08,B*15:29,B*35:01,B*35:02,B*35:03,B*35:05,B*35:08,B*35:43,B*51:01,B*51:02,B*51:03,B*51:06,B*53:01,B*59:01,B*78:02
	31356760	Other	M	0.094	0.100	0.089	1.14	1.00	1.29	4.69E-02		1.41E-03	B*15:17,B*57:01,B*58:01
66	31356763	I	Other	0.288	0.347	0.232	1.75	1.61	1.90	8.41E-40	1.21E-47	2.00E-07	B*07:02,B*07:05,B*07:53,B*08:01,B*13:01,B*13:02,B*14:01,B*14:02,B*15:01,B*15:02,B*15:03,B*15:05,B*15:06,B*15:07,B*15:08,B*15:10,B*15:11,B*15:12,B*15:13,B*15:18,B*15:21,B*15:25,B*15:27,B*15:29,B*15:32,B*15:35,B*15:46,B*15:58,B*15:86,B*18:01,B*18:02,B*27:02,B*27:04,B*27:05,B*27:06,B*27:07,B*35:01,B*35:02,B*35:03,B*35:05,B*35:08,B*35:43,B*37:01,B*38:01,B*38:02,B*38:15,B*39:01,B*39:05,B*39:06,B*39:09,B*39:15,B*40:01,B*40:02,B*40:03,B*40:06,B*40:10,B*40:40,B*40:50,B*41:01,B*41:02,B*44:02,B*44:03,B*45:01,B*45:04,B*47:01,B*48:01,B*48:03,B*48:04,B*49:01,B*50:01,B*51:01,B*51:02,B*51:03,B*51:06,B*51:07,B*52:01,B*53:01,B*54:01,B*55:01,B*55:02,B*55:04,B*55:07,B*55:08,B*55:12,B*55:46,B*56:01,B*56:02,B*56:03,B*56:04,B*59:01,B*67:01,B*78:02,B*81:01
	31356763	Other	N	0.094	0.100	0.089	1.14	1.00	1.29	4.69E-02		1.41E-03	B*15:17,B*57:01,B*58:01
	31356763	Other	K	0.193	0.247	0.143	1.93	1.76	2.13	1.20E-41		6.29E-04	B*46:01,B*46:13
65	31356766	Q	R	0.094	0.100	0.089	1.14	1.00	1.29	4.69E-02		1.41E-03	B*15:17,B*57:01,B*58:01
63	31356772	N	E	0.344	0.314	0.372	0.78	0.72	0.84	1.96E-10		3.58E-01	B*13:01,B*13:02,B*15:01,B*15:03,B*15:05,B*15:06,B*15:07,B*15:12,B*15:17,B*15:25,B*15:27,B*15:32,B*15:35,B*15:46,B*15:58,B*15:86,B*27:02,B*27:04,B*27:05,B*27:06,B*27:07,B*37:01,B*40:01,B*40:02,B*40:03,B*40:06,B*40:10,B*40:40,B*40:50,B*41:01,B*41:02,B*44:02,B*44:03,B*45:01,B*45:04,B*46:01,B*46:13,B*47:01,B*48:01,B*48:03,B*48:04,B*49:01,B*50:01,B*52:01,B*57:01,B*58:01
62	31356775	R	G	0.094	0.100	0.089	1.14	1.00	1.30	4.34E-02		1.41E-03	B*57:01,B*58:01
52	31356805	I	V	0.026	0.026	0.026	1.02	0.81	1.29	8.35E-01		1.35E-01	B*54:01
46	31356823	E	A	0.425	0.452	0.399	1.23	1.15	1.33	2.26E-08		5.72E-02	B*13:01,B*13:02,B*15:01,B*15:02,B*15:05,B*15:06,B*15:07,B*15:08,B*15:11,B*15:12,B*15:13,B*15:17,B*15:21,B*15:25,B*15:27,B*15:32,B*15:35,B*15:58,B*15:86,B*46:01,B*46:13,B*57:01
45	31356826	Other	E	0.161	0.128	0.193	0.61	0.55	0.68	1.25E-20	2.11E-19	8.32E-02	B*07:02,B*07:05,B*07:53,B*08:01,B*14:01,B*14:02,B*15:03,B*15:10,B*15:18,B*15:29,B*27:02,B*27:04,B*27:05,B*27:06,B*27:07,B*38:01,B*38:02,B*38:15,B*39:01,B*39:05,B*39:06,B*39:09,B*39:15,B*48:01,B*48:03,B*48:04,B*55:01,B*55:02,B*55:04,B*55:08,B*55:12,B*55:46,B*56:01,B*56:02,B*56:03,B*56:04,B*59:01,B*67:01,B*81:01
	31356826	Other	M	0.425	0.452	0.399	1.23	1.15	1.33	2.26E-08		5.72E-02	B*13:01,B*13:02,B*15:01,B*15:02,B*15:05,B*15:06,B*15:07,B*15:08,B*15:11,B*15:12,B*15:13,B*15:17,B*15:21,B*15:25,B*15:27,B*15:32,B*15:35,B*15:58,B*15:86,B*46:01,B*46:13,B*57:01
	31356826	Other	T	0.196	0.201	0.191	1.07	0.98	1.18	1.44E-01		3.37E-02	B*18:01,B*18:02,B*35:01,B*35:02,B*35:03,B*35:05,B*35:08,B*35:43,B*37:01,B*51:01,B*51:02,B*51:03,B*51:06,B*51:07,B*52:01,B*53:01,B*58:01,B*78:02
	31356826	Other	K	0.192	0.193	0.192	1.01	0.92	1.10	9.12E-01		1.35E-01	B*15:46,B*40:01,B*40:02,B*40:03,B*40:06,B*40:10,B*40:40,B*40:50,B*41:01,B*41:02,B*44:02,B*44:03,B*45:01,B*45:04,B*47:01,B*49:01,B*50:01
	31356826	Other	G	0.026	0.026	0.026	1.01	0.80	1.27	9.26E-01		1.85E-01	B*54:01,B*55:07
41	31356838	A	T	0.268	0.246	0.288	0.81	0.74	0.88	4.25E-07		1.92E-02	B*13:01,B*13:02,B*15:46,B*40:01,B*40:02,B*40:03,B*40:06,B*40:10,B*40:40,B*40:50,B*41:01,B*41:02,B*44:02,B*44:03,B*45:01,B*45:04,B*47:01,B*49:01,B*50:01
32	31356937	Q	L									8.27E-01	B*27:02,B*27:04,B*27:05,B*27:06,B*27:07,B*40:01,B*40:02,B*40:03,B*40:06,B*40:40,B*40:50,B*41:01,B*41:02,B*44:02,B*44:03,B*45:01,B*45:04,B*47:01,B*49:01,B*50:01
30	31356934	D	G									5.73E-01	B*18:01,B*18:02
24	31356865	A	Other	0.406	0.367	0.443	0.73	0.68	0.79	2.68E-16	9.80E-16	5.92E-01	B*15:01,B*15:02,B*15:05,B*15:06,B*15:07,B*15:08,B*15:11,B*15:12,B*15:13,B*15:17,B*15:21,B*15:25,B*15:27,B*15:32,B*15:35,B*15:46,B*15:58,B*15:86,B*35:01,B*35:02,B*35:03,B*35:05,B*35:08,B*35:43,B*40:10,B*46:01,B*46:13,B*51:01,B*51:02,B*51:03,B*51:06,B*51:07,B*52:01,B*53:01,B*54:01,B*55:01,B*55:02,B*55:04,B*55:07,B*55:08,B*55:12,B*55:46,B*56:01,B*56:02,B*56:03,B*56:04,B*57:01,B*58:01,B*59:01,B*78:02
	31356865	Other	S	0.122	0.112	0.131	0.84	0.75	0.94	2.77E-03		6.12E-04	B*07:02,B*07:05,B*07:53,B*08:01,B*14:01,B*14:02,B*15:03,B*15:10,B*15:18,B*15:29,B*18:01,B*18:02,B*37:01,B*38:01,B*38:02,B*38:15,B*39:01,B*39:05,B*39:06,B*39:09,B*39:15,B*48:01,B*48:03,B*48:04,B*67:01,B*81:01
	31356865	Other	T	0.285	0.255	0.313	0.75	0.69	0.82	9.66E-12		8.96E-04	B*13:01,B*13:02,B*27:02,B*27:04,B*27:05,B*27:06,B*27:07,B*40:01,B*40:02,B*40:03,B*40:06,B*40:40,B*40:50,B*41:01,B*41:02,B*44:02,B*44:03,B*45:01,B*45:04,B*47:01,B*49:01,B*50:01
12	31356925	M	V	0.160	0.141	0.177	0.77	0.69	0.85	3.50E-07		3.48E-03	B*07:02,B*07:05,B*07:53,B*14:01,B*14:02,B*18:01,B*18:02,B*27:02,B*27:04,B*27:05,B*27:06,B*27:07,B*37:01,B*38:01,B*38:02,B*38:15,B*39:01,B*39:05,B*39:06,B*39:09,B*39:15,B*40:02,B*40:03,B*40:06,B*40:40,B*40:50,B*48:01,B*48:03,B*48:04,B*67:01,B*81:01
11	31356928	A	S	0.160	0.141	0.177	0.77	0.69	0.85	3.97E-07		3.48E-03	B*07:02,B*07:05,B*07:53,B*14:01,B*18:01,B*18:02,B*27:02,B*27:04,B*27:05,B*27:06,B*27:07,B*37:01,B*38:01,B*38:02,B*38:15,B*39:01,B*39:05,B*39:06,B*39:09,B*39:15,B*40:02,B*40:03,B*40:06,B*40:40,B*40:50,B*48:01,B*48:03,B*48:04,B*67:01,B*81:01
9	31356934	Y	Other	0.207	0.197	0.217	0.89	0.81	0.97	1.05E-02	9.57E-03	9.62E-01	B*07:02,B*07:05,B*07:53,B*13:01,B*13:02,B*14:01,B*14:02,B*15:01,B*15:02,B*15:03,B*15:05,B*15:06,B*15:07,B*15:08,B*15:10,B*15:11,B*15:12,B*15:13,B*15:17,B*15:18,B*15:21,B*15:25,B*15:27,B*15:29,B*15:32,B*15:35,B*15:46,B*15:58,B*15:86,B*35:01,B*35:02,B*35:03,B*35:05,B*35:08,B*35:43,B*38:01,B*38:02,B*38:15,B*39:01,B*39:05,B*39:06,B*39:09,B*39:15,B*40:10,B*40:40,B*44:02,B*44:03,B*46:01,B*46:13,B*47:01,B*48:01,B*48:03,B*48:04,B*51:01,B*51:02,B*51:03,B*51:06,B*51:07,B*52:01,B*53:01,B*54:01,B*55:01,B*55:02,B*55:04,B*55:07,B*55:08,B*55:12,B*55:46,B*56:01,B*56:02,B*56:03,B*56:04,B*57:01,B*58:01,B*59:01,B*67:01,B*78:02,B*81:01

		31356934	Other	H	0.205	0.196	0.214	0.90	0.82	0.98	1.98E-02		7.78E-01	B*18:01,B*18:02,B*27:02,B*27:04,B*27:05,B*27:06,B*27:07,B*37:01,B*40:01,B*40:02,B*40:03,B*40:06,B*40:50,B*41:01,B*41:02,B*45:01,B*45:04,B*49:01,B*50:01
		31356934	Other	D	0.002	0.001	0.003	0.38	0.15	0.96	4.06E-02		9.41E-03	B*08:01
	-8	31357110	L	V	0.334	0.308	0.358	0.80	0.74	0.87	2.79E-08		3.10E-02	B*13:01,B*13:02,B*18:01,B*18:02,B*27:02,B*27:04,B*27:05,B*27:06,B*27:07,B*35:01,B*35:02,B*35:03,B*35:05,B*35:08,B*35:43,B*37:01,B*40:02,B*40:03,B*40:06,B*40:50,B*44:02,B*44:03,B*47:01,B*51:01,B*51:02,B*51:03,B*51:06,B*51:07,B*52:01,B*53:01,B*57:01,B*58:01,B*78:02,B*81:01
	-10	31357116	G	A	0.257	0.248	0.265	0.92	0.84	1.00	4.04E-02		4.64E-04	B*07:02,B*07:05,B*07:53,B*08:01,B*14:01,B*14:02,B*38:01,B*38:02,B*39:01,B*39:05,B*39:06,B*39:09,B*39:15,B*40:01,B*41:01,B*41:02,B*45:01,B*45:04,B*48:01,B*48:03,B*48:04,B*49:01,B*50:01,B*67:01
	-11	31357119	S	W	0.392	0.352	0.431	0.72	0.67	0.77	1.61E-17		4.85E-06	B*13:01,B*13:02,B*18:01,B*18:02,B*27:02,B*27:04,B*27:05,B*27:06,B*27:07,B*35:01,B*35:02,B*35:03,B*35:05,B*35:08,B*35:43,B*37:01,B*40:02,B*40:03,B*40:06,B*40:10,B*40:50,B*44:02,B*44:03,B*47:01,B*51:01,B*51:02,B*51:03,B*51:06,B*51:07,B*52:01,B*53:01,B*54:01,B*55:01,B*55:02,B*55:04,B*55:12,B*56:01,B*56:02,B*56:03,B*56:04,B*57:01,B*58:01,B*59:01,B*78:02,B*81:01
	-16	31357134	V	L	0.204	0.158	0.246	0.58	0.52	0.63	2.54E-30		1.70E-13	B*13:01,B*13:02,B*18:01,B*18:02,B*27:02,B*27:04,B*27:05,B*27:06,B*27:07,B*37:01,B*40:02,B*40:03,B*40:06,B*40:10,B*40:50,B*44:02,B*44:03,B*47:01,B*54:01,B*55:01,B*55:02,B*55:04,B*55:12,B*56:01,B*56:02,B*56:03,B*56:04,B*59:01
	-21	31357149	T	M	0.107	0.097	0.116	0.82	0.73	0.93	1.49E-03		3.72E-04	B*07:02,B*07:05,B*07:53,B*08:01,B*14:01,B*14:02,B*38:01,B*38:02,B*39:01,B*39:05,B*39:06,B*39:09,B*39:15,B*48:01,B*48:03,B*48:04,B*67:01,B*81:01
	-23	31357155	R	L	0.107	0.097	0.116	0.82	0.73	0.93	1.49E-03		3.72E-04	B*07:02,B*07:05,B*07:53,B*08:01,B*14:01,B*14:02,B*38:01,B*38:02,B*39:01,B*39:05,B*39:06,B*39:09,B*39:15,B*48:01,B*48:03,B*48:04,B*67:01,B*81:01

Supplementary Table 7: Multivariate logistic regression analysis for significant variants and previous reported signals

	Variant 2 \ Variant 1	rs29230	rs29232	rs3129055	rs2860580	A*02:06	A*02:03	A*11:01	A*33:03	A*02:07	Q62	Q70	R97	I97	C99	R114	L276	rs2517713	rs9260734	rs3869062	rs5009448	rs16896923
		HLA-A																				
unadjusted $-\log_{10}P$		22.25	26.03	21.67	36.79	0.11	0.89	32.52	1.28	44.96	42.64	43.20	34.70	33.83	44.96	35.07	34.09	34.53	33.53	30.46	27.91	18.83
HLA-A	rs29230	NA	13.56	11.39	17.75	0.31	0.14	13.27	0.51	34.75	23.15	24.00	21.30	15.39	34.75	15.74	15.61	15.95	17.71	15.14	13.66	9.67
	rs29232	9.82	NA	2.25	16.66	2.05	5.09	15.04	1.13	27.24	21.99	21.83	32.62	14.12	27.24	15.39	14.34	14.67	15.21	13.10	11.24	6.80
	rs3129055	12.13	6.97	NA	21.96	1.24	3.07	19.44	5.68	26.84	27.45	27.39	26.41	19.33	26.84	20.42	19.56	19.94	19.48	17.49	13.72	9.82
	rs2860580	2.47	5.15	6.14	NA	1.09	0.58	2.58	0.52	26.96	7.29	7.56	11.14	1.07	26.96	0.73	0.80	0.77	2.26	1.38	2.20	0.07
	A*02:06	22.33	27.44	22.41	37.40	1.38	0.91	32.86	1.30	45.36	43.37	43.99	37.05	34.34	45.36	35.61	34.61	35.06	33.93	30.77	28.29	18.93
	A*02:03	21.79	29.69	23.53	36.59	0.15	NA	32.11	1.45	47.21	42.58	43.16	36.31	33.55	47.21	34.81	33.81	34.27	33.14	30.03	27.58	18.36
	A*11:01	2.23	7.94	7.99	7.03	0.72	0.20	NA	0.08	30.95	11.92	12.45	15.10	4.65	30.95	5.39	4.79	5.17	6.36	3.68	4.72	1.46
	A*33:03	21.68	25.91	25.64	36.27	0.17	1.07	31.79	NA	48.04	42.28	42.87	40.94	33.23	48.04	34.47	33.50	33.95	32.83	29.72	27.20	18.10
	A*02:07	11.32	6.87	1.55	18.04	0.81	3.66	17.92	4.93	NA	22.38	22.50	6.56	16.21	NA	17.09	16.41	16.70	17.44	15.79	12.23	8.81
	Q62	1.69	4.29	5.54	0.80	1.25	0.77	1.09	0.73	25.25	NA	3.81	8.27	6.63	25.25	0.40	6.12	4.72	0.64	0.40	0.96	0.30
	Q70	2.00	3.48	4.85	0.05	1.33	0.79	1.01	0.77	24.79	3.18	NA	8.73	0.48	24.79	0.10	0.39	0.33	0.72	0.31	0.62	0.36
	R97	8.63	23.93	13.13	13.55	3.20	2.92	13.17	8.29	18.40	16.96	17.98	NA	11.09	18.40	11.99	11.28	11.69	12.99	11.42	12.91	5.58
	I97	3.24	5.72	6.58	4.06	0.96	0.45	3.46	0.41	28.16	13.19	10.71	11.81	NA	28.16	2.25	0.80	1.28	3.54	2.18	2.75	0.24
	C99	11.32	6.87	1.55	18.04	0.81	3.66	17.92	4.93	NA	22.38	22.50	6.56	16.21	NA	17.09	16.41	16.70	17.44	15.79	12.23	8.81
	R114	2.22	5.69	6.38	2.80	1.01	0.49	2.80	0.40	27.76	8.83	9.31	11.38	0.68	27.76	NA	0.82	1.11	3.80	1.59	1.58	0.08
	L276	3.18	5.67	6.54	3.65	0.97	0.46	3.30	0.41	28.09	12.74	10.41	11.72	0.47	28.09	2.06	NA	0.83	3.43	2.11	2.69	0.22
	rs2517713	3.06	5.54	6.46	3.18	0.98	0.48	3.19	0.43	27.93	11.84	9.95	11.66	0.57	27.93	1.80	0.17	NA	3.13	1.86	2.57	0.18
	rs9260734	5.93	7.11	6.99	5.75	0.80	0.25	5.38	0.18	29.55	10.65	11.29	13.94	3.74	29.55	5.38	3.92	4.11	NA	0.02	1.11	1.02
	rs3869062	6.55	8.22	8.21	8.21	0.67	0.15	6.03	0.05	30.99	13.61	14.12	15.58	5.76	30.99	6.57	5.98	6.22	3.82	NA	1.63	1.09
	rs5009448	7.84	9.14	7.11	11.78	0.76	0.39	9.87	0.21	30.21	16.89	17.18	19.80	9.17	30.21	9.44	9.39	9.74	7.50	4.66	NA	0.69
	rs16896923	13.18	14.08	12.60	19.17	0.34	0.12	16.06	0.22	35.68	25.18	25.77	21.79	16.22	35.68	17.42	16.48	16.90	16.31	13.21	10.42	NA
TRIM31	rs2517664	21.37	23.45	21.05	35.29	0.03	0.45	37.28	0.71	40.43	36.08	37.69	28.68	32.39	40.43	36.10	32.64	33.12	31.21	35.76	29.27	22.55
TRIM39	rs117495548	23.01	29.09	25.30	37.29	0.02	0.57	32.58	1.08	42.09	39.99	43.68	32.74	34.90	42.09	36.26	35.16	35.38	33.75	30.65	29.09	19.00
Top	rs9391681	11.88	7.93	2.23	19.20	0.79	3.42	19.10	5.02	2.07	23.72	23.77	7.74	17.43	2.07	18.39	17.64	17.88	18.45	16.83	13.38	9.46
HLA-C	C*07:02	22.07	25.51	21.19	36.66	0.08	1.42	32.36	1.11	44.62	42.54	43.09	35.24	33.74	44.62	34.95	34.00	34.41	33.13	30.01	27.49	18.83
	C*03:04	20.83	23.14	20.51	33.29	0.01	0.76	28.17	0.68	41.16	38.96	39.68	31.66	30.26	41.16	31.20	30.52	30.99	28.87	25.45	23.55	14.45
	C*03:02	21.52	25.30	24.25	35.98	0.17	1.07	31.75	0.30	47.05	41.86	42.44	38.78	33.01	47.05	34.25	33.27	33.71	32.73	29.70	27.09	18.19
	C*12:03	22.90	26.03	20.89	36.62	0.09	0.97	33.16	1.60	43.97	42.50	42.97	34.02	33.67	43.97	34.81	33.93	34.38	34.18	31.08	27.27	19.28
	C*12:02	22.65	25.04	20.71	35.66	0.16	0.78	33.68	1.11	44.05	41.45	42.09	34.33	32.83	44.05	34.03	33.12	33.53	32.17	29.32	26.63	17.81
	C*01:02	16.09	15.75	8.49	26.37	0.40	1.79	25.41	4.27	20.51	31.72	31.62	18.35	24.19	20.51	25.35	24.43	24.72	25.50	23.55	20.23	13.71
	M248	16.21	15.86	8.61	26.33	0.42	1.79	25.31	4.26	20.96	31.65	31.60	18.37	24.14	20.96	25.24	24.38	24.67	25.47	23.45	20.13	13.57
	W156	22.03	24.84	20.23	33.53	0.10	0.75	35.56	1.24	42.12	39.28	41.35	32.28	30.73	42.12	33.28	31.00	31.44	31.84	31.33	27.19	19.24
	E152	22.78	27.93	22.45	37.41	0.10	0.88	33.48	1.32	47.99	43.31	43.99	34.76	34.41	47.99	35.71	34.68	35.15	33.71	30.77	28.10	18.89
	C99	16.21	15.86	8.61	26.33	0.42	1.79	25.31	4.26	20.96	31.65	31.60	18.37	24.14	20.96	25.24	24.38	24.67	25.47	23.45	20.13	13.57
	L95	19.95	22.91	21.06	33.97	0.05	0.50	28.18	0.38	38.57	39.33	39.14	30.89	31.12	38.57	30.70	31.38	31.82	28.76	25.19	22.38	14.28
	I95	19.45	22.02	20.24	33.37	0.04	0.51	27.74	0.37	38.38	38.72	38.50	30.79	30.57	38.38	30.10	30.82	31.25	28.24	24.66	21.87	13.98
	N77	20.23	24.91	22.03	33.87	0.03	0.81	32.88	0.71	41.35	39.82	39.80	32.03	31.39	41.35	33.10	31.64	32.05	31.91	30.70	26.56	18.60
	F9	16.21	15.86	8.61	26.33	0.42	1.79	25.31	4.26	20.96	31.65	31.60	18.37	24.14	20.96	25.24	24.38	24.67	25.47	23.45	20.13	13.57
	C6	16.21	15.86	8.61	26.33	0.42	1.79	25.31	4.26	20.96	31.65	31.60	18.37	24.14	20.96	25.24	24.38	24.67	25.47	23.45	20.13	13.57
HLA-B	B*58:01	21.48	25.17	24.25	35.89	0.18	1.07	31.69	0.26	46.95	41.76	42.33	38.69	32.93	46.95	34.17	33.19	33.63	32.67	29.65	27.03	18.15
	B*13:01	19.98	22.10	19.62	31.87	0.08	0.74	26.96	0.69	41.60	37.65	38.24	32.29	28.91	41.60	30.06	29.18	29.60	27.62	24.28	22.69	13.33
	B*46:01	15.08	12.95	5.89	23.17	0.48	2.23	22.77	4.69	11.93	28.12	28.07	13.56	21.19	11.93	22.21	21.42	21.69	22.61	20.79	17.53	11.93
	B*55:02	22.94	24.13	20.44	36.32	0.23	1.16	31.96	1.04	42.15	41.92	42.68	34.16	33.24	42.15	34.66	33.50	33.97	31.82	28.81	25.17	17.76
	B*38:02	21.64	28.90	23.47	36.03	0.15	0.13	31.84	1.45	47.24	41.69	42.49	33.38	33.01	47.24	34.25	33.26	33.72	32.92	29.98	27.24	18.65
	B*39:01	21.69	25.14	20.43	35.90	0.08	0.84	31.79	1.16	42.98	41.40	42.30	33.98	32.85	42.98	34.21	33.17	33.57	32.19	29.23	26.10	18.08
	W156	16.96	15.68	8.25	26.11	0.44	1.98	25.09	4.52	18.27	31.10	31.37	17.22	23.72	18.27	24.89	23.97	24.36	25.47	23.58	20.65	14.25
	L145	20.50	21.03	18.72	29.97	0.05	0.66	27.80	0.63	40.96	35.57	36.37	31.06	27.04	40.96	31.04	27.29	27.72	25.92	25.27	23.85	14.06
	L116	20.07	20.78	20.01	29.70	0.03	0.79	27.79	0.30	37.81	34.89	36.87	29.59	26.63	37.81	30.67	26.87	27.30	25.19	24.90	23.01	14.39
	D114	19.25	14.95	16.59	30.35	0.29	3.48	29.69	0.54	29.58	35.34	37.31	32.02	27.67	29.58	29.17	27.95	28.38	24.71	23.20	19.36	14.81
	R97	22.53	25.23	22.70	34.28	0.11	0.33	33.89	0.33	37.86	39.61	38.48	30.01	32.04	37.86	33.94	32.30	32.65	30.00	29.30	24.95	18.20
	R69	15.08	12.93	5.86	23.15	0.49	2.24	22.77	4.70	11.85	28.10	28.05	13.52	21.18	11.85	22.20	21.41	21.68	22.60	20.79	17.52	11.91
	K66	15.08	12.93	5.86	23.15	0.49	2.24	22.77	4.70	11.85	28.10	28.05	13.52	21.18	11.85	22.20	21.41	21.68	22.60	20.79	17.52	11.91
	W-11	22.66	30.69	17.09	35.26	0.05	0.17	33.54	7.37	34.89	40.61	42.20	24.95	32.50	34.89	35.22	32.78	33.22	30.59	28.93	26.73	17.10
	L-16	18.28	22.06	19.22	28.60	0.03	0.55	28.57	0.72	35.22	33.98	36.40	27.51	25.84	35.22	29.65	26.12	26.48	24.55	23.59	21.80	13.33
	rs2894207	19.10	22.31	19.41	29.54	0.17	0.55	30.02	0.68	37.49	34.90	36.47	29.01	26.96	37.49	29.09	27.21	27.64	27.43	27.37	22.62	15.55
	rs9265975	18.75	22.08	19.49	32.28	0.10	0.46	27.65	0.39	39.70	37.42	37.17	32.02	29.83	39.70	29.20	30.10	30.50	27.59	24.64	20.72	13.44
	rs2523589	21.3	15.2	15.8	29.9	0.37	4.06	30.9	0.23	30.9	35.7	36.2	33.2	27.4	30.9	29.8	27.7	28	26.2	24.9	20.6	14
HLA-DQA1	rs9461780	21.9	23.7	20.7	35.8	0.37	0.68	31.2	0.88	42.6	41.3	41.9	34.5	32.8	42.6	33.6	33	33.5	31.9	28.4	26.2	17.8

	Variant 2 \ Variant 1	rs2517664	rs117495548	rs9391681	C*07:02	C*03:04	C*03:02	C*12:03	C*12:02	C*01:02	M248	W156	E152	C99	L95	I95	N77	F9	C6
		TRIM31	TRIM39	Top	HLA-C														
unadjusted $-\log_{10}P$		20.20	12.34	45.82	0.98	12.92	1.36	2.47	3.11	29.49	28.30	6.05	0.08	28.30	20.98	22.58	7.07	28.30	28.30
HLA-A	rs29230	19.53	13.03	36.16	0.73	11.48	0.34	3.18	3.54	23.39	22.29	5.82	0.98	22.29	18.69	19.82	4.93	22.29	22.29
	rs29232	18.17	14.98	29.12	0.16	10.00	0.34	2.47	2.02	19.38	18.25	4.80	2.64	18.25	17.89	18.63	5.89	18.25	18.25
	rs3129055	19.74	15.43	28.29	0.26	11.77	4.26	1.60	2.06	16.69	15.57	4.55	1.28	15.57	20.38	21.19	7.44	15.57	15.57
	rs2860580	19.07	12.87	28.97	0.79	9.26	0.03	2.28	1.82	18.91	17.63	2.46	1.13	17.63	18.08	19.09	3.87	17.63	17.63
	A*02:06	20.19	12.33	46.21	0.97	12.90	1.38	2.47	3.13	29.63	28.44	6.05	0.07	28.44	20.97	22.56	7.05	28.44	28.44
	A*02:03	19.96	12.16	47.87	1.49	12.82	1.52	2.54	3.02	30.21	29.02	5.94	0.02	29.02	20.71	22.31	7.00	29.02	29.02
	A*11:01	24.18	12.46	32.97	0.75	8.37	0.18	3.19	4.40	22.26	20.94	9.30	1.54	20.94	16.53	17.71	7.46	20.94	20.94
	A*33:03	19.86	12.21	48.97	0.80	12.46	0.41	2.76	2.97	32.11	30.91	6.02	0.19	30.91	20.34	21.94	6.61	30.91	30.91
	A*02:07	16.38	9.78	3.12	0.41	8.82	3.86	1.28	2.05	3.35	2.46	2.81	4.02	2.46	14.18	15.64	2.95	2.46	2.46
	Q62	14.77	10.05	27.46	0.84	9.02	0.11	2.30	1.73	18.24	16.93	2.28	1.20	16.93	17.53	18.52	3.94	16.93	16.93
	Q70	15.63	12.87	26.94	0.82	9.19	0.15	2.19	1.82	17.54	16.29	4.00	1.35	16.29	16.75	17.72	3.27	16.29	16.29
	R97	15.33	10.69	20.42	1.66	9.80	6.00	1.71	2.70	13.09	11.85	3.45	0.25	11.85	17.14	18.67	4.18	11.85	11.85
	I97	19.11	13.34	30.22	0.86	9.21	0.01	2.29	1.98	19.76	18.47	2.66	1.07	18.47	18.21	19.27	4.42	18.47	18.47
	C99	16.38	9.78	3.12	0.41	8.82	3.86	1.28	2.05	3.35	2.46	2.81	4.02	2.46	14.18	15.64	2.95	2.46	2.46
	R114	21.12	13.45	29.90	0.82	8.89	0.02	2.18	1.94	19.64	18.30	4.10	1.15	18.30	16.51	17.53	4.94	18.30	18.30
	L276	19.11	13.34	30.16	0.86	9.21	0.01	2.29	2.01	19.73	18.45	2.67	1.07	18.45	18.21	19.27	4.41	18.45	18.45
	rs2517713	19.13	13.17	29.95	0.82	9.24	0.01	2.30	1.98	19.57	18.29	2.66	1.11	18.29	18.20	19.25	4.37	18.29	18.29
	rs9260734	18.36	12.61	31.41	0.38	8.05	0.12	3.20	1.55	21.35	20.10	4.21	0.48	20.10	16.09	17.20	5.31	20.10	20.10
	rs3869062	24.60	12.58	32.86	0.29	7.71	0.22	3.16	1.82	22.52	21.20	6.98	0.67	21.20	15.61	16.72	7.34	21.20	21.20
	rs5009448	21.35	13.40	32.18	0.36	8.45	0.09	1.74	1.66	21.83	20.51	5.28	0.48	20.51	15.42	16.55	5.63	20.51	20.51
	rs16896923	23.22	12.52	37.16	0.98	8.51	0.49	2.96	1.99	24.47	23.12	6.48	0.26	23.12	16.49	17.83	6.83	23.12	23.12
TRIM31	rs2517664	NA	6.79	41.41	1.52	14.09	0.92	2.96	3.45	25.97	24.94	2.67	0.20	24.94	18.25	19.76	2.13	24.94	24.94
TRIM39	rs117495548	15.16	NA	42.88	0.84	13.36	1.14	2.50	3.28	28.12	26.97	6.40	0.16	26.97	19.52	21.63	6.17	26.97	26.97
Top	rs9391681	16.48	9.72	NA	0.59	8.20	3.96	1.33	2.16	2.16	1.44	2.93	4.72	1.44	13.16	14.65	2.78	1.44	1.44
HLA-C	C*07:02	20.56	12.26	45.59	NA	14.01	1.16	2.62	3.26	29.47	28.28	6.57	1.03	28.28	23.19	24.83	7.95	28.28	28.28
	C*03:04	21.15	12.73	41.48	2.27	NA	0.67	2.94	4.01	24.12	22.92	8.01	0.99	22.92	8.93	10.55	9.71	22.92	22.92
	C*03:02	19.93	12.20	48.00	0.77	12.39	NA	2.33	2.99	31.99	30.79	5.71	0.30	30.79	20.28	21.87	6.55	30.79	30.79
	C*12:03	20.56	12.37	44.89	1.14	13.35	1.20	NA	3.22	28.51	27.33	4.18	0.01	27.33	21.89	23.46	7.53	27.33	27.33
	C*12:02	20.46	12.48	45.01	1.15	13.77	1.22	2.58	NA	28.28	27.09	3.61	0.05	27.09	22.40	24.00	7.56	27.09	27.09
	C*01:02	17.39	11.22	20.38	0.94	7.42	4.21	1.34	1.76	NA	0.07	2.66	6.70	0.07	11.91	13.40	2.52	0.07	0.07
	M248	17.53	11.25	21.01	0.95	7.43	4.19	1.36	1.77	1.81	NA	2.71	6.69	NA	11.99	13.49	2.56	NA	NA
	W156	17.77	12.63	43.09	1.59	14.81	0.94	0.28	0.36	26.35	25.19	0.00	0.31	25.19	24.32	25.94	4.79	25.19	25.19
	E152	20.24	12.36	49.48	1.69	13.49	1.46	2.46	3.10	35.09	33.90	6.15	NA	33.90	22.52	24.72	7.34	33.90	33.90
	C99	17.53	11.25	21.01	0.95	7.43	4.19	1.36	1.77	1.81	NA	2.71	6.69	NA	11.99	13.49	2.56	NA	NA
	L95	18.04	11.12	38.50	3.53	0.16	0.38	3.46	4.62	20.49	19.34	9.54	2.16	19.34	NA	2.40	6.14	19.34	19.34
	I95	17.96	11.56	38.34	3.59	0.17	0.35	3.44	4.63	20.31	19.18	9.58	2.83	19.18	0.26	NA	5.96	19.18	19.18
	N77	16.71	11.64	42.08	1.99	15.48	0.72	2.96	3.61	25.29	24.12	3.76	0.59	24.12	20.10	21.54	0.00	24.12	24.12
	F9	17.53	11.25	21.01	0.95	7.43	4.19	1.36	1.77	1.81	NA	2.71	6.69	NA	11.99	13.49	2.56	NA	NA
	C6	17.53	11.25	21.01	0.95	7.43	4.19	1.36	1.77	1.81	NA	2.71	6.69	NA	11.99	13.49	2.56	NA	NA
HLA-B	B*58:01	19.92	12.19	47.90	0.76	12.36	0.11	2.33	3.00	31.88	30.68	5.69	0.31	30.68	20.24	21.83	6.52	30.68	30.68
	B*13:01	21.27	12.77	41.91	1.81	1.90	0.71	2.93	3.13	25.01	23.71	6.94	0.59	23.71	9.51	10.56	8.90	23.71	23.71
	B*46:01	16.51	11.27	11.07	1.14	7.47	4.45	1.34	1.92	0.04	0.43	2.66	7.09	0.43	11.93	13.36	2.30	0.43	0.43
	B*55:02	19.99	12.06	42.98	1.65	13.30	1.16	0.19	3.35	31.31	30.61	4.01	0.26	30.61	19.90	21.27	7.81	30.61	30.61
	B*38:02	19.82	12.45	48.17	3.23	12.32	1.55	2.38	2.99	31.82	30.61	5.70	0.32	30.61	20.09	21.68	6.59	30.61	30.61
	B*39:01	20.06	12.19	43.73	0.07	13.36	1.17	2.56	3.11	27.38	26.16	6.18	0.57	26.16	22.02	23.63	7.68	26.16	26.16
	W156	17.33	11.91	17.81	1.00	7.25	4.50	1.35	1.95	3.20	2.27	2.68	6.94	2.27	14.35	15.98	5.51	2.27	2.27
	L145	15.88	12.77	41.23	2.00	2.70	0.64	2.98	3.21	24.28	23.02	3.75	0.72	23.02	10.60	11.68	5.91	23.02	23.02
	L116	15.81	12.75	37.73	2.77	3.30	0.32	1.75	3.63	30.52	29.11	2.38	1.35	29.11	11.40	12.28	5.92	29.11	29.11
	D114	16.27	11.86	29.40	3.22	4.59	0.66	2.15	1.16	15.00	13.78	2.33	1.53	13.78	11.21	11.81	5.52	13.78	13.78
	R97	14.72	10.92	38.14	2.15	16.31	0.34	2.28	0.70	25.95	24.77	2.17	0.44	24.77	19.92	21.91	3.39	24.77	24.77
	R69	16.50	11.27	10.99	1.14	7.45	4.47	1.34	1.92	0.03	0.46	2.65	7.11	0.46	11.91	13.34	2.29	0.46	0.46
	K66	16.50	11.27	10.99	1.14	7.45	4.47	1.34	1.92	0.03	0.46	2.65	7.11	0.46	11.91	13.34	2.29	0.46	0.46
	W-11	18.32	11.82	35.09	4.29	9.32	8.07	1.63	1.38	21.33	20.09	2.64	2.39	20.09	15.57	17.29	5.59	20.09	20.09
	L-16	16.66	12.34	34.83	3.28	3.93	0.09	2.01	0.80	25.95	24.60	1.29	0.71	24.60	10.34	12.05	6.02	24.60	24.60
	rs2894207	13.30	11.23	37.64	3.41	2.69	0.26	0.29	4.44	19.99	18.79	0.50	1.81	18.79	5.34	7.14	1.57	18.79	18.79
	rs9265975	17.32	11.08	39.78	2.30	1.44	0.44	3.38	2.15	21.97	20.70	6.71	1.23	20.70	4.18	4.94	3.66	20.70	20.70
	rs2523589	14.842	12.309	30.6	0.28	5.43	0.24	1.32	1.01	20.5	19.2	1.47	1	19.2	11.8	13.5	6.78	19.2	19.2
HLA-DQA1	rs9461780	19.932	11.77	43.8	0.83	11.9	0.93	2.5	3.08	28.5	27.4	6.69	0.12	27.4	19	20.6	6.8	27.4	27.4

	Variant 2 \ Variant 1	B*58:01	B*13:01	B*46:01	B*55:02	B*38:02	B*39:01	W156	L145	L116	D114	R97	R69	K66	W-11	L-16	rs2894207	rs9265975	rs2523589	rs9461780
		HLA-B																		HLA-DQA1
unadjusted $-\log_{10}P$		1.46	16.16	40.78	18.44	2.11	9.02	33.96	16.71	26.38	28.12	18.21	40.92	40.92	16.79	29.59	25.57	24.17	35.75	16.36
HLA-A	rs29230	0.41	14.00	33.94	18.94	1.40	8.65	28.77	15.02	24.29	25.13	18.52	34.08	34.08	17.21	25.74	22.52	20.83	34.82	15.99
	rs29232	0.25	12.41	28.41	17.39	5.28	8.43	23.89	11.88	21.33	17.10	17.41	28.53	28.53	21.55	25.74	21.98	20.42	25.24	14.08
	rs3129055	4.35	14.23	26.10	17.80	4.11	8.18	21.08	13.89	24.82	23.12	19.26	26.22	26.22	12.16	27.25	23.41	22.13	30.12	15.44
	rs2860580	0.01	11.30	27.46	18.33	1.19	8.42	23.22	9.88	19.36	21.49	15.63	27.58	27.58	15.19	21.39	18.34	19.74	28.90	15.39
	A*02:06	1.48	16.16	40.96	18.48	2.12	9.01	34.12	16.70	26.36	28.20	18.21	41.10	41.10	16.78	29.58	25.59	24.17	35.86	16.47
	A*02:03	1.62	16.06	41.85	18.57	1.57	8.99	34.82	16.55	26.30	30.31	17.83	41.99	41.99	16.33	29.36	25.34	23.89	38.35	16.21
	A*11:01	0.24	10.66	31.23	18.25	1.28	8.53	26.48	12.00	21.73	25.22	19.68	31.37	31.37	17.87	25.67	23.08	19.38	34.12	15.06
	A*33:03	0.52	15.74	43.69	18.34	2.28	8.95	36.78	16.25	25.73	27.57	17.53	43.84	43.84	22.34	29.19	25.13	23.57	35.06	16.06
	A*02:07	3.85	12.74	7.03	16.83	4.80	7.56	5.83	12.58	19.02	11.60	10.54	7.10	7.10	5.64	19.44	17.80	18.81	21.05	13.92
	Q62	0.06	11.16	26.38	18.20	0.91	8.15	22.20	9.52	18.59	20.51	15.05	26.51	26.51	14.63	20.79	17.73	18.97	28.77	15.00
	Q70	0.09	11.19	25.76	18.33	1.22	8.43	21.89	9.76	20.03	21.96	13.24	25.88	25.88	15.74	22.68	18.75	18.15	28.68	15.05
	R97	5.99	13.83	20.40	18.28	0.40	8.54	16.62	13.14	21.40	25.40	13.41	20.51	20.51	6.60	22.49	19.97	21.57	34.26	16.18
	I97	0.06	11.34	28.50	18.25	1.11	8.36	23.87	9.95	19.29	21.83	16.39	28.63	28.63	15.41	21.64	18.76	20.27	29.37	15.36
	C99	3.85	12.74	7.03	16.83	4.80	7.56	5.83	12.58	19.02	11.60	10.54	7.10	7.10	5.64	19.44	17.80	18.81	21.05	13.92
	R114	0.06	11.23	28.25	18.38	1.11	8.46	23.76	12.70	22.07	22.07	17.07	28.38	28.38	16.95	24.19	19.62	18.42	30.46	14.96
	L276	0.05	11.33	28.46	18.25	1.10	8.39	23.84	9.94	19.27	21.84	16.39	28.59	28.59	15.43	21.65	18.74	20.27	29.40	15.35
	rs2517713	0.05	11.31	28.28	18.27	1.13	8.37	23.78	9.92	19.25	21.83	16.30	28.41	28.41	15.43	21.57	18.72	20.23	29.23	15.41
	rs9260734	0.19	10.32	30.12	17.56	1.37	8.08	25.87	9.09	18.12	19.07	14.57	30.26	30.26	13.71	20.61	19.50	18.34	28.41	14.72
	rs3869062	0.29	10.08	31.40	17.60	1.53	8.16	27.09	11.57	20.93	20.71	17.04	31.54	31.54	15.20	22.77	22.52	18.49	30.20	14.39
	rs5009448	0.16	11.10	30.82	16.96	1.31	7.76	26.80	12.74	21.63	19.50	15.21	30.95	30.95	15.58	23.60	20.39	17.24	28.62	14.67
	rs16896923	0.57	10.92	34.28	17.90	1.90	8.51	29.52	12.12	22.15	24.13	17.59	34.40	34.40	15.04	24.30	22.42	19.08	31.11	15.37
TRIM31	rs2517664	1.01	17.43	36.33	18.32	1.56	8.94	30.40	11.40	21.05	23.18	11.25	36.46	36.46	14.36	25.24	17.20	20.69	29.23	16.00
TRIM39	rs117495548	1.23	16.64	39.50	18.26	2.25	8.89	33.41	17.19	26.82	27.51	16.48	39.63	39.63	16.13	29.55	24.27	22.72	35.65	15.69
Top	rs9391681	3.95	12.15	5.16	16.79	4.89	7.46	4.34	11.94	18.01	10.43	9.89	5.22	5.22	4.86	18.09	17.03	17.98	19.86	14.22
HLA-C	C*07:02	1.26	16.82	40.93	18.79	4.26	8.73	33.98	17.54	27.86	29.94	19.18	41.07	41.07	19.75	31.54	27.64	25.24	35.28	16.25
	C*03:04	0.75	5.89	35.65	18.71	1.41	9.33	28.48	7.05	17.38	19.95	21.61	35.78	35.78	13.15	21.06	15.93	13.65	28.61	15.42
	C*03:02	0.31	15.70	43.43	18.36	2.29	8.92	36.71	16.19	25.67	27.59	17.48	43.58	43.58	22.94	28.80	24.83	23.54	35.01	16.04
	C*12:03	1.29	16.57	39.83	17.68	2.01	9.08	32.99	17.17	25.76	27.84	18.03	39.97	39.97	16.03	29.19	23.77	24.97	34.77	16.38
	C*12:02	1.33	16.19	39.76	18.59	1.99	9.02	32.93	16.81	26.86	26.40	16.11	39.89	39.89	15.21	27.66	26.79	23.35	33.97	16.34
	C*01:02	4.19	11.82	13.11	19.77	4.71	7.52	8.08	11.60	27.42	13.45	14.60	13.25	13.25	8.30	26.11	16.23	16.90	26.91	15.45
	M248	4.18	11.73	14.51	20.05	4.69	7.51	8.50	11.56	27.20	13.48	14.63	14.67	14.67	8.29	25.97	16.26	16.85	26.84	15.51
	W156	1.03	16.99	37.75	17.40	1.73	9.11	30.87	14.63	23.11	24.65	14.58	37.89	37.89	13.53	25.37	20.77	24.77	31.65	16.95
	E152	1.57	16.44	46.61	18.52	2.22	9.23	39.72	17.08	27.23	29.09	18.37	46.76	46.76	18.53	29.94	26.80	24.92	36.27	16.37
	C99	4.18	11.73	14.51	20.05	4.69	7.51	8.50	11.56	27.20	13.48	14.63	14.67	14.67	8.29	25.97	16.26	16.85	26.84	15.51
	L95	0.46	4.99	32.12	17.87	1.03	9.77	27.43	6.52	17.12	18.32	17.14	32.24	32.24	11.21	19.22	10.38	8.13	26.76	14.46
	I95	0.43	4.33	31.89	17.73	1.02	9.78	27.42	5.91	16.34	17.23	17.53	32.01	32.01	11.29	19.24	10.43	7.13	26.80	14.38
	N77	0.79	17.86	36.55	18.90	1.58	9.44	32.52	15.65	25.32	26.65	14.74	36.68	36.68	15.36	28.62	20.70	21.14	35.48	16.12
	F9	4.18	11.73	14.51	20.05	4.69	7.51	8.50	11.56	27.20	13.48	14.63	14.67	14.67	8.29	25.97	16.26	16.85	26.84	15.51
	C6	4.18	11.73	14.51	20.05	4.69	7.51	8.50	11.56	27.20	13.48	14.63	14.67	14.67	8.29	25.97	16.26	16.85	26.84	15.51
HLA-B	B*58:01	NA	15.66	43.53	18.35	2.30	8.94	36.82	16.15	25.62	27.51	17.40	43.67	43.67	23.28	28.72	24.77	23.50	34.90	16.02
	B*13:01	0.78	0.00	35.82	19.00	1.59	9.34	28.60	1.63	11.85	19.86	23.44	35.95	35.95	9.14	16.14	12.17	9.50	23.39	17.63
	B*46:01	4.65	11.18	NA	16.24	5.11	7.30	0.35	10.84	16.07	6.45	7.54	NA	NA	2.93	15.79	15.46	16.08	17.27	14.22
	B*55:02	1.25	17.01	37.09	NA	1.68	9.37	30.11	17.49	14.27	22.14	9.89	37.22	37.22	10.93	18.96	22.43	25.29	25.91	16.61
	B*38:02	1.66	15.74	43.39	18.23	NA	8.87	36.87	16.19	25.52	32.99	17.26	43.53	43.53	15.58	28.52	24.76	23.53	46.82	16.01
	B*39:01	1.31	16.59	38.44	18.74	1.87	NA	31.62	17.12	27.32	24.79	19.43	38.57	38.57	18.49	30.86	26.30	24.92	30.96	16.43
	W156	4.72	10.92	8.61	16.18	5.38	7.34	NA	10.62	16.00	6.42	7.85	8.74	8.74	3.07	16.41	15.21	15.94	17.84	14.74
	L145	0.70	1.15	35.00	18.95	1.47	9.33	27.81	NA	10.82	18.97	19.18	35.13	35.13	8.33	14.99	10.89	10.25	22.26	18.09
	L116	0.37	1.35	30.62	11.01	1.03	9.72	23.44	0.59	NA	13.12	9.81	30.73	30.73	4.22	6.59	9.03	10.18	14.45	18.21
	D114	0.65	8.20	20.24	14.94	7.46	6.66	12.78	7.80	11.88	NA	6.02	20.34	20.34	10.03	14.12	13.78	12.59	13.28	12.74
	R97	0.36	21.16	30.75	13.70	0.97	9.88	23.94	17.65	18.39	16.10	NA	30.86	30.86	5.30	17.06	20.39	20.15	22.80	15.66
	R69	4.67	11.17	NA	16.23	5.12	7.29	0.32	10.82	16.05	6.41	7.52	NA	NA	2.91	15.76	15.44	16.06	17.23	14.21
	K66	4.67	11.17	NA	16.23	5.12	7.29	0.32	10.82	16.05	6.41	7.52	NA	NA	2.91	15.76	15.44	16.06	17.23	14.21
	W-11	8.51	9.00	28.09	15.14	0.64	10.19	21.01	8.71	14.67	21.54	6.92	28.21	28.21	NA	14.92	15.65	15.56	24.71	17.29
	L-16	0.13	2.65	27.30	11.96	0.75	9.94	20.72	1.85	3.44	12.23	5.11	27.41	27.41	0.91	NA	8.36	9.98	10.05	16.83
	rs2894207	0.30	2.67	30.87	16.68	1.11	9.56	23.53	1.74	9.96	16.00	12.81	30.98	30.98	6.25	12.40	NA	4.57	18.60	16.89
	rs9265975	0.51	1.02	32.71	19.22	1.31	9.59	25.53	2.29	12.30	16.03	13.95	32.83	32.83	7.50	15.20	5.72	NA	21.27	16.44
	rs2523589	0.18	3.78	22.7	12.4	14.7	5.48	15.9	3.05	5.08	4.92	4.65	22.8	22.8	5.07	3.68	8.35	9.85	NA	15.53
HLA-DQA1	rs9461780	1.01	17.4	38.7	18.7	1.69	9.08	32.3	18.4	28.2	24.4	17.5	38.8	38.8	17.8	30.1	26.1	24.2	34.90	NA

* The conditional association analysis was performed using PLINK. The association of Variant 1, while controlling the effect of variant 2 (also adjusted for sex and age), is listed in the Table.
The six independent variants from this study are highlighted in red. Other variants were collected from previous publications listed below. NA means the calculation is not applicable. Color changes from red to blue indicate graded levels of strength to weak associations after conditional analysis.

References:

1 Tang, M. et al. The Principal Genetic DetermiNAts for Nasopharyngeal Carcinoma in China Involve the HLA Class I Antigen Recognition Groove. PLoS Genet 8, e1003103 (2012).
2 Chin, Y.M. et al. HLA-A SNPs and amino acid variants are associated with nasopharyngeal carcinoma in Malaysian Chinese. Int J Cancer 136, 678-87 (2015).
3 Bei, J.X. et al. A genome-wide association study of nasopharyngeal carcinoma identifies three new susceptibility loci. Nat Genet 42, 599-603 (2010).
4 Tse, K.P. et al. Genome-wide association study reveals multiple nasopharyngeal carcinoma-associated loci within the HLA region at chromosome 6p21.3. Am J Hum Genet 85, 194-203 (2009).
5 Tian, W. et al. Sequence-based typing of HLA-A gene in 930 patients with nasopharyngeal carcinoma in HuNA province, southern China. Tissue Antigens 86, 15-20 (2015).
6 Hildesheim, A. et al. Association of HLA class I and II alleles and extended haplotypes with nasopharyngeal carcinoma in Taiwan. J Natl Cancer Inst 94, 1780-9 (2002).

Supplementary Table 8: Eight independent signals for common variant association analysis after LD pruning.

Variants	Locus	EA/OA	MAF	Combined (*n*=5689)		Conditional analysis
				OR (95% CI)	*P*	*P*
**rs12174017[a]**	*HCP5B*	T/C	0.17/0.22/0.12	**2.09 (1.89-2.32)**	**1.75e-45**	**-**
**HLA-A amino acid Q_{62}**	*HLA-A*	P/Ab	0.33/0.26/0.39	**0.56 (0.51-0.61)**	**6.27e-44**	**2.22e-23**
**rs9405084[b]**	*HLA-B*	C/T	0.31/0.25/0.36	**0.60 (0.55-0.65)**	**1.47e-34**	**2.03e-19**
HLA-B*55:02	*HLA-B*	P/Ab	0.018/0.0047/0.031	**0.15 (0.10-0.23)**	**3.59e-19**	**5.53e-14**
**rs2517664**	*TRIM31*	T/C	0.030/0.014/0.046	**0.29 (0.22-0.37)**	**6.38e-21**	**9.73e-13**
**rs9265975**	*HLA-B*	A/G	0.10/0.072/0.13	**0.51 (0.45-0.58)**	**6.77e-25**	**3.12e-10**
**rs9461780**	*HLA-DQA1*	T/C	0.071/0.049/0.091	**0.52 (0.45-0.61)**	**4.38e-17**	**3.03e-14**
**rs117495548**	*TRIM39/ TRIM39-RPP21*	G/A	0.020/0.0094/0.030	**0.31 (0.23-0.43)**	**4.53e-13**	**2.05e-06**

MAF: minor allele frequency; OR: odds ratio; *P*: *P*-value
*Conditional *P* is calculated from the combined dataset.
LD pruning on the significant common variants was performed using PLINK (parameters: --indep-pairwise 200 50 0.8).
[a]r^2 = 0.84 with rs9391681; [b]r^2 = 0.91 with rs2523589

Supplementary Table 9: eQTL effects of rs2523589 and rs9265975 from GTEx portal.

SNP	Chr6 coordinate (hg38)	Gene Symbol	Normalized effect size	*P*	Tissues
**rs2523589**	31359557	*MIR6891*	0.34	1.4e-16	Whole Blood & 31 other tissues
		NOTCH4	0.35	5.0e-13	Whole Blood
		HCG4	0.35	1.8e-12	Whole Blood & 7 other tissues
		HCG27	0.24	2.9e-14	Thyroid & 28 other tissues
		HLA-C	-0.28	2.4e-12	Skeletal Muscle & 26 other tissues
		HLA-B	0.30	2.3e-10	Brain tissues & 5 other tissues
		ZBTB12	0.25	1.7e-9	Whole Blood
		MICA	0.17	1.3e-8	Whole Blood & skin
**rs9265975**	31348114	*LINC01149*	-0.42	5.7e-27	Whole Blood & 35 other tissues
		PSORS1C3	-0.62	1.8e-16	Artery, whole blood & 26 other tissues
		HCG27	0.14	9.0e-13	Whole Blood & 19 other tissues
		CCHCR1	0.23	9.8e-11	Esophagus, whole blood & 8 other tissues
		POU5F1	0.27	4.4e-8	Skeletal Muscle & 4 other tissues
		HLA-C	0.12	9.9e-5	Whole Blood and Adipose tissues

*https://gtexportal.org/home/

Supplementary Table 10: Comparison with previous NPC GWAS

	Other studies					In-house		1000Genomes
Studies	Population	SNPs	coordinates (hg38, chr6)	*P*	OR (95% CI)	In-house *P*	In-house OR (95% CI)	MAF 1000G
Tang et al. (2012)	Guangdong & Guangxi (2028 samples)	rs417162	29948728	1.05E-11	0.63 (0.53–0.75)	4.70E-35	0.59 (0.55-0.65)	C=0.3243/1624
		rs2517713	29950322	1.63E-11	0.60 (0.52–0.70)	1.69E-35	0.59 (0.55-0.65)	G=0.3243/1624
		rs9260734	29964889	2.63E-11	0.59 (0.50–0.69)	1.97E-34	0.58 (0.53-0.63)	A=0.2460/1232
		rs5009448	29972711	6.40E-11	0.61 (0.53–0.71)	1.22E-28	0.62 (0.57-0.68)	T=0.2674/1339
		rs2267633	29603064	1.89E-09	0.61 (0.52–0.72)	1.80E-22	0.64 (0.58-0.70)	G=0.1839/921
		rs29230	29608616	9.48E-09	0.61 (0.52–0.72)	5.67E-23	0.63 (0.58-0.69)	G=0.2133/1068
TSE et al. (2009)	Taiwan (1671 cases, 1287 controls)	rs2517713	29950322	3.90E-20	1.88 (1.65–2.15)	2.97E-35	0.59 (0.55-0.65)	G=0.3243/1624
		rs2975042	29952759	1.60E-19	1.86 (1.63–2.13)	1.12E-35	0.59 (0.55-0.64)	G=0.3227/1616
		rs9260734	29964889	6.77E-18	1.85 (1.61–2.12)	2.96E-34	0.58 (0.53-0.63)	A=0.2460/1232
		rs29232	29643654	8.97E-17	1.67 (1.48–1.88)	9.23E-27	1.50 (1.40-1.62)	G=0.2133/1068
		rs3869062	29967114	8.68E-16	1.78 (1.55–2.05)	3.44E-31	0.59 (0.54-0.64)	G=0.0940/471
		rs5009448	29972711	1.30E-15	1.72 (1.51–1.96)	1.22E-28	0.62 (0.57-0.68)	T=0.2674/1339
		rs3129055	29702484	7.63E-11	1.51 (1.34–1.71)	2.14E-22	1.46 (1.36-1.58)	G=0.2941/1473
		rs9258122 (rs3131866)	29703963	3.33E-10	1.49 (1.32–1.69)	1.75E-22	1.46 (1.36-1.58)	A=0.2939/1472
		rs16896923	30032910	2.49E-10	1.66 (1.42–1.94)	1.48E-19	0.64 (0.58-0.71)	C=0.0849/425
		rs2267633	29603064	1.28E-09	1.57 (1.36–1.82)	1.80E-22	0.64 (0.58-0.70)	G=0.1839/921
		rs2076483	29603768	1.49E-09	1.57 (1.36–1.82)	3.18E-22	0.64 (0.58-0.70)	G=0.1839/921
		rs29230	29608616	4.77E-09	1.56 (1.34–1.80)	5.67E-23	0.63 (0.58-0.69)	G=0.2133/1068
Bei et al. (2010)	Southern Chinese (5090 cases and 4957 controls)	rs2860580	29938914	4.88E-67	0.58 (0.55–0.62)	1.64E-37	0.58 (0.54-0.63)	A=0.3884/1945
		rs2894207	31295974	3.42E-33	0.61 (0.57–0.66)	2.69E-26	0.55 (0.49-0.62)	C=0.2268/1136
		rs28421666	32624960	2.49E-18	0.67 (0.61–0.73)	4.15E-04	0.84 (0.76-0.92)	G=0.0897/449
Chin et al. (2014)	Malaysian Chinese (481 controls, 444 cases)	rs3869062	29967114	1.73E-09	2.37 (1.79-3.14)	3.44E-31	0.59 (0.54-0.64)	G=0.0940/471
		rs2735085	29959318	1.92E-09	2.31 (1.76-3.03)	4.91E-36	0.57 (0.52-0.62)	A=0.2448/1226
		rs7747253	29952002	2.08E-09	2.21 (1.71-2.86)	4.77E-40	0.58 (0.53-0.62)	C=0.3920/1963
		rs2893999	29976055	2.61E-09	2.32 (1.76-3.07)	7.56E-31	0.59 (0.54-0.64)	C=0.0929/465
		rs9260734	29964889	2.96E-09	2.29 (1.74-3)	2.96E-34	0.58 (0.53-0.63)	A=0.2460/1232
		rs2860580	29938914	1.86E-08	2.04 (1.59-2.62)	1.64E-37	0.58 (0.54-0.63)	A=0.3884/1945
	rs28421666 is not validated in our dataset.							

Supplementary Table 11: Top 15 genes associated with NPC by SKAT gene association analysis.

Gene	N_INFORMATIVE	NumVar	NumPolyVar	Q	Pvalue
TRIM31	5689	301	301	1.90E+07	6.75E-29
AGPAT1	5689	282	282	5.45E+06	1.21E-27
HLA-B	5689	514	514	1.68E+07	1.60E-27
TRIM31-AS1	5689	284	284	1.91E+07	3.13E-27
MICA	5689	634	634	3.80E+07	2.44E-26
LINC01149	5689	200	200	8.11E+06	5.59E-23
MICA-AS1	5689	375	375	2.25E+07	2.71E-22
VARS	5689	406	406	4.34E+06	7.79E-22
HLA-H	5689	198	198	1.14E+07	2.58E-18
HSPA1L	5689	132	132	2.49E+06	3.23E-17
APOM	5689	118	118	1.27E+06	3.71E-17
MUCL3	5689	328	328	4.36E+06	6.07E-17
TRIM39	5689	384	384	2.62E+06	1.47E-16
GABBR1	5689	664	664	5.29E+06	6.95E-16
C2	5689	1103	1103	1.30E+07	1.94E-15

The top four genes associated with NPC identified by variant analysis are indicated in red font. NumVar stands for number of variants in the gene (or site) and the "NumPolyVar" stands for number of polymorphic genotypes. Biallelic variants have the same value for NumVar and NumPolyVar.

Supplementary Table 12: Clinical characteristics of NPC cases with positive TRIM31 expression in inflammatory cells

**Clinical parameters**	n = 52	%
**Median Age ± SD**	50 ± 11.03	
**Gender**		
**M**	43	82.7
**F**	9	17.3
**Stage**		
**I**	14	26.9
**II**	13	25.0
**III**	20	38.5
**IV**	5	9.6
**Early vs Late stage**		
**I+II**	27	51.9
**III +IV**	25	48.1
**Hospital**		
**PYNEH**	8	15.4
**QMH**	2	3.8
**TMH**	42	80.8
**Survival**		
**Alive**	22	42.3
**Dead**	30	57.7

Supplementary Table 13: Association of rare variants with NPC risk

Variants	Minor allele	Function	Gene	OR discovery	*P* discovery	OR validation	*P* validation	OR combined	*P* combined	H3K27ac	ORegAnno
rs17179220	A	intergenic	HLA-G;HCP5B	0.02	6.15E-16	0.03	9.67E-10	0.035	3.30E-22	No	Yes
rs117647774	A	intergenic	HCP5B;HLA-A	<0.024	2.18E-13	0.03	2.52E-10	0.025	5.78E-22	No	No
rs147023494	T	intergenic	HCP5B;HLA-A	0.02	6.15E-16	0.03	9.67E-10	0.035	3.30E-22	No	No
HLA-A	A*31:01	exonic	HLA-A	0.02	6.15E-16	0.03	1.89E-09	0.036	6.26E-22	-	-
rs148590958	C	intergenic	HLA-A;HCG9	0.02	3.21E-16	0.08	3.26E-08	0.035	9.17E-23	No	No
rs114716190	G	intergenic	HLA-A;HCG9	0.02	3.21E-16	0.08	3.26E-08	0.035	9.17E-23	Yes	No
rs116563937	G	intergenic	HCG27;HLA-C	<0.031	1.68E-10	<0.037	6.93E-09	<0.017	7.00E-19	No	No
rs140823445	G	intergenic	HCG27;HLA-C	<0.029	4.39E-11	<0.034	1.71E-09	<0.016	9.22E-20	No	No
rs7450349	A	intergenic	XXbac-BPG248L24.13;HLA-B	<0.023	1.13E-13	0.08	4.99E-09	0.036	1.19E-21	No	No
HLA-B	B*07:05	exonic	HLA-B	<0.027	5.94E-12	<0.033	8.52E-10	<0.015	5.83E-21	-	-
HLA-B	Baa156-R	exonic	HLA-B	<0.021	4.30E-15	0.02	2.86E-13	0.021	6.28E-26	-	-
rs2596478	C	intergenic	HLA-B;RNU6-283P	<0.021	4.30E-15	0.02	4.34E-12	0.022	8.54E-25	No	No
rs2523566	T	intergenic	HLA-B;RNU6-283P	<0.021	2.24E-15	0.02	4.34E-12	0.022	4.45E-25	No	No
rs2596429	A	intergenic	HLA-B;RNU6-283P	<0.021	2.24E-15	0.02	4.34E-12	0.022	4.45E-25	No	Yes
rs2523532	C	intergenic	AL671883.1;XXbac-BPG181B23.7	<0.021	4.30E-15	0.02	4.34E-12	0.022	8.54E-25	No	No
rs2523485	T	intergenic	AL671883.1;XXbac-BPG181B23.7	<0.021	2.24E-15	0.02	4.34E-12	0.022	4.45E-25	No	No
rs2523480	G	intergenic	AL671883.1;XXbac-BPG181B23.7	<0.021	4.30E-15	0.02	4.34E-12	0.022	8.54E-25	No	No
**rs2596540**	**A**	**UTR5**	**MICA**	**<0.020**	**3.19E-16**	**0.07**	**3.98E-10**	**0.032**	**1.03E-24**	**Yes**	**Yes**
rs2853971	A	ncRNA_intronic	HCP5	0.02	1.68E-16	0.07	3.98E-10	0.042	6.01E-24	No	No
rs3134791	A	ncRNA_intronic	HCP5	0.02	1.68E-16	0.07	3.98E-10	0.042	6.01E-24	No	No
rs2516450	C	ncRNA_intronic	HCP5	0.02	1.68E-16	0.07	3.98E-10	0.042	6.01E-24	No	No
rs2516469	G	ncRNA_intronic	HCP5	0.02	1.68E-16	0.11	3.01E-08	0.062	8.84E-19	No	No
rs11754929	T	ncRNA_intronic	HCP5	0.03	1.58E-12	0.10	1.31E-06	0.042	2.51E-18	No	No
rs115138709	C	ncRNA_intronic	HCP5	0.03	1.58E-12	0.10	1.31E-06	0.042	2.51E-18	Yes	No
rs77803816	A	ncRNA_intronic	HCP5	0.03	1.58E-12	0.10	1.31E-06	0.042	2.51E-18	Yes	Yes
rs11557308	T	ncRNA_exonic	HCP5	0.03	3.07E-11	0.10	1.31E-06	0.056	2.39E-17	Yes	No
rs150934595	T	ncRNA_intronic	HCP5	0.03	1.58E-12	0.10	1.31E-06	0.042	2.51E-18	No	No

The variants with similar D′ scores are highlighted with the same color.

Supplementary Table 14: Association of candidate variants from Henan ESCC and Hong Kong non-NPC cancer

Variants	Combined *P* in Hong Kong NPC	Henan (case n = 288 vs control n = 293)		Hong Kong non-NPC (n=405)*	
		P*	**OR**	***P****	**OR**
rs9391681	2.09E-46	0.04	0.56 (0.32-0.96)	0.90	1.02 (0.8-1.28)
HLA-A amino acid Q^{62}	1.31E-43	0.43	1.1 (0.86-1.41)	0.88	0.99 (0.84-1.16)
rs2523589	3.00E-36	0.13	1.2 (0.95-1.52)	0.29	0.91 (0.77-1.08)
*HLA-B*55:02*	3.24E-19	NA	NA	NA	NA
rs2517664	5.64E-21	0.16	1.28 (0.91-1.79)	0.98	0.99 (0.67-1.47)
rs9265975	7.74E-25	0.22	0.83 (0.62-1.12)	0.96	1.01 (0.8-1.27)

OR: odds ratio; *P*: *P*-value, logistic regression association adjusted for sex and age
*This Hong Kong non-NPC cancer cohort consisted of 384 esophageal squamous cell carcinoma (ESCC), 16 colorectal cancer, 3 non-small cell lung cancer, and 2 ovarian cancer patients.

Supplementary Table 15: Primers for Sanger sequencing validation

Primers	F' (5'-3')	R' (5'-3')	Amplicon length (bp)	Amplicon start	Amplicon end
Primer1	CTGTCCAGCTCTTGCCCTC	TTTGGATTGGAGCCAGCACT	217	29891331	29891547
Primer2	TCTGACAACACCACATTCCACA	CTGTGGGTGTAAACGTGGGA	477	29915895	29916371
Primer3	TACAGAGCTTCAAGAGTGGC	GGGTGTTAACATGGAATTGAGGC	655	29948113	29948767
Primer4	ACAAGACTCAAGATAGAGGTTCC	CATTTCCAAATAATCAGGAATGGCA	502	29951451	29951952
Primer5	ACCTATCTTGGAGGATAGTTGTGGG	CTTCACTCCTAGAGACAGGGAGG	285	30104648	30104932
Primer6	TTGGCTGAGGAATCGGCATT	AGGCACTCTTTGTCCTGAGC	778	30105048	30105825
Primer7	CTTCTAGAGTCCCAGTCTCTGC	GCATATTGCAACACATGCACCAG	433	30334244	30334676
Primer8	AATGGGTTTCAATGGGTCCAGA	TTCAAGGACACTGACTGATTCTTT	254	30383124	30383377
Primer9	GTGGCTCTATCTCGGCTCAC	GGAGGAATGGGGACTGTTGG	333	31204451	31204783
Primer10	ACTTCCTTGGCTATCGTTTTAAGC	TCCTGTTTCTGGACTTTTATTTGC	603	31253058	31253660
Primer11	AGAAACCCAGAATGCAAAGAATCCC	GCCAATTTGTAAAGTGGTATGGCA	650	31359232	31359881
Primer12	CCGTCTTTCCCTCCATTCCC	TCCTTCTGCCCATCACTTGG	815	31462661	31463475
HLA-A	TGGCCCCYGGTACCCGT	GAAACSGCCTCTGYGGGGAGAAGCAA	910	-	-

Y = C or T; S = G or C
