# Supplementary material for: Nasopharyngeal carcinoma MHC region deep sequencing identifies HLA and novel non-HLA TRIM31 and TRIM39 loci
Source: Commun Biol. 2020 Dec 11;3:759. doi: 10.1038/s42003-020-01487-y (PMC7733486; doi:10.1038/s42003-020-01487-y)
Supplement: Supplementary file 2 — Description of Additional Supplementary Files [file 42003_2020_1487_MOESM2_ESM.pdf]

Description of additional supplementary items

File name: Supplementary Data 1

Description of individual worksheets

Appendix A: Details of the proxy variant analysis for 2967 variants after controlling the eight index variants.

Controlling rs9391681: Source data for Figure 2a.

Controlling HLA-A aaQ62: Source data for Figure 2b.

Controlling rs2523589: Source data for Figure 2c.

Controlling rs2517664: Source data for Figure 2e.

Controlling rs9265975: Source data for Figure 2f.

rs117495548: Source data for Figure 2h.
