# Supplementary material for: Nasopharyngeal carcinoma MHC region deep sequencing identifies HLA and novel non-HLA TRIM31 and TRIM39 loci
Source: Commun Biol. 2020 Dec 11;3:759. doi: 10.1038/s42003-020-01487-y (PMC7733486; doi:10.1038/s42003-020-01487-y)
Supplement: Supplementary file 4 — Reporting Summary [file 42003_2020_1487_MOESM4_ESM.pdf]

nature research

Corresponding author(s): Prof. Maria Li Lung

Last updated by author(s): Oct 27, 2020

Reporting Summary

Nature Research wishes to improve the reproducibility of the work that we publish. This form provides structure for consistency and transparency in reporting. For further information on Nature Research policies, see our Editorial Policies and the Editorial Policy Checklist.

Statistics

For all statistical analyses, confirm that the following items are present in the figure legend, table legend, main text, or Methods section.

n/a	Confirmed	
☐	☒	The exact sample size (*n*) for each experimental group/condition, given as a discrete number and unit of measurement
☐	☒	A statement on whether measurements were taken from distinct samples or whether the same sample was measured repeatedly
☐	☒	The statistical test(s) used AND whether they are one- or two-sided<br>*Only common tests should be described solely by name; describe more complex techniques in the Methods section.*
☐	☒	A description of all covariates tested
☐	☒	A description of any assumptions or corrections, such as tests of normality and adjustment for multiple comparisons
☐	☒	A full description of the statistical parameters including central tendency (e.g. means) or other basic estimates (e.g. regression coefficient) AND variation (e.g. standard deviation) or associated estimates of uncertainty (e.g. confidence intervals)
☐	☒	For null hypothesis testing, the test statistic (e.g. *F*, *t*, *r*) with confidence intervals, effect sizes, degrees of freedom and *P* value noted<br>*Give P values as exact values whenever suitable.*
☒	☐	For Bayesian analysis, information on the choice of priors and Markov chain Monte Carlo settings
☒	☐	For hierarchical and complex designs, identification of the appropriate level for tests and full reporting of outcomes
☒	☐	Estimates of effect sizes (e.g. Cohen's *d*, Pearson's *r*), indicating how they were calculated

Our web collection on statistics for biologists contains articles on many of the points above.

Software and code

Policy information about availability of computer code

Data collection	no software was used
Data analysis	Sequencing reads were aligned to the human reference genome hg38 (hg38+Alt+decay) using Bwa.kit (https://github.com/lh3/bwa/tree/master/bwakit). The PCR duplicates were removed from the aligned file (bam) using SAMtools (version 1.3.1). Picard (version 2.2.1) was used to calculate the HsMetrics. The germline variants (SNP/InDels) were called using mpileup2snp from VarScan (version 2.3.8). at base resolution in the target region (pileup2cns) with parameters (minimum coverage: 8; minimum supported alternative reads: 2; minimum alternative allele frequency: 0.2; minimum average quality: 15; P threshold:0.01; minimum mapping quality 15). The function of the SNPs/InDels were annotated using ANNOVAR and functional trackers from UCSC Genome Browser. The classic HLA class I alleles were typed from cleaned FASTQ files using OptiType. The final variant sets (SNPs/InDels/HLA class I alleles/ HLA class I amino acid polymorphism) were imputed and phased using Beagle (version5.0). Linkage disequilibrium statistics (D' and r2) were calculated by PLINK(v1.9). The gene level burden test was performed using SNP-set kernel association test (SKAT) from Rvtests based on the NCBI RefSeq annotation (nPerm=10000, alpha=0.001, beta1=1, beta2=25). The common variants MAF ≥1% was analyzed by logistic regression model adjusted for sex and age to test the association between NPC and controls using PLINK. For the rare variants (MAF<1%), the Chi-square test or Fishers' exact test were used to test the association between NPC and controls. The Fishers' exact test was used when any of the expected values of the 2x2 contingency table was below 5.

For manuscripts utilizing custom algorithms or software that are central to the research but not yet described in published literature, software must be made available to editors and reviewers. We strongly encourage code deposition in a community repository (e.g. GitHub). See the Nature Research guidelines for submitting code & software for further information.

Data

Policy information about availability of data

All manuscripts must include a data availability statement. This statement should provide the following information, where applicable:

- Accession codes, unique identifiers, or web links for publicly available datasets
- A list of figures that have associated raw data
- A description of any restrictions on data availability

The sequencing data are deposited in the European Genome-phenome Archive (EGA), accession number EGAS00001003995.

Field-specific reporting

Please select the one below that is the best fit for your research. If you are not sure, read the appropriate sections before making your selection.

☒ Life sciences ☐ Behavioural & social sciences ☐ Ecological, evolutionary & environmental sciences

For a reference copy of the document with all sections, see nature.com/documents/nr-reporting-summary-flat.pdf

Life sciences study design

All studies must disclose on these points even when the disclosure is negative.

Sample size

We aimed to identified associated common variants (OR>1.5) and rare variants with high effect size(OR>20).
For rare variants, the power was calculated using power.fisher.test from R package statmod. If assume only 2 in controls and 40 in cases (OR around 20) from 1300 cases and 1300 controls will give a power>0.8, while using significant level 5e-8. For variants with higher frequency and similar effect size will have a even larger power.
For the common variants, the power was estimated using another R package (wp.logistic from WebPower). The allele frequency in the cases and controls are simulated with frequency range from 0.05 to 0.50 (increments 0.001). If OR>1.5 (or OR<1/1.5), the sample size (1300 cases and 1300 controls) will give a power large than 0.8.
Based on the calculation, we ensured the use of at least a sample size of 2600 in both discovery and validation phase (3047 samples in discovery phase and 2642 samples in the validation in the final analysis)

Data exclusions

At the sample level, we filtered out samples with insufficient sequencing coverage (<15X), a high off-target ratio, and high missing ratio (>10%). At the variant level, we filtered variants with high missing ratio (>10%). The variants that violated the Hardy Weinberg equilibrium (HWE) in the control samples were also filtered out (HWE P<1e-06). The InDels with length larger than 4 base pairs or multiple InDels in the same location are filtered out. The identity-by-descent test was performed using PLINK for variants in the MHC region to remove some potential related samples (PI_HAT>0.3). The principal components analysis (PCA) and multi-dimensional scaling (MDS) were used to check if there were any population stratification between cases and controls. The alignments of top variants were manually checked in the Integrative Genomics Viewer for some randomly selected samples.

Replication

This study is a two-phase study including discovery phase and validation phase.

Randomization

Samples are randomly coded in tissue bank. The samples for discovery and validation are randomly selected but balanced with sex and gender without knowing other clinical information.

Blinding

The investigators are blinded to group allocation. Samples stored in the tissue bank are collected independently.

Reporting for specific materials, systems and methods

We require information from authors about some types of materials, experimental systems and methods used in many studies. Here, indicate whether each material, system or method listed is relevant to your study. If you are not sure if a list item applies to your research, read the appropriate section before selecting a response.

Materials & experimental systems

n/a	Involved in the study	
☐	☒	Antibodies
☐	☒	Eukaryotic cell lines
☒	☐	Palaeontology and archaeology
☒	☐	Animals and other organisms
☐	☒	Human research participants
☒	☐	Clinical data
☒	☐	Dual use research of concern

Methods

n/a	Involved in the study	
☒	☐	ChIP-seq
☒	☐	Flow cytometry
☒	☐	MRI-based neuroimaging

Antibodies

Antibodies used	anti-TRIM31 antibody
Validation	anti-TRIM31 antibody (Proteintech, Cat#215711-AP) Application: Immunohistochemical (IHC) staining and Western blot (WB), Validation: WB, IHC

Eukaryotic cell lines

Policy information about cell lines

Cell line source(s)	361 [1] and 361-EBV, NPC43 [2] from Prof. Sai Wah Tsao<br>References:<br>1. Yip, Y. L., et al. Efficient immortalization of primary nasopharyngeal epithelial cells for EBV infection study. PLoS One 8, e78395 (2013). doi: 10.1371/journal.pone.0078395<br>2. Lin, W., et al. Establishment and characterization of new tumor xenografts and cancer cell lines from EBV-positive nasopharyngeal carcinoma. Nat Commun 9, 4663 (2018). doi: 10.1038/s41467-018-06889-5
Authentication	Short Tandem Repeat (STR) DNA profiling
Mycoplasma contamination	All cell lines tested negative for mycoplasma
Commonly misidentified lines<br>(See ICLAC register)	Our study did not use commonly misidentified lines.

Human research participants

Policy information about studies involving human research participants

Population characteristics	NPC cases and healthy controls were age and gender matched. Both cases and controls were Hong Kong Chinese.
Recruitment	In total, 2759 NPC patients were recruited, since 2009 to 2018, from five Hong Kong public hospitals including Queen Mary Hospital (QMH), Queen Elizabeth Hospital (QEH), Tuen Mun Hospital (TMH), Pamela Youde Nethersole Eastern Hospital (PYNEH) and Princess Margaret Hospital (PMH). Study protocols were approved by the Hospital Institutional Review Board and written consents were obtained from all patients. The 2939 control population consisted of 2422 healthy controls collected from the Red Cross and 517 cancer-free hospital controls from QMH. Patients enrolled with routine staging procedures included physical examination and imaging tests. Staging was defined according to the American Joint Committee on Cancer (AJCC) TNM system. Our study design is a retrospective case-control association study with inherent limitation of reverse causation. The alleles identified may or may not be the causal factors for NPC development resulting from indirect markers in linkage disequilibrium with the causal signals.
Ethics oversight	Institutional Review Board (IRB) of the University of Hong Kong/Hospital Authority Hong Kong West Cluster (HKU/HA HKW IRB)

Note that full information on the approval of the study protocol must also be provided in the manuscript.
